# Highly Boosting Circularly Polarized Luminescence of Chiral Metal–Imidazolate Frameworks

**DOI:** 10.1002/advs.202207333

**Published:** 2023-04-18

**Authors:** Xue‐Zhi Wang, Chuang‐Wei Zhou, Ji Zheng, Zhao‐Xia Lian, Meng‐Ying Sun, Yong‐Liang Huang, Dong Luo, Yan Yan Li, Xiao‐Ping Zhou

**Affiliations:** ^1^ College of Chemistry and Materials Science and Guangdong Provincial Key Laboratory of Functional Supramolecular Coordination Materials and Applications Jinan University Guangzhou 510632 P. R. China; ^2^ Department of Radiology The First Affiliated Hospital of Jinan University Guangzhou 510632 P. R. China; ^3^ Department of Medicinal Chemistry Shantou University Medical College Shantou 515041 P. R. China; ^4^ Key Laboratory of Biomaterials of Guangdong Higher Education Institutes Engineering Technology Research Center of Drug Carrier of Guangdong Department of Biomedical Engineering Jinan University Guangzhou 510632 P. R. China

**Keywords:** chirality, circularly polarized luminescence, metal–organic frameworks, subcomponent self‐assembly, supramolecular interactions

## Abstract

To develop a simple and general method for improving the circularly polarized luminescence (CPL) performances of materials is of great significance. In this work, two pairs of CPL‐active homochiral metal–organic frameworks (MOFs) P/M‐Et and P/M‐Et(Cd) with eta topology are reported. In comparison to the reported isomorphic Zn‐imidazolate MOFs P‐Me and M‐Me, both luminescence dissymmetry factor (*g*_lum_) and photoluminescence quantum yields (*Φ*_PL_) of P‐Et and M‐Et are largely improved by simply changing the methyl group to an ethyl group of ligands in P‐Et and M‐Et. Furthermore, the |*g*_lum_| values are significantly amplified up to 0.015 from 0.0057 by introducing the non‐luminescent halogenated aromatics, while an enhanced fluorescence efficiency is observed simultaneously (from 27.2% to 47.3%). The figure of merit value is about 40 times larger than that of P‐Me and M‐Me. Similarly, the CPL performances of P/M‐Et(Cd) are improved by about five times after encapsulating fluorobenzene molecules. This work represents a new and simple method for developing CPL‐active MOF materials.

## Graphical abstract

Two pairs of circularly polarized luminescence (CPL)‐active homochiral metal–organic frameworks (MOFs) with eta topology are reported. Their CPL performances are highly boosted by introducing ethyl substituent groups and non‐luminescent halogenated aromatics guests, and the figure of merit value is about 40 times larger than that of the chiral MOFs with methyl substituent groups.

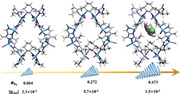

## 1 Introduction

Circularly polarized luminescence (CPL) has exhibited widespread potential applications, such as optical information storage,^[^ ^1^ ^]^ spintronics devices,^[^ ^2^ ^]^ encryption devices,^[^ ^3^ ^]^ molecular photoswitches,^[^ ^4^ ^]^ 3D displays,^[^ ^5^ ^]^ photodetectors,^[^ ^6^ ^]^ and smart sensors/probers.^[^ ^7^, ^8^ ^]^ An ideal CPL material should simultaneously show a large luminescence dissymmetry factor (*g*_lum_) and a high photoluminescence quantum yield (*Φ*_PL_). A figure of merit (FM) has been introduced to evaluate the comprehensive quality of CPL emissive systems,^[^ ^9^ ^]^ defined by the equation of FM = *g*_lum_ × *Φ*_PL_. Due to the trade‐off between *g*_lum_ and *Φ*_PL_, CPL materials with large FM values are still limited so far.

Metal–organic frameworks (MOFs) combining both metal ions/clusters and organic ligands have attracted much attention due to their tunable structure and advanced functions.^[^ ^10^, ^11^, ^12^, ^13^, ^14^, ^15^, ^16^, ^17^, ^18^ ^]^ When the chirality is endowed, the functions including asymmetric catalysis and enantioselective recognition or separation can be introduced into MOF materials. Although luminescent chiral MOFs have been reported extensively, their CPL properties and mechanisms are rarely investigated.^[^ ^19^, ^20^, ^21^, ^22^, ^23^, ^24^, ^25^, ^26^, ^27^, ^28^, ^29^, ^30^, ^31^, ^32^, ^33^, ^34^, ^35^, ^36^, ^37^ ^]^ For example, the first CPL MOF material based on transition metal ions was reported in 2019.^[^ ^25^ ^]^ After decorating chiral emitters on the surface of ZIF‐8 by molecular exchange, the chiral ZIF‐8 was CPL‐active with a |*g*_lum_| value of 5.5 × 10^−3^. Due to the porous advantage of MOFs, the straightforward strategy for improving the CPL performance of MOFs is the encapsulation of luminophores (e.g., dyes and quantum dots) into their cavities, which have been documented by several reported CPL MOFs.^[^ ^19^, ^20^, ^22^, ^23^, ^24^, ^26^, ^27^, ^28^, ^29^, ^30^, ^34^ ^]^ However, this method will change the emission color of the resulting system and the main luminescence may originate from the guest luminophores. Thus, it is essential to find a method to boost the CPL performance of MOFs at the same time without changing their luminescence origin.

**Scheme 1:**
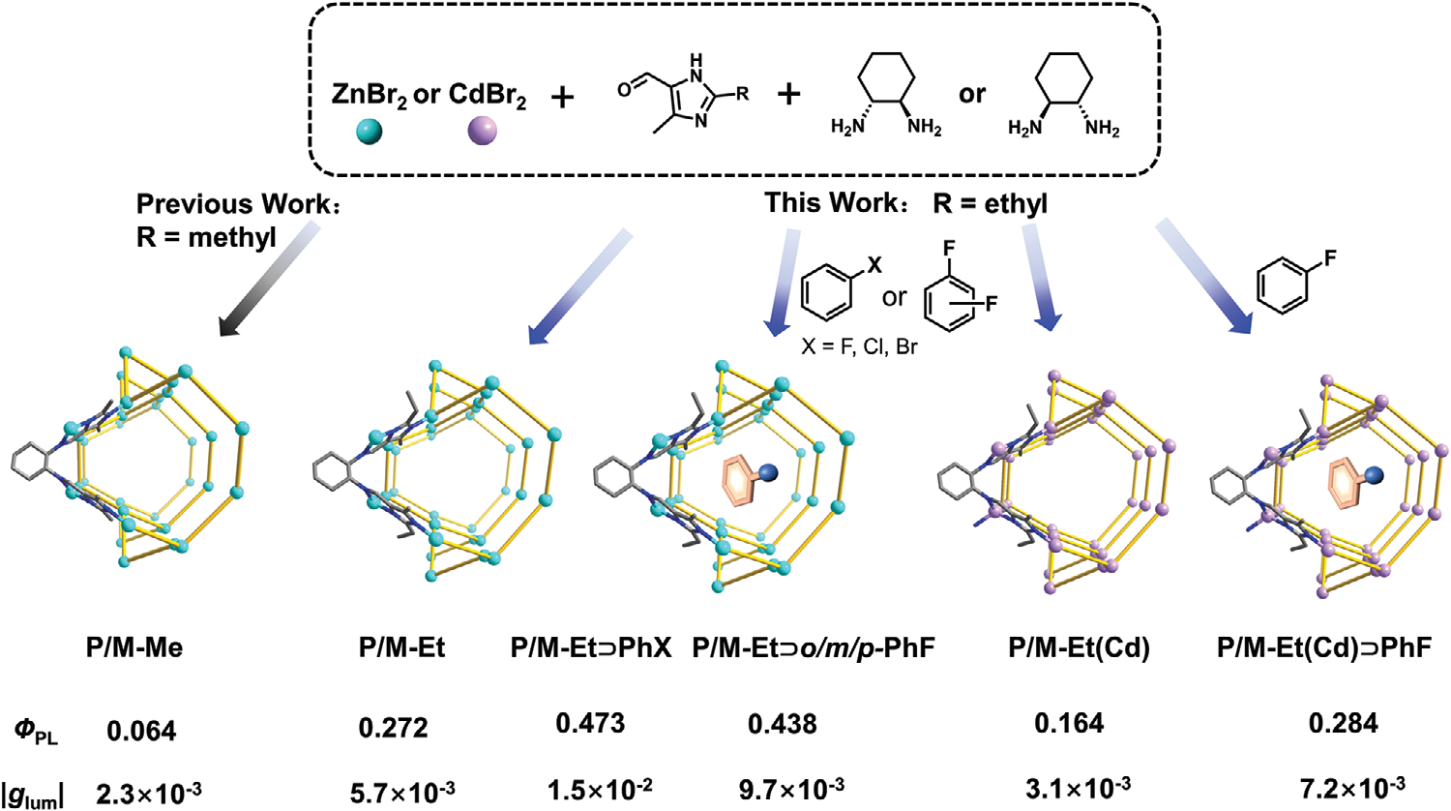
Subcomponent self‐assembly of chiral Zn/Cd‐imidazolate MOFs, encapsulation of different guest molecules, and their CPL performances.

In our previous work, a pair of CPL‐active Zn‐imidazolate MOFs, P‐Me and M‐Me (**Scheme** **1**) with eta topology were constructed by chiral and non‐luminescent imidazole ligands with Zn^II^ ions.^[^ ^33^ ^]^ However, their FM values of about ±1.5 × 10^−4^, based on the dissymmetry factors of ±2.3 × 10^−3^ and *Φ*_PL_ of 6.4% at 520 nm, are relatively low. Herein we report a pair of isomorphic chiral MOFs P‐Et and M‐Et by simply changing the substituent group methyl to ethyl, which achieved distinct CPL properties compared with MOFs P‐Me and M‐Me (Scheme 1). CPL measurements showed the enhanced |*g*_lum_| values of 5.7 × 10^−3^ for P‐Et, while *Φ*_PL_ (27.2%) was about four times larger than that of P‐Me. The amplification of the |FM| values by one order of magnitude was observed from 10^−4^ to 10^−3^. By filling the channels of MOFs P/M‐Et with non‐luminescent halogenated aromatics (e.g., fluorobenzene, chlorobenzene, bromobenzene, and *o/p/m*‐difluorobenzene denoted as P/M‐Et⊃PhX and P/M‐Et⊃*o/p/m‐*PhF_2_, Scheme 1), both |*g*_lum_| and *Φ*_PL_ were further improved (e.g., |g_lum_| = 1.5 × 10^−2^, and *Φ*_PL_ = 47.3%_)_, giving excellent |FM| values (6.1 × 10^−3^), while the emission wavelength and lifetime are not significantly changed. This FM value is about 40 times larger than the value of P/M‐Me. Theoretical calculation studies found that the encapsulated halogenated aromatics interact with the framework via multi weak interactions to reduce the nonradiative decay process and improve the *Φ*_PL_. Moreover, after loading the halogenated aromatics, the angle between magnetic (***m***) and electric (***μ***) transition dipole moments (*θ_*μ*,m_*) is enlarged, leading to the enhancement of |*g*_lum_|. For comparison, isomorphic Cd‐imidazolate MOFs P/M‐Et(Cd) were synthesized and characterized. Both P/M‐Et(Cd) showed enhanced circularly polarized fluorescence properties after encapsulating fluorobenzene molecules. This work provides new and simple strategies to boost the CPL performance of MOF materials.

## 2 Results and Discussion

MOFs P‐Et and M‐Et were synthesized by mixing 2‐ethyl‐5‐methyl‐1H‐imidazole‐4‐carbaldehyde, chiral 1,2‐diaminocyclohexane, and ZnBr_2_ under solvothermal conditions (for details, see the Supporting Information). P‐Et(Cd) and M‐Et(Cd) can be obtained by using the same synthesis method when ZnBr_2_ was replaced with CdBr_2_ (for details, see the Supporting Information). Phase‐pure rod–shaped crystals were obtained directly. In addition, we synthesized imidazole derivatives with an isopropyl group (2‐isopropyl‐4‐methyl‐1H‐imidazole‐5‐carbaldehyde, denoted as IMIMC) using a modification of a previously published method (for details, refer to the Supporting Information).^[^ ^38^ ^]^ However, the subcomponent self‐assembly of IMIMC, chiral 1,2‐diaminocyclohexane, and ZnBr_2_ under the same condition of synthesis of P‐Et and M‐Et to MOFs was not successful, which was probably due to the steric hindrance of the isopropyl groups.

**Figure 1:**
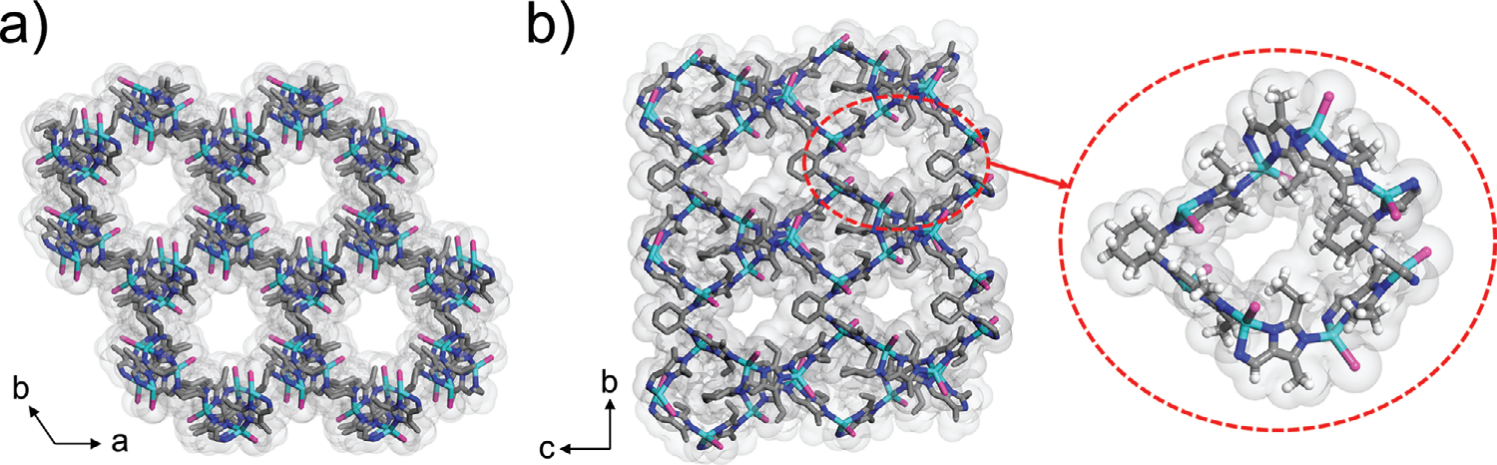
The crystal structure of P‐Et with Connolly surface (gray curved surface, a probe radius of 0.8 Å was used to visualize the channels) viewed along the a) *c*‐axis and b) *a*‐axis, Color codes: Zn, cyan; C, gray; Br, pink; N, blue; H, light gray.

X‐ray crystallography revealed that P‐Et and M‐Et crystallize in chiral space groups *P*6_1_ and *P*6_5_, respectively. P‐Et(Cd) and M‐Et(Cd) crystallize in *P*6_5_ and *P*6_1_, respectively. Two pairs of MOFs are enantiomers with eta topology like P/M‐Me. Hence only the structural description is given for P‐Et. The asymmetric unit of P‐Et contains four Zn(II), four Br, two *R*–*L* (*R*‐H_2_ *L* = *N*,*N*′‐((1*R*,2*R*)‐cyclohexane1,2‐diyl)bis(1‐(2‐ethyl‐5‐methyl‐1H‐imidazol‐4‐yl)methanimine)), and one lattice solvent molecules (Figure S1a, Supporting Information). The Zn^II^ ions possess a tetrahedral geometry and are bridged by imidazole units to fabricate trifold right‐handed extensive helixes, which are further linked by the cyclohexyl groups to generate a 3D chiral porous framework. P‐Et shows 3D open channels with two types: a helical channel along the *c*‐axis with a diameter of ≈15 Å and 1D channels along the *a/b*‐axis with a diameter of ≈10 Å (**Figure** **1**). The total potential solvent area volume is 4924 Å^3^ (42%) per unit cell for MOFs P‐Et, obtained by PLATON analysis.

The phase purity of the bulk samples of P/M‐Et and P/M‐Et(Cd) was confirmed by powder X‐ray diffraction (PXRD) experiments. The observed PXRD patterns are in good agreement with the simulated ones (Figures S2 and S17, Supporting Information). Thermogravimetric analysis (TGA) performed on as‐synthesized P/M‐Et revealed that these compounds have thermal stability up to ≈350 °C (Figure S3, Supporting Information). The thermal stability is also verified by in situ variable temperature PXRD of P‐Et that the framework remains crystalline over a wide temperature range (heated from 25 to 200 °C, Figure S4, Supporting Information), and remains stable after solvent removal (solvent escape ≈200 °C, confirmed by TGA). The PXRD studies revealed that P‐Et is stable in common organic solvents and water at room temperature for 24 h (Figure S5, Supporting Information). To study the porous property, the samples of P/M‐Et were measured by performing nitrogen adsorption experiments at 77 K (Figure S6, Supporting Information). As expected, both activated P/M‐Et show reversible typical type I isotherms for typical microporous materials. The Brunauer–Emmett–Teller surface areas were calculated as 693 and 558 m^2^ g^−1^ for P‐Et and M‐Et, respectively, which were smaller than that of P‐Me (811 m^2^ g^−1^) and M‐Me (843 m^2^ g^−1^).

**Figure 2:**
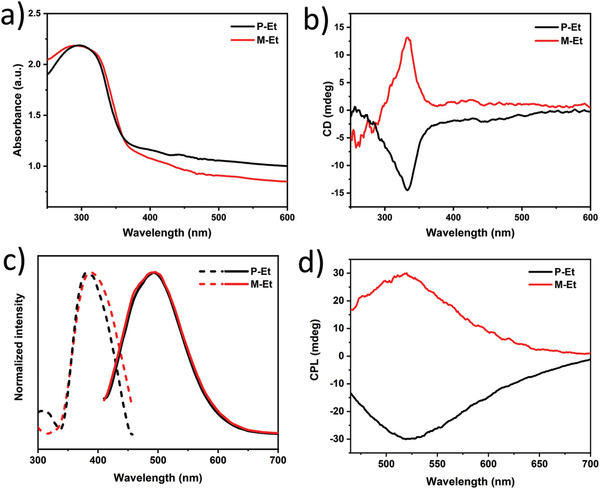
a) Solid‐state UV–vis absorption spectra, b) ECD spectra, and c) normalized excitation (dotted line) and emission spectra (solid line), and d) CPL spectra of P‐Et and M‐Et.

The solid–state photophysical properties of chiral MOFs were studied under an air environment. The solid‐state UV–vis absorption spectra of P/M‐Et and P/M‐Et(Cd) were identical, and absorption bands centered at around 300 nm were observed (**Figure** **2**a and Figure S18a, Supporting Information). The bands can be assigned to the *π*–π* transition of ligand R/S‐L. The solid‐state electronic circular dichroism (ECD) spectra of the two pairs of enantiomers, P/M‐Et and P/M‐Et(Cd), exhibited a mirror‐image relationship in the 250–450 nm range (Figure 2b and Figure S18b, Supporting Information). The homochirality of P‐Et and M‐Et was also further documented through solid‐state vibrational circular dichroism (VCD) spectra.^[^ ^39^ ^]^ Mirror‐image VCD signals were observed for P‐Et and M‐Et in the wavelength range of 1800–1000 cm^−1^, which corresponds well with the Fourier‐transform infrared spectra (refer to Figure S8, Supporting Information).

Both P‐Et and M‐Et emitted green light at 518 nm (Figure 2c and Table S3, Supporting Information) upon being excited at 395 nm. Similarly, P‐Et(Cd) and M‐Et(Cd) showed green fluorescence with a narrow band centered at 492 nm (Figure S19a and Table S4, Supporting Information). The decay lifetimes of P‐Et and P‐Et(Cd) were 1.78 and 1.08 ns, respectively, suggesting they emitted green fluorescence. The absolute *Φ*_PL_ of P‐Et is measured as 27.3% within an integrating sphere, which is evidently higher than the value of P‐Et(Cd) (16.4%) and reported imidazolate‐based frameworks without doping other emitters.^[^ ^40^, ^41^, ^42^ ^]^ Due to the chiral feature, we further investigated the circularly polarized luminescence of P/M‐Et and P/M‐Et(Cd) (Figure 2d; Figures S9 and S20, Supporting Information). Two couples of enantiomers showed strong mirror‐image CPL signals at 520 and 492 nm, respectively. The *g*_lum_ values of P‐Et and M‐Et were −5.7 × 10^−3^ and 5.1 × 10^−3^, corresponding to FM values of −1.6 × 10^−3^ and 1.4 × 10^−3^, respectively, which are about ten times larger than the reported values of P/M‐Me (±1.5 × 10^−4^). To the best of our knowledge, this is the first time that an order of magnitude increase in FM value can be achieved by simply changing substituents for MOF materials. We preliminarily speculate that this phenomenon is related to the increase in the rigidity of the chiral frameworks by replacing small methyl groups with relatively bulky ethyl groups to boost the CPL performance of MOF materials. On the other hand, the CPL performances of the P/M‐Et are also better than those of P/M‐Et(Cd) (|FM| = 4 × 10^−4^, see Table S3, Supporting Information). The CPL brightness (*B*_CPL_) has been also employed to evaluate the overall CPL performance.^[^ ^43^, ^44^ ^]^ Based on the calculation formula (*B*_CPL_ = *ξ*_abs_ × *Φ*_PL_ × |*g*_lum_|/2), the *B*_CPL_ values of P/M‐Et were determined as 17.8 × 10^−5^ and 16.8 × 10^−5^, which are higher than those of P/M‐Et(Cd) (4.0 × 10^−5^ and 4.5 × 10^−5^, respectively, Table S3, Supporting Information).

The porous and stable characterizations of P‐Et and M‐Et promote us to study how to further boost their CPL performances without changing their luminescent origin. The halogenated aromatic molecules fluorobenzene (PhF), chlorobenzene (PhCl), and bromobenzene (PhBr) are chosen for their non‐luminescent feature and possible formation of weak halogenated bonds with the frameworks that may boost the luminescence of host.^[^ ^45^ ^]^ Furthermore, the sizes of the halogenated aromatics match the channels of P/M‐Et along the *a*/*b*‐axis, which will probably help them to be encapsulated. TGA (Figure S10, Supporting Information) and elemental analysis (see the Supporting Information) demonstrated that halogenated aromatics exist in the products of the synthesis of P/M‐Et in the presence of PhX (PhX refers to halogenated aromatics). The locations of the halogenated aromatic molecules can be characterized by single‐crystal X‐ray crystallography. Unfortunately, we failed to obtain the detailed location of the guest molecules due to their serious disorder of the structures. PXRD patterns of the series of P‐Et⊃PhX were similar to that of the parents and no new diffraction peaks were observed (Figure S11, Supporting Information). The ECD spectra of P‐Et⊃PhX are similar and their |g_abs_| (≈3.5 × 10^−4^) values are closer to their parent MOF (≈2.2 × 10^−4^), indicating that the ground state chiroptical properties of the frameworks do not change significantly with the guests loaded (Figures S12 and S13, Supporting Information). The emission peaks (*λ*_max_ from 515 to 520 nm) of P/M‐Et⊃PhX show no significant shift compared with those of P/M‐Et (*λ*_max_ = 518 nm; Figure S14 and Table S3, Supporting Information) verifying no host–guest charge transfer. Furthermore, the emission lifetimes of P‐Et⊃PhX (1.79–2.04 ns) are very close to the parent P/M‐Et (1.74–1.78 ns), further suggesting that the guest halogenated aromatic molecules do not alter the luminescent origin. Interestingly, the *Φ*_PL_ values are highly enhanced after encapsulating halogenated aromatic. P/M‐Et⊃PhF has the larger *Φ*_PL_ values of 47.3% and 46.6% than that of P/M‐Et⊃PhCl and P/M‐Et⊃PhBr (≈40%). The reduction of fluorescent emission efficiency in P/M‐Et⊃PhCl and P/M‐Et⊃PhBr here may be related to heavy atoms of Cl and Br in PhCl and PhBr. Typically, the external heavy atom will enhance the singlet‐to‐triplet intersystem crossing, which quenches the fluorescence. Furthermore, P/M‐Et⊃*o/p/m‐*PhF_2_ containing difluorobenzene molecules have been synthesized and characterized, we found that their *Φ*_PL_ values (ranging from 36.3–43.8%) were also enhanced in comparison to the P/M‐Et.

**Figure 3:**
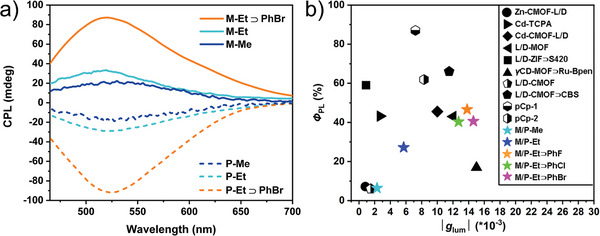
a) CPL spectra of P‐Me, P‐Et, P‐Et⊃PhF, M‐Me, M‐Et, and M‐Et⊃PhF. b) Summary of the |*g*_lum_| and *Φ*_PL_ values of this work and reported CPL‐active non‐lanthanide MOFs.

We further investigated the circularly polarized luminescence. The amplification of the |*g*_lum_| values by one order of magnitude was observed for P/M‐Et⊃PhX (**Figure** **3**a and Table S3, Supporting Information), which increase from 10^−3^ to 10^−2^ (±1.2–1.5 × 10^−2^) after loading PhX. Among them, M‐Et⊃PhBr gives the largest |*g*_lum_| value of 1.5 × 10^−2^, while, P‐Et⊃PhF has the max *Φ*_PL_ value of 47.3% and calculated |FM| value is up to 6.1 × 10^−3^, which enlarges about 40‐fold relative to P‐Me. The CPL performances of MOFs P/M‐Et⊃PhX are comparable to the best non‐lanthanide CPL MOFs (Figure 3b and Table S5, Supporting Information).^[^ ^19^, ^20^, ^21^, ^22^, ^23^, ^33^, ^34^, ^37^ ^]^ Similarly, the CPL performances of P/M‐Et⊃*o/p/m‐*PhF_2_ were also obviously enhanced in comparison to their parent P/M‐Et (Figures S11 and S13, and Table S3, Supporting Information). In contrast, the CPL performances of MOFs P/M‐Me do not show significant enhancement by loading the halogenated aromatics under the same conditions as those for M/P‐Et.

To further verify the versatility of this method, P/M‐Et(Cd)⊃PhF was synthesized and characterized. As expected, their CPL performances were also significantly improved after encapsulating the PhF molecule. P/M‐Et(Cd)⊃PhF exhibited the higher quantum yield (27.7% and 28.4%) and larger |*g*_lum_| value (6.8 × 10^−3^ and 7.2 × 10^−3^) in comparison to the P/M‐Et (Cd) (Table S4, Supporting Information). Both the |FM| and *B*_CPL_ values are increased by about four times (Figures S17–S20 and Table S4, Supporting Information).

**Figure 4:**
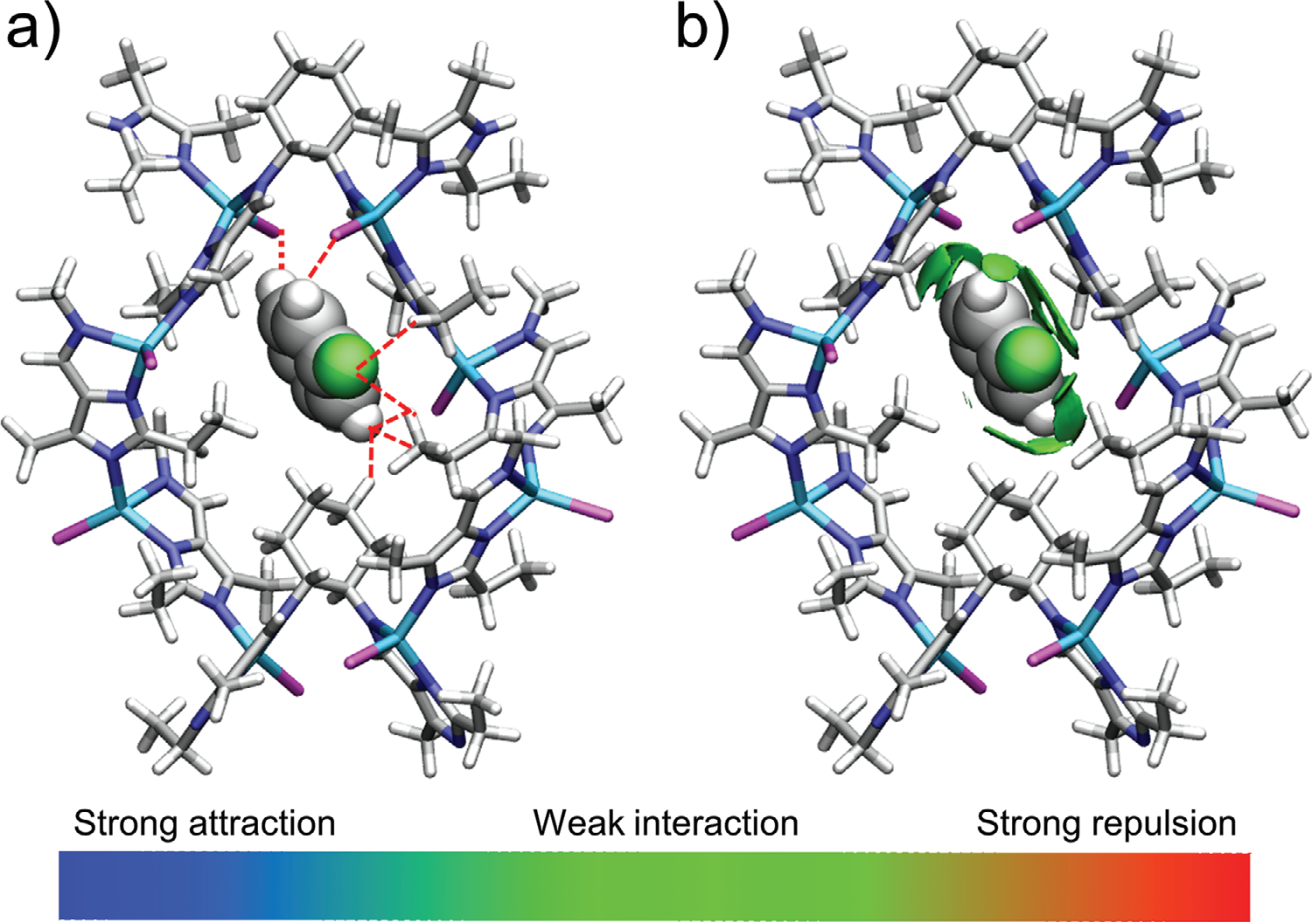
a) The calculation structure of PhF and the eight‐membered ring window of P‐Et (weak interactions are highlighted with red dashed lines). b) Independent gradient model based on Hirshfeld partition (IGMH) analysis between PhF and the eight‐membered ring window of P‐Et. The color scale shows a range of interaction strengths: strong attraction (blue), weak interaction (green), and strong repulsion (red). Color codes: Zn, cyan; C, gray; Br, pink; F, green; N, blue; H, light gray.

To understand the mechanism, density functional theory (DFT) calculations were performed. The PhF molecule was selected as an example and was filled in two types of channels of the parent P‐Et (Figure S21, Supporting Information), respectively. To optimize and calculate the energy of the complex, the CASTEP module of Materials Studio software was employed.^[^ ^46^ ^]^ We found that the total interaction energy of the P‐Et⊃PhF in the channels of the *a*‐axis (−4673.64 a.u.) was higher than that of the *c*‐axis (−4673.56 a.u.). These results revealed that PhF prefers to occupy the channels of the *a/b*‐axis. Therefore, an eight‐membered ring window of the *a/b*‐axis channel was selected as a structural model. The anchored location of PhF molecules in the window was further determined by DFT calculations (**Figure** **4**a).^[^ ^47^, ^48^ ^]^ As shown in Figure 4a, PhF exquisitely locates in the window, which may interact with the framework via multiple supramolecular interactions, including C—H···X (X = F, Br, and C) interactions and *π*···*π* stacking (Table S6, Supporting Information). Furthermore, the independent gradient model based on Hirshfeld partition (IGMH) analysis of this structural model showed that the PhF molecule was surrounded by green isosurface,^[^ ^49^, ^50^ ^]^ suggesting that there exist weak interactions between PhF and the P‐Et framework (Figure 4b). The calculated interaction energy is negative, indicating attractive interactions between host and guest. The total interaction energy is decomposed into Pauli repulsion, orbital interaction, electrostatic interaction, and dispersion interaction. The energy decomposition analysis results show that dispersion interaction (−33.69 kcal mol^−1^) plays a dominant role in the interaction energy (Table S7, Supporting Information).^[^ ^51^, ^52^, ^53^ ^]^ Therefore, we conclude that the dispersion interactions between the halogenated aromatic guests and the framework probably suppress the distortion and vibration relaxation of the excited state of the chiral MOFs thus enhancing their photoluminescence performance.

Time‐dependent DFT (TDDFT) calculations were performed for the octanuclear Zn(II) models to study the possibilities of intersystem crossing of the eight‐membered ring window (P‐Et) and bromobenzene guests in P‐Et⊃PhBr. The electron density difference maps of the first singlet excited state (*S*_1_) show ignorable contributions from guests of P‐Et⊃PhBr, indicating the absence of external heavy atom effects of halogen atoms (Table S9, Supporting Information). However, the calculation results still indicate the possibility of intersystem crossing (ISC) for these models because the energy of S_1_ is equal to that of the closest triplet excited state. In this stage, the ISC process in our system cannot be fully precluded, but those models with halobenzene guests do not show a larger possibility of ISC relative to the empty model (P‐Et), since the singlet–triplet energy difference is 0 eV for each model.

The intrinsic chiroptical activity is related to the arrangement of electric dipole and magnetic dipole,^[^ ^54^ ^]^ which can be studied by theoretical calculation. Theoretically, the dissymmetry factors can be calculated by the equation *g*_lum_ = 4(| ***μ*** | × | ***m*** | × cos*θ_*μ*,m_*)/(| ***μ*** |^2^ + | ***m*** |^2^) (*g*_lum_ ≈ 4cos*θ_*μ*,m_* | ***m*** |/| ***μ*** |, when ***μ*** is much larger than ***m*** *)*, where ***μ***, ***m***, and *θ_*μ*,m_* represent the electric, magnetic transition dipole moments, and the angle between ***μ*** and ***m***, respectively. TDDFT calculations were performed using the excited‐state structures to understand the origin that boosts the |*g*_lum_| values with PhF or *o*‐PhF_2_ loaded. The three parameters relative to *g*_lum_ were calculated based on the optimized geometries of the first excited state *S*_1_. As a result, calculated *θ_*μ*,m_* values were 103.3° for P‐Et, 111.8° for P‐Et⊃PhF, and 121.8° for P‐Et⊃*o*‐PhF_2_ suggesting that the *θ_*μ*,m_* was enlarged after introducing PhF or *o*‐PhF_2_. At the same time, the | ***μ*** | value was 2.06 × 10^−21^ esu cm for P‐Et⊃PhF, which was smaller than that of P‐Et (1.23 × 10^−20^ esu cm). Meanwhile, after loading the PhF, the | ***m*** | value of P‐Et⊃PhF (3.85 × 10^−23^ erg G^−1^) was close to the value of P‐Et (3.19 × 10^−23^ erg G^−1^). Thus, the theoretical |*g*_lum, cal_| value of the *S*_1_→*S*_0_ transition of P‐Et⊃PhF was calculated to be 2.78 × 10^−2^, an order of magnitude larger than the calculated value of P‐Et (2.38 × 10^−3^). After introducing *o*‐PhF_2_ into P‐Et, the | ***m*** | was significantly increased to 5.04 × 10^−23^ erg G^−1^ and | ***μ*** | was slightly increased to 2.55 × 10^−20^ esu cm. As a result, P‐Et⊃*o*‐PhF_2_ gave a certain increased |*g*_lum, cal_| value of −4.16 × 10^−3^. Both the |*g*_lum, cal_| values of P‐Et, P‐Et⊃PhF, and P‐Et⊃*o*‐PhF_2_ are the order of magnitude to the experimental results (Table S8, Supporting Information), suggesting that the enhancement of the CPL performance after introducing PhF or *o*‐PhF_2_ into P‐Et is mainly due to an increase in the value of *θ_*μ*,m_*.

## 3 Conclusions

In summary, we have successfully synthesized and characterized two couples of enantiomers chiral Zn/Cd‐imidazolate MOFs, P/M‐Et and P/M‐Et(Cd), decorated with ethyl groups. In comparison to the MOFs P/M‐Me, both the |*g*_lum_| and *Φ*_PL_ values of P/M‐Et are enhanced by simply changing methyl to ethyl. The |FM| value increases from 1.5 × 10^−4^ to 1.6 × 10^−3^ with one order of magnitude. Furthermore, by confining the non‐luminescent halogenated aromatics into the cavities of P/M‐Et, the simultaneous improvement of both |*g*_lum_| (order of 10^−2^) and *Φ*_PL_ (>40%) values is observed, and the FM value increases to 6.1 × 10^−3^, which is 40 times larger than that of P/M‐Me. This method also works in P/M‐Et(Cd) that the FM values of Cd‐imidazolate MOFs are improved by about four times after encapsulating fluorobenzene. Molecular simulations reveal that the halogenated aromatics show multi weak interactions with the frameworks, which is beneficial to the improvement of *Φ*_PL_ values. After loading the halogenated aromatics, TDDFT calculations demonstrate that the cos *θ_*μ*,m_* is enlarged, leading to a higher |*g*_lum_| value. By changing the substituent groups and encapsulating non‐luminescent guest molecules, the CPL performances of MOFs are highly boosted without significantly changing the emission wavelength and lifetime, which provides new and simple strategies for developing advanced CPL materials.

[CCDC 2 194 055, 2 194 056, 2 243 850, and 2 243 851 contains the supplementary crystallographic data for this paper. These data can be obtained free of charge from The Cambridge Crystallographic Data Centre via www.ccdc.cam.ac.uk/data_request/cif.]

## Conflict of Interest

The authors declare no conflict of interest.

## Supporting information

Supporting Information

Supporting Information

## Data Availability

The data that support the findings of this study are available in the supplementary material of this article.
