# Supplementary material for: Highly Boosting Circularly Polarized Luminescence of Chiral Metal–Imidazolate Frameworks
Source: Adv Sci (Weinh). 2023 Apr 18;10(17):2207333. doi: 10.1002/advs.202207333 (PMC10265085; doi:10.1002/advs.202207333)
Supplement: Supplementary file 1 — Supporting Information [file ADVS-10-2207333-s002.pdf]

ADVANCED SCIENCE
Open Access

Supporting Information

for *Adv. Sci.*, DOI 10.1002/advs.202207333

Highly Boosting Circularly Polarized Luminescence of Chiral Metal–Imidazolate Frameworks

Xue-Zhi Wang, Chuang-Wei Zhou, Ji Zheng, Zhao-Xia Lian, Meng-Ying Sun, Yong-Liang Huang, Dong Luo, Yan Yan Li and Xiao-Ping Zhou**

Supporting Information

Highly Boosting Circularly Polarized Luminescence of Chiral Metal-Imidazolate Frameworks

Xue-Zhi Wang, Chuang-Wei Zhou, Ji Zheng, Zhao-Xia Lian, Meng-Ying Sun, Yong-Liang Huang, Dong Luo, Yan Yan Li, and Xiao-Ping Zhou**

Table of Contents

Experimental Procedures **S3**

Chemicals *S3*

Synthesis of 2-isopropyl-4-methyl-1H-imidazole-5-carbaldehyde *S3*

Syntheses of MOFs ***P-Et, M-Et, P-Et(Cd) and M-Et(Cd)*** *S4*

Syntheses of MOFs ***P/M-Et⊃PhX, P/M-Et⊃o/p/m-PhF*** $_2$ ***and P/M-Et(Cd)⊃PhF*** *S5*

Characterizations *S6*

Computational detail *S7*

Figure S1-24 .... **S9**

Table S1-13 .... **S22**

References **S68**

Experimental Procedures

Chemicals

All chemicals and solvents were used without further purification. Zinc bromide ($ZnBr_2$, 99%, Bidepharm), cadmium bromide ($CdBr_2$, 99%, Bidepharm), 2-ethyl-5-methy1-1H-imidazole-5-carbaldehyde (97%, Bidepharm), (1R,2R)-(-)-1,2-diaminocyclohexane (99%, Energy Chemical), (1S,2S)-(+)-1,2-diaminocyclohexane (99%, Energy Chemical), fluorobenzene (PhF, 99%, Energy Chemical), chlorobenzene (PhCl, 99%, Energy Chemical), bromobenzene (PhBr, 99%, Energy Chemical), *o*-difluorobenzene (*o*-PhF_2, 99%, Energy Chemical), m-difluorobenzene (*m*-PhF_2, 99%, Energy Chemical), p-difluorobenzene (*p*-PhF_2, 99%, Energy Chemical), N,N-diethylformamide (DEF, 99.0%, TCI Development Co., Ltd.), methanol (99.5%,GHTECH), isobutyrimidamide hydrochloride (95%, Bidepharm), butane-2,3-dione (98%, ACMEC), manganese dioxide (93%, Bidepharm).

Synthesis of 2-isopropyl-4-methyl-1H-imidazole-5-carbaldehyde

2-isopropyl-4-methyl-1H-imidazole-5-carbaldehyde was carried out according to the literature procedures.[1]

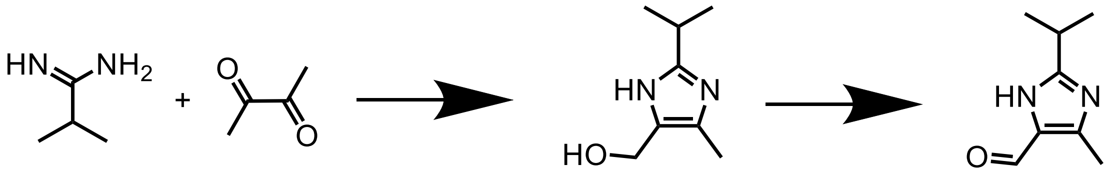

Scheme S1. Synthetic pathway for 2-isopropyl-4-methyl-1H-imidazole-5-carbaldehyde.

4-hydroxymethyl-5-methyl-2-isopropyl-3H-imidazole: Isobutyrimidamide hydrochloride (6.13 g, 50 mmol) was dissolved in the minimum amount of water (3 mL), than butane-2,3-dione (4.37 g, 51 mmol) was added. The mixture solution was stirred at room temperature for 3.25 h, and neutralised with aqueous sodium hydroxide (2 M). After neutralization, the mixture was incubated in air overnight. The white precipitate was yielded and collected by filtration. The solid product was washed with acetone. The solid was dissolved into aqueous hydrochloric acid (5 M, 80 mL), and the solution was heated under reflux for 3 h. After the solution was cooled to room temperature, it was neutralized with saturated aqueous sodium hydrogen carbonate. After the neutralization, the white precipitate was formed, which was collected by filtration and was washed with acetone to give 4-hydroxymethyl-5-methyl-2-isopropyl-3H-imidazole (4.26 g, 55.3%). ^{1}H-NMR (DMSO-d_6, 298 K, 400 MHz): δ_H 14.20 (1 H, br s), 5.49 (1 H, br s), 4.41 (2 H, s), 3.29-3.17 (1 H, m), 2.22 (3H, s), 1.32 (6H, d, *J* 7.0).

2-isopropyl-4-methyl-1H-imidazole-5-carbaldehyde: A solution of 4-hydroxymethyl-5-methyl-2-isopropyl-3H-imidazole (1.93 g, 13 mmol) in chloroform (75 mL) was heated under reflux with manganese dioxide (11.30 g, 130 mmol) for 1 h. The hot solution was then filtered through Celite. Then,

the solids were washed thoroughly with hot chloroform. The chloroform solution was collected, which was combined with the filtrate. The solution was evaporated to give the solid product (yield 68%). ^{1}H NMR (DMSO-d_6, 298 K, 400 MHz): δ_H 12.44 (1 H, br s), 9.68 (1 H, s), 3.0-2.86 (1 H, m), 2.39 (3H, s), 1.22 (6H, d, *J* 7.0). FT-IR spectrum (KBr, pellets, cm-1): 3191(m), 3095 (m), 2931(m), 2872 (m), 1659 (s), 1592 (m), 1541(s), 1421(m), 1374 (m), 1350 (m), 1307 (s), 1096 (m), 1012 (s), 847 (m), 651 (m).

Syntheses of MOFs P-Et and M-Et

P-Et: A mixture of $ZnBr_2$ (11.25 mg, 0.05 mmol), (1R,2R)-(-)-1,2-diaminocyclohexane (2.28 mg, 0.02 mmol), 2-ethyl-5-methy1-1H-imidazole-5-carbaldehyde (5.5 mg, 0.04 mmol), and DEF/methanol mixed solvent (3 mL, 4:1, v/v) was sealed in a Pyrex glass tube and heated in an oven at 120 °C for 72 hours and cooled to room temperature at a rate of 5 °C h^{-1}. The yellow rod-like crystals were obtained and washed with DEF and methanol, respectively. The yield is 57.8% (7.4 mg). IR spectrum (KBr, pellets, cm^{-1}): 2972(m), 1671(s), 1615(s), 1498(m), 1422(m), 1397(m), 1303(m), 889(m), 644(m), 508(m), 428(m). Elemental analysis (CHN): $C_{60.5}H_{101.5}N_{16.5}O_{4.5}Zn_4Br_4$ (corresponding to $[C_{38}H_{52}Br_4N_{12}Zn_4]\cdot 4.5DEF$), calculated (%): C 42.61, H 6.00, N 13.55; found (%): C 42.81, H 5.541, N 13.17.

M-Et: A mixture of $ZnBr_2$ (11.25 mg, 0.05 mmol), (1S,2S)-(+)-1,2-diaminocyclohexane (2.28 mg, 0.02 mmol), 2-ethyl-5-methy1-1H-imidazole-5-carbaldehyde (5.5 mg, 0.04 mmol) and DEF/methanol mixed solvent (3 mL, 4:1, v/v) was sealed in a Pyrex glass tube and heated in an oven at 120 ℃ for 72 hours and cooled to room temperature at a rate of 5 °C h^{-1}. The yellow rod-like crystals were obtained and washed with DEF and methanol, respectively. The yield is 54.4% (7.0 mg). IR spectrum (KBr, pellets, cm^{-1}): 2936(w), 1671(s), 1615(s), 1497(m), 1398(m), 1302(m), 1216(m), 1103(m), 1005(m), 889(m), 644(m), 508(m), 427(m). Elemental analysis (CHN): $C_{60.5}H_{101.5}N_{16.5}O_{4.5}Zn_4Br_4$ (corresponding to $[C_{38}H_{52}Br_4N_{12}Zn_4]\cdot 4.5DEF$), calculated (%):C 42.61, H 6.00, N 13.55; found (%): C 42.77, H 5.917, N 13.12.

Syntheses of MOFs P-Et(Cd) and M-Et(Cd)

P-Et(Cd): A mixture of $CdBr_2$ (13.6 mg, 0.05 mmol), (1R,2R)-(-)-1,2-diaminocyclohexane (2.28 mg, 0.02 mmol), 2-ethyl-5-methy1-1H-imidazole-5-carbaldehyde (5.5 mg, 0.04 mmol), and DEF/methanol mixed solvent (3 mL, 4:1, v/v) was sealed in a Pyrex glass tube and heated in an oven at 120 °C for 72 hours and cooled to room temperature at a rate of 5 °C h^{-1}. The yellow rod-like crystals were obtained and washed with DEF and methanol, respectively. The yield is 30% (4.5 mg). IR spectrum (KBr, pellets, cm^{-1}): 2933(m), 1665(s), 1617(s), 1394(m), 1300(m), 1261(m), 1212(m), 951(m), 642(m), 492(m). Elemental analysis (CHN): $C_{35}H_{61}N_9O_3Cd_2Br_2$ (corresponding to $[C_{20}H_{28}Br_2N_6Cd_2]\cdot 3DEF$), calculated (%): C 40.40, H 5.91, N 12.11; found (%): C 39.77, H 5.611, N 12.10.

M-Et(Cd): A mixture of $CdBr_2$ (13.6 mg, 0.05 mmol), (1S,2S)-(+)-1,2-diaminocyclohexane (2.28 mg, 0.02 mmol), 2-ethyl-5-methy1-1H-imidazole-5-carbaldehyde (5.5 mg, 0.04 mmol) and DEF/methanol mixed solvent (3 mL, 4:1, v/v) was sealed in a Pyrex glass tube and heated in an oven at 120 °C for 72 hours and cooled to room temperature at a rate of 5 °C h^{-1}. The yellow rod-like crystals were obtained and washed with DEF and methanol, respectively. The yield is 28% (4.1 mg). IR spectrum (KBr, pellets, cm^{-1}): 2935(w), 1645(s), 1612(s), 1497(m), 1395(m), 1310(m), 1300(m), 1261(m), 1003(m), 952(m), 642(m), 492(m). Elemental analysis (CHN): $C_{35}H_{61}N_9O_3Cd_2Br_2$ (corresponding to $[C_{20}H_{28}Br_2N_6Cd_2]$·3DEF), calculated (%): C 40.40, H 5.91, N 12.11; found (%): C 40.32, H 5.920, N 12.31.

Syntheses of MOFs P/M-Et⊃PhX, **P/M-Et⊃*o/m/p*-PhF**, **and P/M-Et(Cd)⊃PhF**

A mixture of $ZnBr_2$ or $CdBr_2$ (11.25 mg or 13.6 mg, 0.05 mmol), (1R,2R)-(-)-1,2-diaminocyclohexane or (1S,2S)-(+)-1,2-diaminocyclohexane (2.28 mg, 0.02 mmol), 2-ethyl-5-methy1-1H-imidazole-5-carbaldehyde (5.5 mg, 0.04 mmol), 0.25 mL of **PhX** or ***o/m/p*-PhF₂** (PhX = fluorobenzene, chlorobenzene, and bromobenzene, respectively; *o/m/p*-PhF_2 = *o/m/p*-difluorobenzene), and DEF/methanol mixed solvent (3 mL, 4:1, v/v) was sealed in a Pyrex glass tube and heated in an oven at 120 °C for 72 hours and cooled to room temperature at a rate of 5 °C h^{-1}. The yellow rod-like crystals were obtained and washed with DEF and methanol, respectively.

P-Et ⊃ PhF: Elemental analysis (CHN): $C_{59.2}H_{97}N_{16}O_4Zn_4Br_4F_{0.2}$ (corresponding to $[C_{38}H_{52}Br_4N_{12}Zn_4]$·4.0DEF·0.2PhF), calculated (%):C 42.48, H 5.84, N 13.39; found (%): C 42.48, H 5.854 N 12.87. **M-Et⊃PhF**: Elemental analysis (CHN): $C_{59.2}H_{99}N_{16}O_5Zn_4Br_4F_{0.2}$ (corresponding to $[C_{38}H_{52}Br_4N_{12}Zn_4]$·4.0DEF·0.2PhF·$H_2O$), calculated (%):C 42.03, H 5.90, N 13.25; found (%): C 42.17, H 5.951, N 13.24. **P-Et ⊃ PhCl**: Elemental analysis (CHN): $C_{59.2}H_{97}N_{16}O_4Zn_4Br_4Cl_{0.2}$ (corresponding to $[C_{38}H_{52}Br_4N_{12}Zn_4]$·4.0DEF·0.2PhCl), calculated (%):C 42.40, H 5.83, N 13.36; found (%): C 42.4, H 5.885, N 13.04. **M-Et ⊃ PhCl**: Elemental analysis (CHN): $C_{59.2}H_{97}N_{16}O_4Zn_4Br_4Cl_{0.2}$ (corresponding to $[C_{38}H_{52}Br_4N_{12}Zn_4]$·4.0DEF·0.2PhCl), calculated (%):C 42.40, H 5.83, N 13.36; found (%): C 42.47, H 5.896, N 12.89. **P-Et⊃PhBr**: Elemental analysis (CHN): $C_{54.8}H_{86.5}N_{15}O_3Zn_4Br_{4.3}$ (corresponding to $[C_{38}H_{52}Br_4N_{12}Zn_4]$·3.0DEF·0.3PhBr, calculated (%):C 41.12, H 5.45, N 13.13; found (%): C 41.17, H 5.742, N 12.91. **M-Et⊃PhBr**: Elemental analysis (CHN): $C_{55.4}H_{87}N_{15}O_3Zn_4Br_{4.4}$ (corresponding to $[C_{38}H_{52}Br_4N_{12}Zn_4]$·3.0DEF·0.4PhBr, calculated (%):C 41.17, H 5.43, N 13.00; found (%): C 41.25, H 5.541, N 13.07.

Characterizations

Fourier-transfer infrared (FT-IR) spectra were recorded on a Thermo Scientific FT-IR Nicolet iS10 spectrophotometer in the frequency range from 4000 – 400 cm^{-1} using a KBr module. Solid-state vibrational circular dichroism (VCD) spectra of the rotated solid sample were obtained on a ChiraIR Dual-PEM VCD spectrometer from BioTools. VCD spectra were collected on KBr pellets of the sample (1:40). The solid-state ultraviolet-visible (UV-vis) absorption spectra and electronic circular dichroism (ECD) were recorded on a Bio-Logic MOS-500 multifunctional circular dichroism spectrometer using pressed KBr pellets and measured at a scan speed of 30 $nm \cdot min^{-1}$ with a bandwidth of 1 nm. All spectroscopy studies were equipped with Biokine software V4.80. Each ECD spectrum was the average of at least three scans. Elemental analyses were determined by Elementar vario Micro Cube. NMR spectra were recorded on a Bruker 400 MHz spectrometer. Field Emission Scanning Electron Microscope (SEM) with energy dispersive spectrometer (EDS) was performed on a scanning electron microscope (COXEM EM-30 Plus, Korea). Powder X-ray diffraction (PXRD) experiments were performed on a Rigaku Ultima IV X-ray diffractometer (Cu Kα, λ = 1.5418 Å) in the step of 0.02° under the tube conditions 40 KV and 40 mA. The thermogravimetry analyses (TGA) were carried out on TGA Q50 V20.6 with a heating rate of 10 °C/min from 40 to 800 °C in N_2 atmosphere. Gas sorption isotherms were measured on a MicrotracBEL MAX II. The fluorescence lifetimes and absolute quantum yields were measured by employing Hamamatsu C11367 spectrometer under room temperature. Steady-state photoluminescence (PL) spectra for all solid-state samples were recorded on a Horiba FluoroMax-4 fluorometer. CPL spectra for all solid-state samples were recorded on a JASCO CPL-300 spectrophotometer in the solid state with scanning speed, E_x slit width, E_m slit width, and accumulations of 200 nm min^{-1}, 2000 μm, 2000 μm, and 2, respectively. The excitation wavelength was 390 nm and the DV values were about 0.5 V for all samples. In these processes, the solid-state samples (a small amount) were uniformly sandwiched by two quartz slides to test. Certainly, the instrument was carefully calibrated to eliminate the influence of other factors and the reproducibility of CPL spectrum for each sample was confirmed by measuring at least three times.

Single crystals data collections were performed on an Oxford Diffraction XtalAB [Rigaku, Japan, Cu Kα, λ = 1.54178 Å] equipped with a monochromator and CCD plate detector (CrysAlisPro CCD, Oxford Diffraction Ltd) at 100 K. Data collection and reduction were performed by the program CrysAlisPro. Structures were solved by direct methods using *SHELXT* in *OLEX2* program package[2] and refined by full-matrix least-squares on F^2 with anisotropic displacement parameters for all non-hydrogen atoms using the *SHELXL* program.[3] The hydrogen atoms were located from different maps and refined with isotropic temperature factors. The treatment for the disordered guest molecules in the cavities of all complexes involved the use of the *SQUEEZE* program of *PLATON*.[4] Detailed

information about the X-ray crystal data, intensity collection procedure and refinement results for all compounds is summarized in Table S1. CCDC Nos. 2194055, 2194056, 2243850 and 2243851.

Computational detail

For exploring the host-guest interactions in these host-guest complexes, the model of **P-Et** with guest fluorobenzene molecules was thus selected as an example. Because a single crystal of **P-Et** occupied by fluorobenzene guests failed to be obtained, the CASTEP module of Materials Studio software has been applied to yield the desired model.[5] During the calculation, the framework from the crystal structure of **P-Et** was frozen, while the configurations and positions of the included fluorobenzene molecules were optimized. The periodic PBE functional and OTFG ultra-soft pseudopotential were adopted in this part of the calculation.[6, 7]

The above computation returns a framework model in which only the small pockets were occupied by fluorobenzene molecules, and only one fluorobenzene exists in each small cavity. Therefore, a simplified host-guest complex model (denoted as **P-Et⊃PhF**), reproducing a small cavity with an included fluorobenzene, was extracted from the optimized periodic host-guest complex model. Similar to the situation in the periodic model, the model of **P-Et⊃PhF** contains one fluorobenzene molecule, eight Zn(II) cations, and eight Br^- anions. However, some of the organic ligands were cut off and saturated. First of all, for the ligand bridging two Zn(II) ions in this octanuclear Zn(II) model by forming two ZnN_2C_2 chelating five-membered ring, it is not cut off, and thus the ligand is consistent with the original organic ligand $(L1)^{2-}$. Secondly, for the organic ligand bridging two Zn(II) ions in this octanuclear Zn(II) model by two N atoms from an imidazolyl group and forming a ZnN_2C_2 chelating five-membered ring with one of the two Zn(II) ions in the help of the N atom of the C=N group, only half of the original ligand remained and the cyclohexyl group was simplified to the methyl group. In this case, the ligand is denoted as $(L2)^-$. Thirdly, for the organic ligand only coordinated with one Zn(II) ion in this octanuclear Zn(II) model by the N atom from an imidazolyl group, only the coordinated imidazolyl group remained with the uncoordinated N atom protonated to keep the whole model neutral, while the other part of this ligand was altered to a methyl group. In this case, the ligand is denoted as HL3. After the above treatment, the formula of **P-Et⊃PhF** is $Zn_8Br_8(L1)_2(L2)_4(HL3)_2$⊃Ph-F. The octanuclear Zn(II) model of **P-Et⊃*o*-PhF$_2$** was gotten by the same method.

Based on the models of **P-Et⊃PhF** with and without the fluorobenzene molecule, further quantum chemical calculations in the framework of density functional theory (DFT) were performed to shed light on the nature of ground-state host-guest interactions and the effects of guests on the spectral properties. In the geometrical optimization of the ground state (S_0), the eight Zn atoms were frozen while the other atoms were relaxed. The following level of theory was selected: (1) Functional: the hybrid Perdew, Burke, and Ernzerhof functional (PBE0) with D3(BJ) dispersion correlation;[8-11] (2) Basis sets: the

LANL2DZ effective core potential (ECP) was applied for Zn and the 6-31G(d,p) basis set was applied for the other atoms.[12, 13] The above level was also accepted to obtain the optimized geometry of the lowest singlet-excited state (S_1) by time-dependent DFT (TDDFT) method.

Furthermore, the independent gradient model based on Hirshfeld partition (IGMH) was used to visualize the ground-state host-guest interactions in **P-Et⊃PhF**.[14] In this part, the calculation was carried out by using M06-2X functional[15] and Def-TZVP basis set,[16] and the output files were furtherly treated by Multiwfn program.[17] Then, the isosurface map of IGMH (isovalue = 0.05 a.u.) was drawn using VMD 1.9 program.[18] The above level was also adopted in the TDDFT calculation to obtain the luminescence dissymmetry factor (g_{lum}). The three parameters of transition dipole moments and their angle of vectors relative to g_{lum} were calculated based on the first excited states S1. With the $\boldsymbol{\mu}$, $\boldsymbol{m}$ and $\theta_{\mu,\text{m}}$ components in hands, the dissymmetry factor, $g_{\text{lum}} = 4(|\boldsymbol{\mu}|\cdot|\boldsymbol{m}|\cdot\cos\theta_{\mu,\text{m}})/(|\boldsymbol{\mu}|^2 + |\boldsymbol{m}|^2)$, can be obtained easily. The result files (fchk and log) were furtherly treated by Multiwfn 3.8 software for extracting the information of electronic transitions and generating the cubic files (cub) of electron density difference (EDD). The isovalue of EDD maps was 0.0005 a.u. The nature of excited states was clarified by EDD maps. All the above computations using the octanuclear Zn(II) models were performed by Gaussian 09E package.[19]

To clarify the energy dominating the ground-state host-guest interactions (e.g., orbital attraction, electrostatic interaction, dispersion interaction) in **P-Et⊃PhF**, energy decomposition analysis (EDA) calculations[20-22] were performed using AMS2018 program[23, 24] with the treatment of ZORA Hamiltonian[25-27] for scalar relativistic effects. In this part, the dispersion-corrected BLYP-D3(BJ) functional[28] was utilized, along with the triple-zeta polarization (TZ2P) basis set[29] without frozen core approximation.

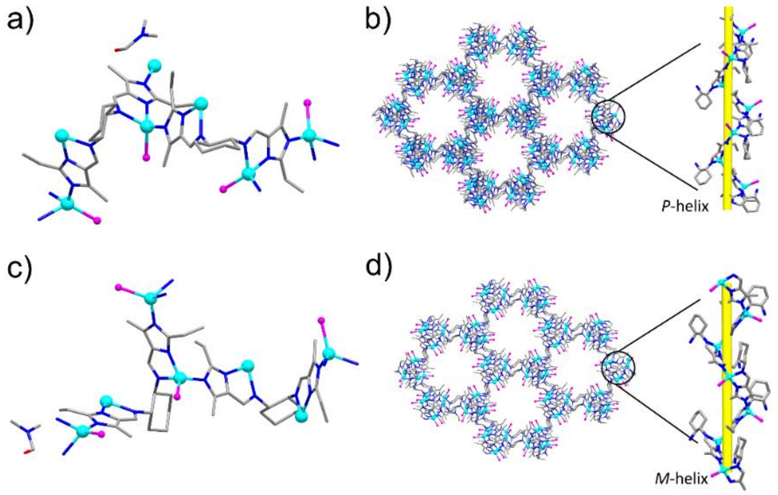

Figure S1. Crystal structure of **P-Et**: (a) asymmetric unit and (b) three-dimensional network structure along the *c*-axis and the *P*-helix. Crystal structure of **M-Et**: (c) asymmetric unit and (d) three-dimensional network structure along the *c*-axis and the *M*-helix. Color scheme: Zn, cyan; Br, pink; N, blue; C, gray; O, red; H, omitted.

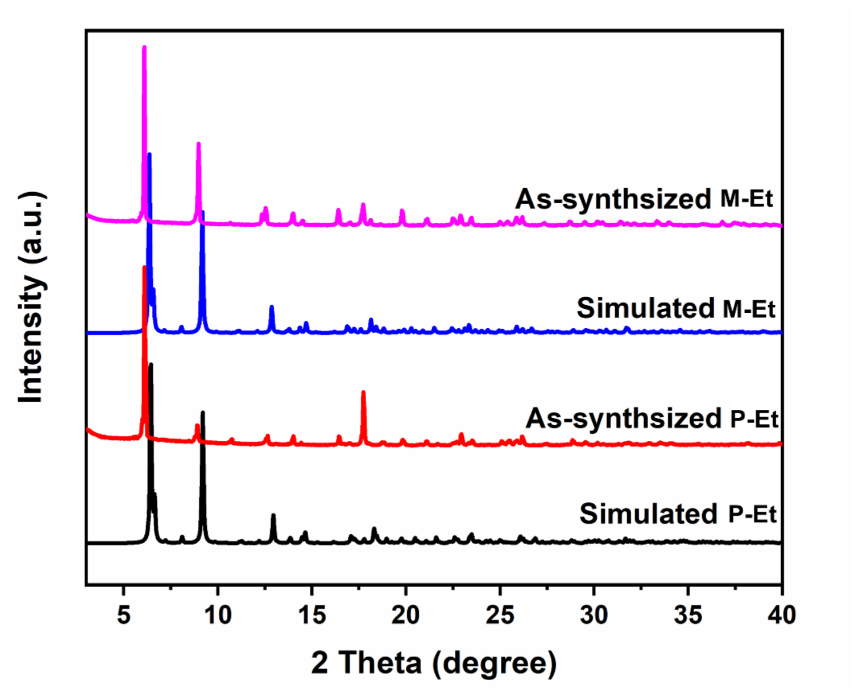

Figure S2. PXRD patterns of as-synthesized samples and the simulated PXRD patterns from the single crystals X-ray structures of MOFs **P/M-Et**.

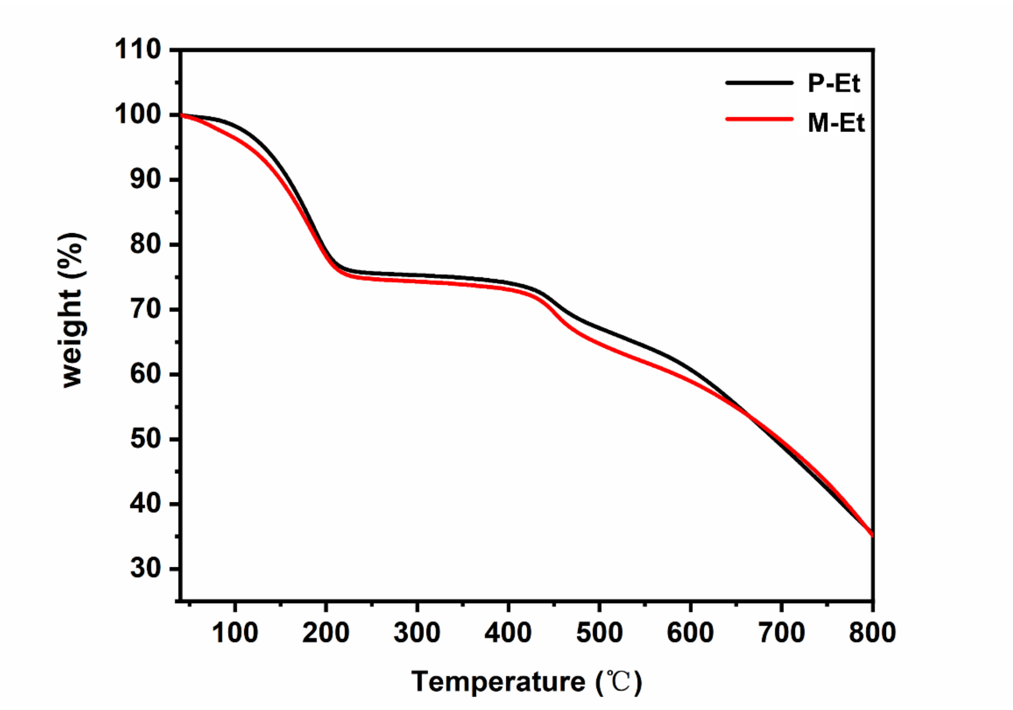

Figure S3. TGA plots of **P-Et** and **M-Et**.

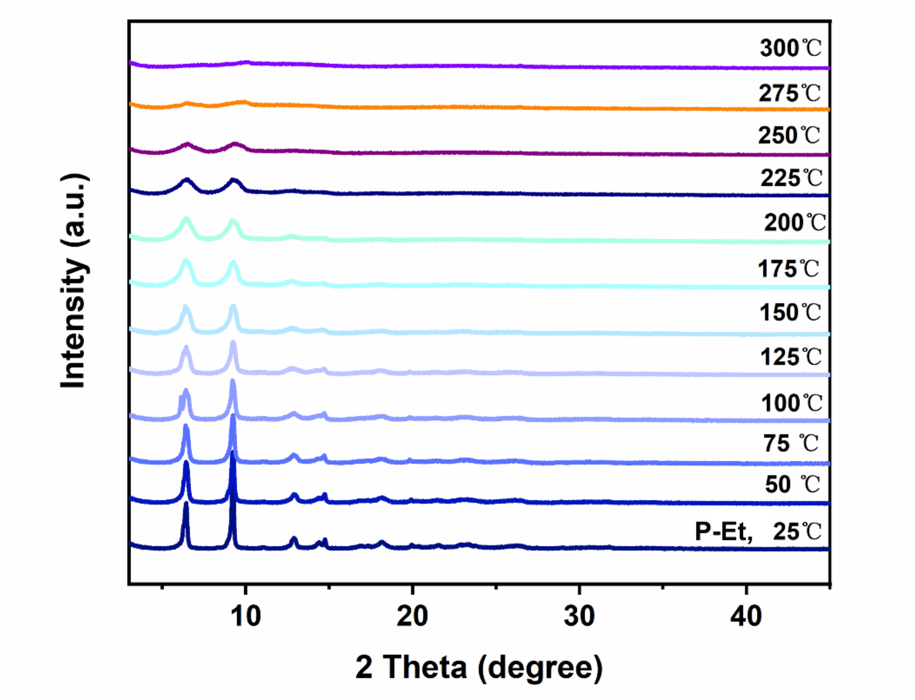

Figure S4. Variable-temperature PXRD patterns of **P-Et**.

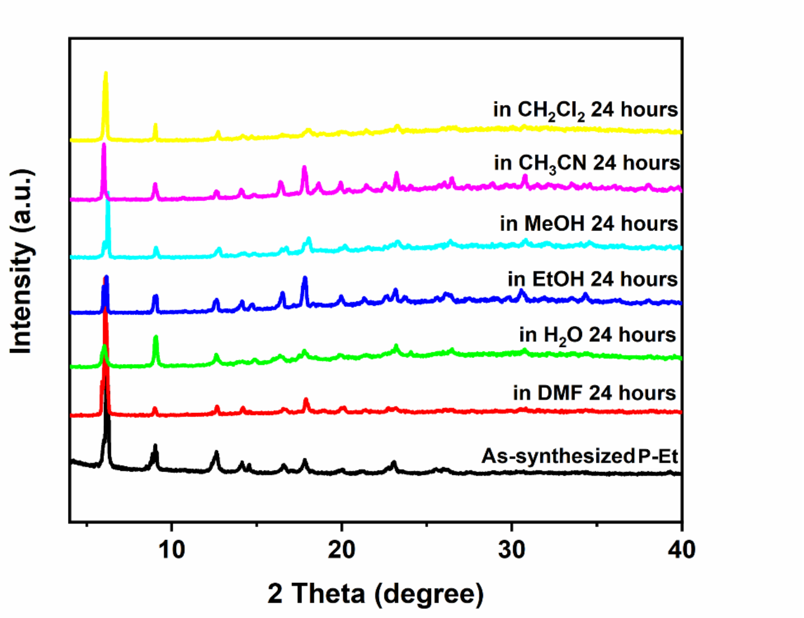

Figure S5. PXRD patterns of **P-Et**: as-synthesized samples and samples after treatment with organic solvents (DMF, ethanol, methanol, acetonitrile, and dichloromethane) and water.

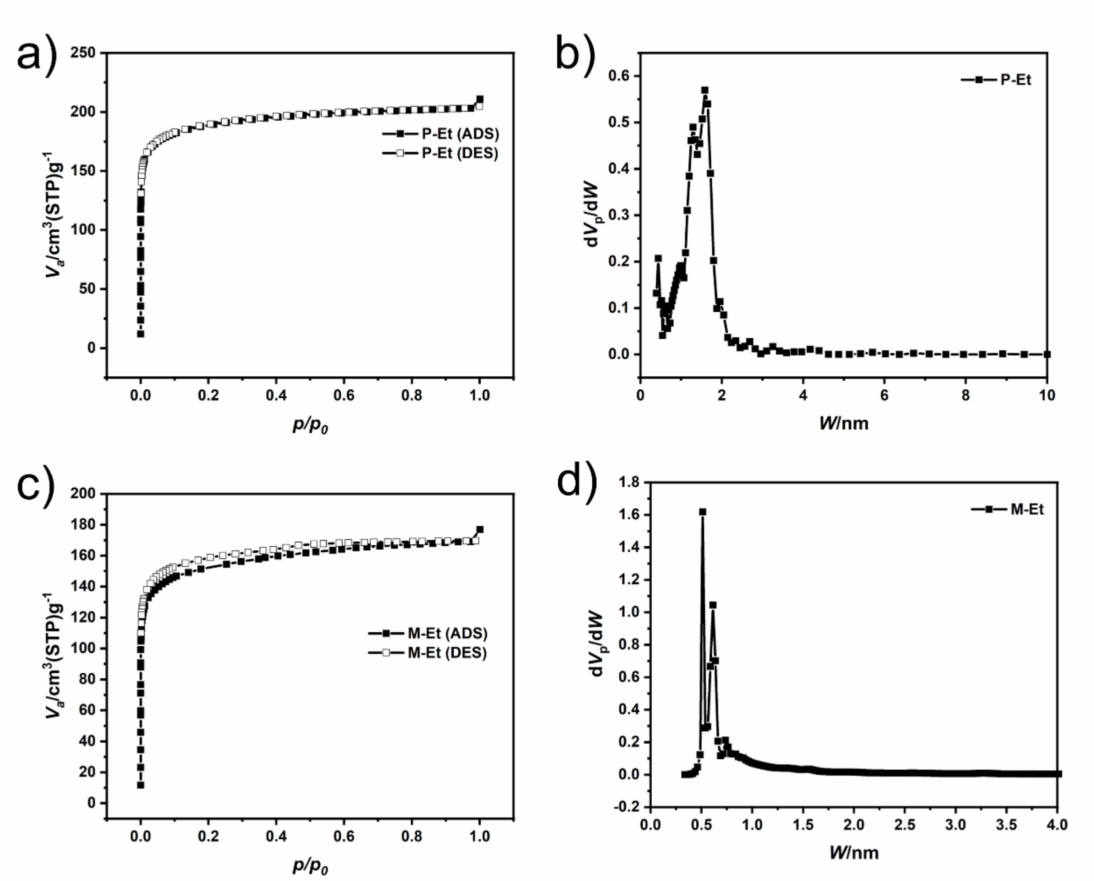

Figure S6. N_2 sorption isotherms of (a) **P-Et** and (c) **M-Et** at 77 K. The pore size distributions of (b) **P-Et** and (d) **M-Et**.

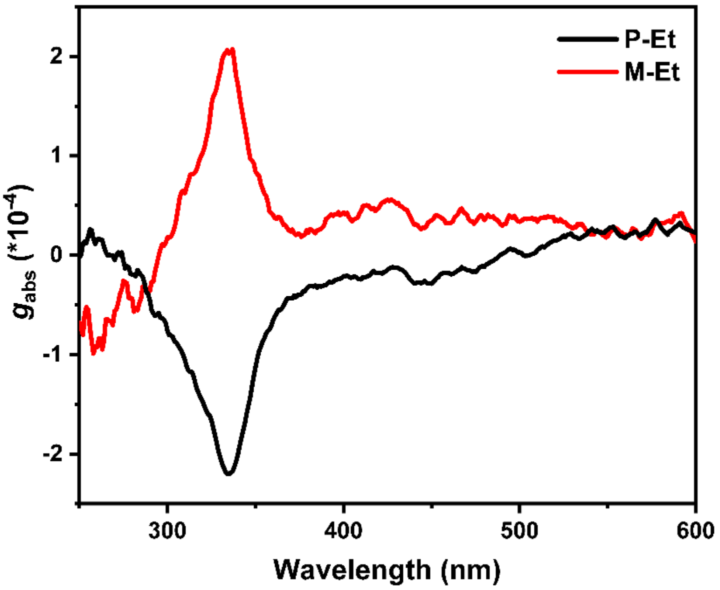

Figure S7. The g_{abs} spectra of **P-Et** (black line) and **M-Et** (red line).

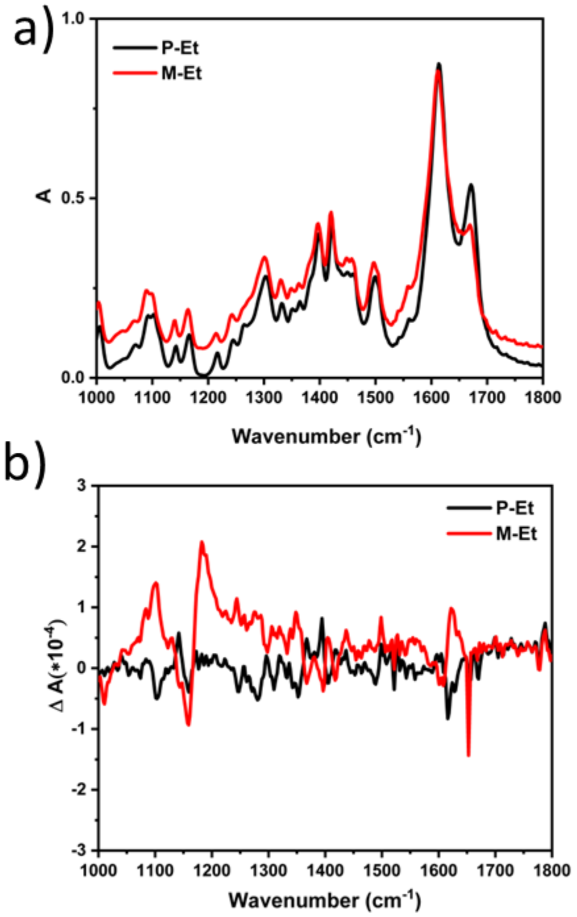

Figure S8. FT-IR (a) and solid-state VCD (b) spectra of **P-Et** (black line) and **M-Et** (red line) .

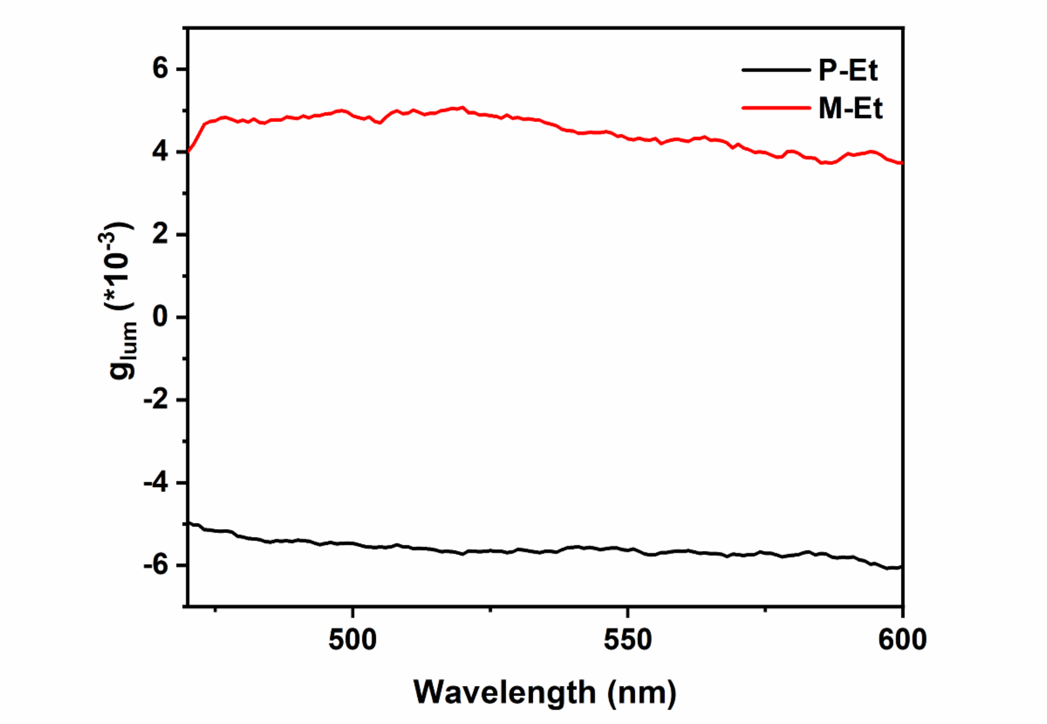

Figure S9. The g_{lum} plots of **P-Et** (black line) and **M-Et** (red line).

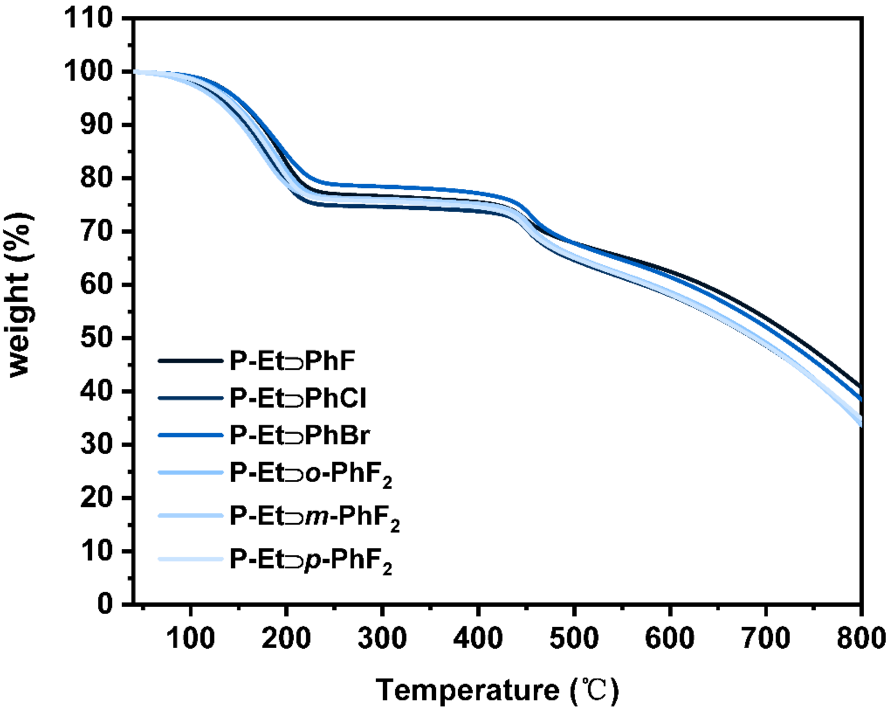

Figure S10. TGA plots of **P-Et⊃PhX** and **P-Et⊃*o/m/p*−PhF$_2$**.

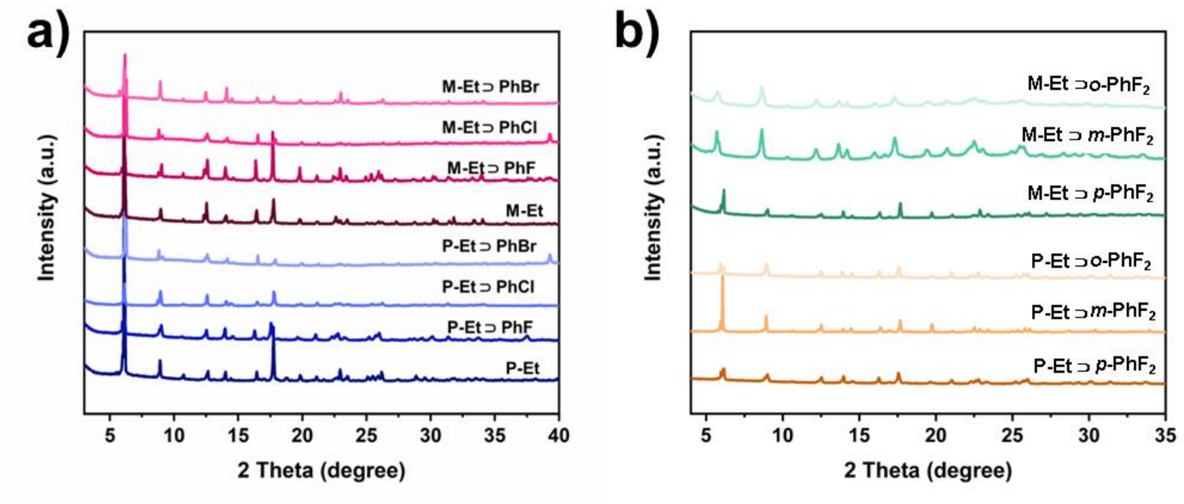

Figure S11. PXRD patterns for as-synthesized samples of (a) **P/M-Et**, **P/M-Et⊃PhF**, **P/M-Et⊃PhCl**, **P/M-Et⊃PhBr**, (b) **P/M-Et⊃*o*-PhF$_2$**, **P/M-Et⊃*m*-PhF$_2$**, and **P/M-Et⊃*p*-PhF$_2$**.

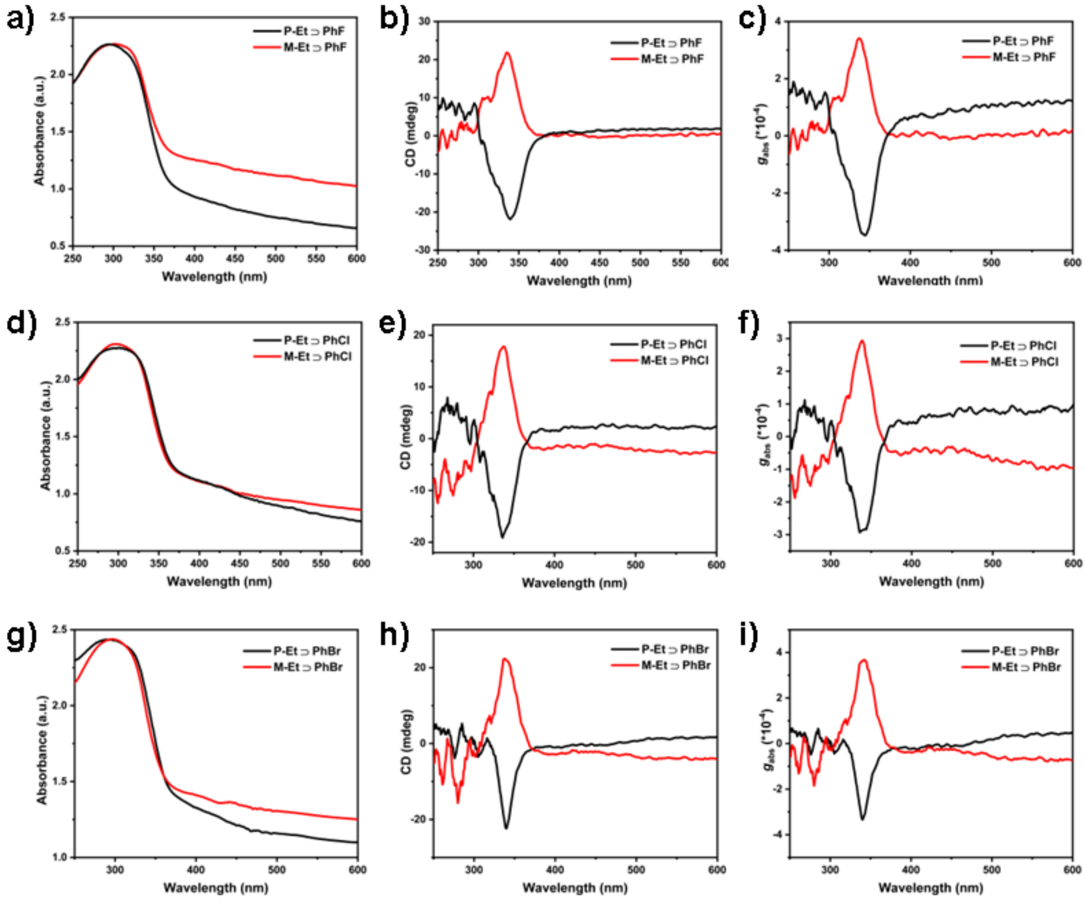

Figure S12. (a,d,g) UV−vis absorption, (b,e,h) ECD and (c,f,i) g_{abs} spectra of **P/M-Et⊃PhF**, **P/M-Et⊃PhCl**, and **P/M-Et⊃PhBr**.

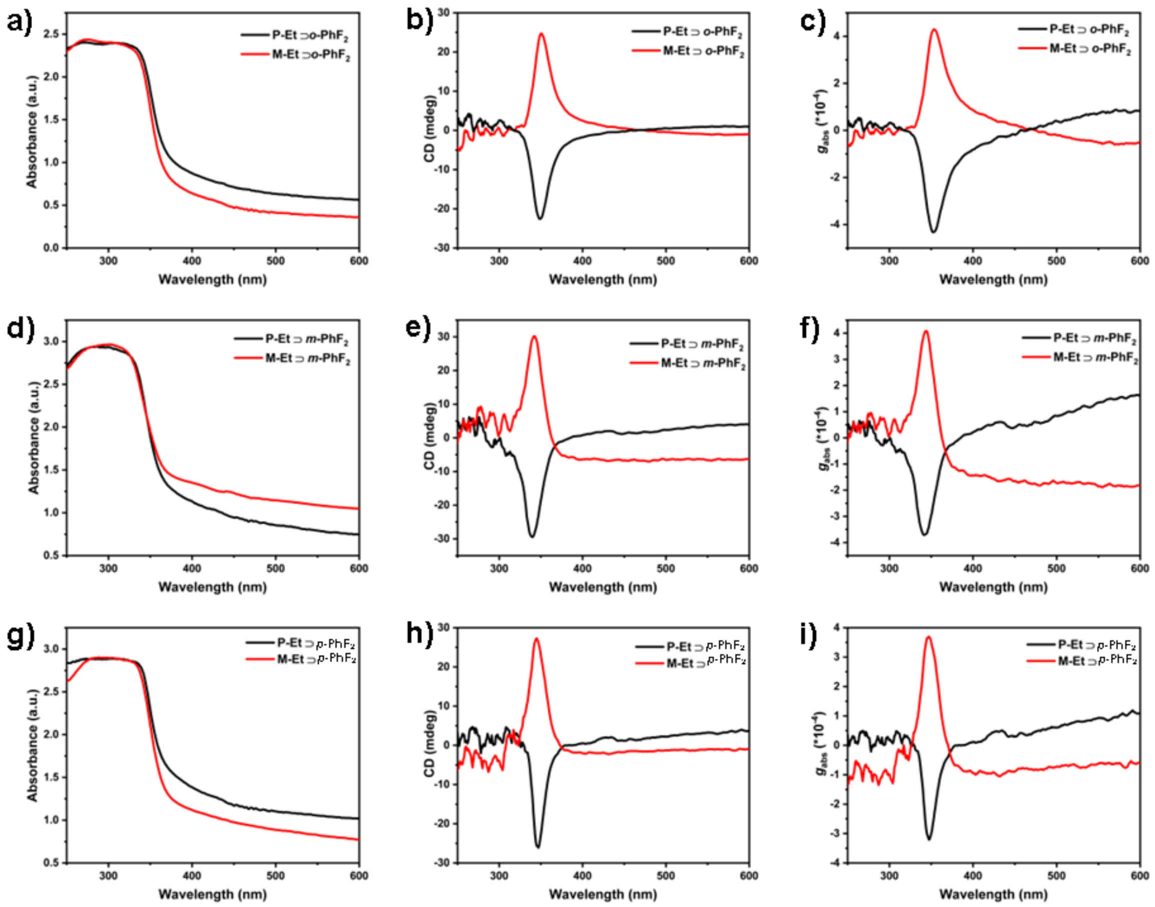

Figure S13. (a,d,g) UV−vis absorption, (b,e,h) ECD and (c,f,i) g_{abs} spectra of **P/M-Et⊃*o*-PhF$_2$**, **P/M-Et⊃*m*-PhF$_2$**, and **P/M-Et⊃*p*-PhF$_2$**.

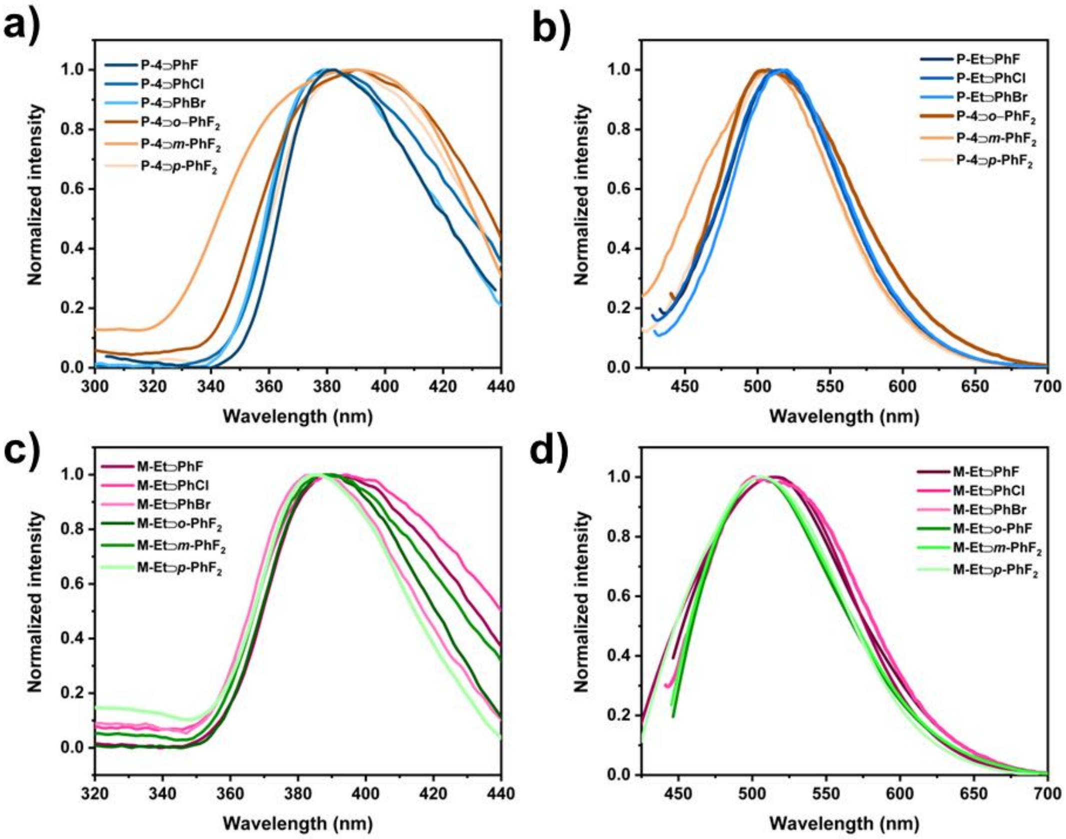

Figure S14. Normalized (a, c) excitation and (b,d) emission spectra of **P/M-Et⊃PhX**, **P/M-Et⊃*o*-PhF$_2$**, **P/M-Et⊃*m*-PhF$_2$**, and **P/M-Et⊃*p*-PhF$_2$**, respectively.

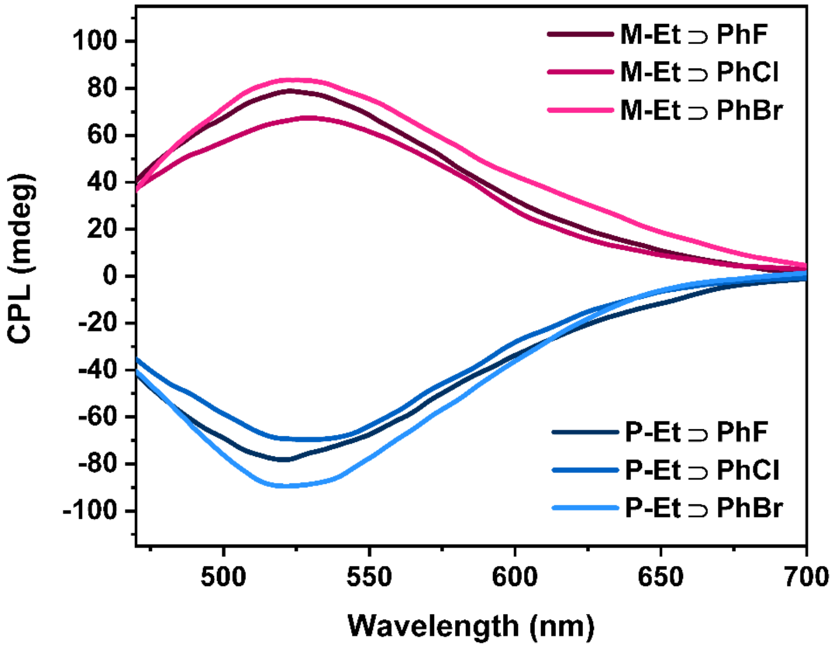

Figure S15. CPL spectra of **P/M-Et**, **P/M-Et⊃PhF**, **P/M-Et⊃PhCl**, and **P/M-Et⊃PhBr**.

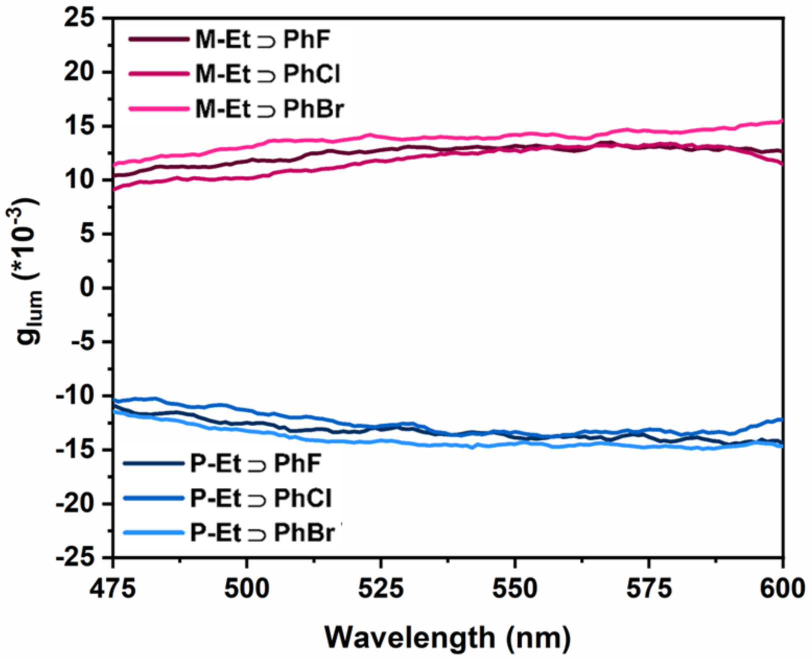

Figure S16. g_{lum} spectra of **P/M-Et**, **P/M-Et⊃PhF**, **P/M-Et⊃PhCl**, and **P/M-Et⊃PhBr**.

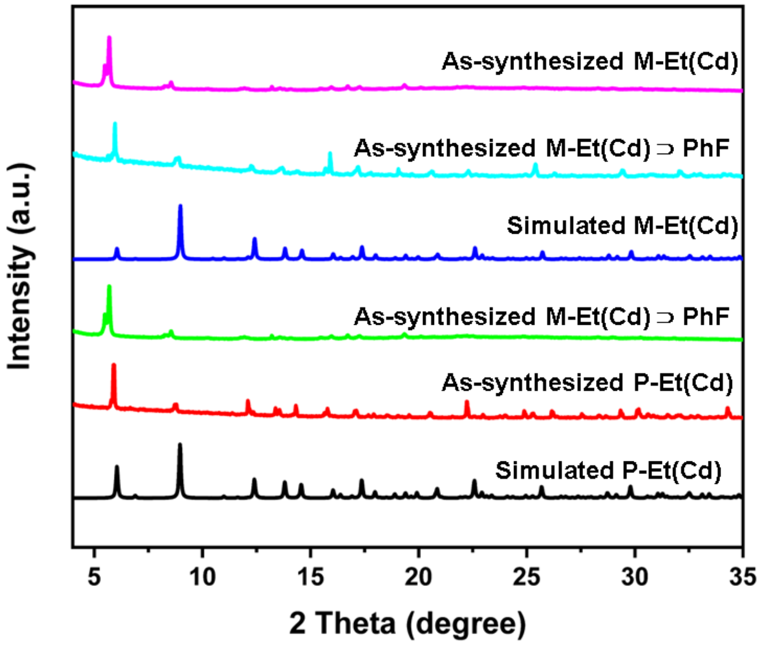

Figure S17. PXRD patterns for simulated and as-synthesized samples of **P/M-Et(Cd)** and **P/M-Et(Cd) ⊃PhF**.

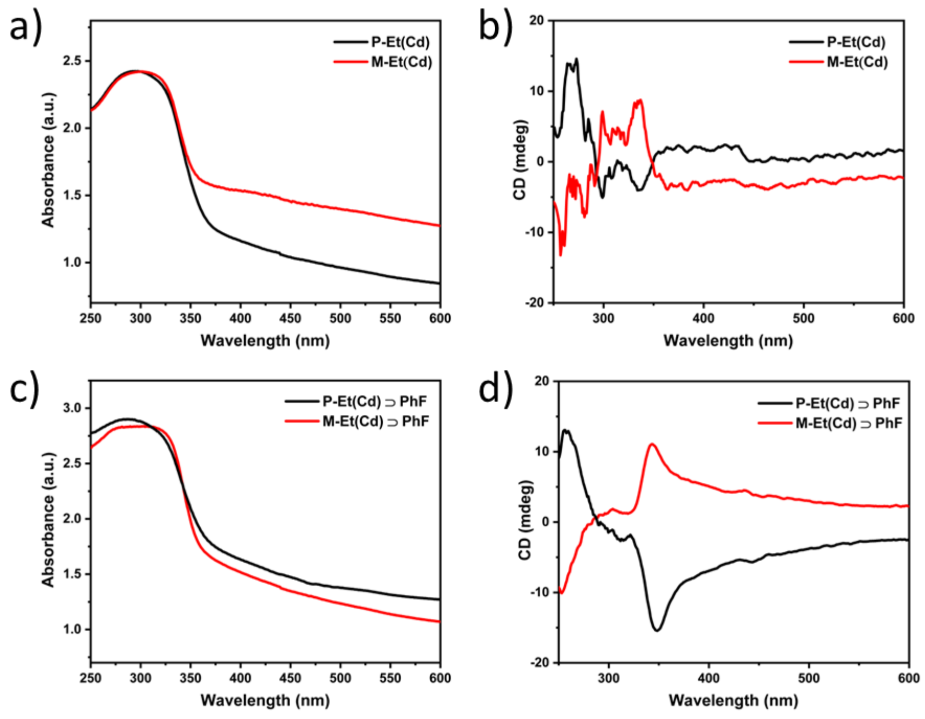

Figure S18. (a,c) UV−vis absorption and (b,d) ECD spectra of **P/M-Et(Cd)** and **P/M-Et(Cd)⊃PhF**.

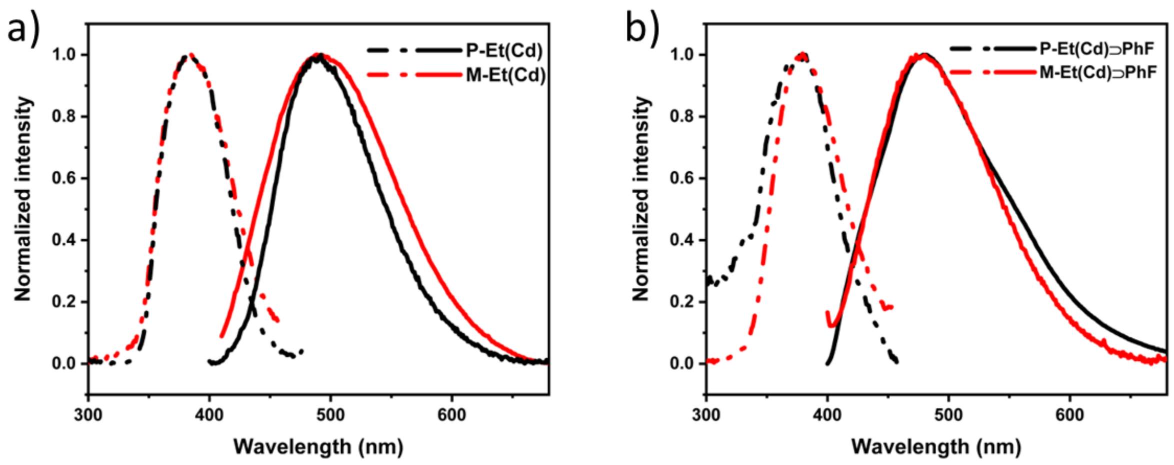

Figure S19. Normalized excitation and emission spectra of (a) **P/M-Et(Cd)** and (b) **P/M-Et(Cd)⊃ PhF**, respectively.

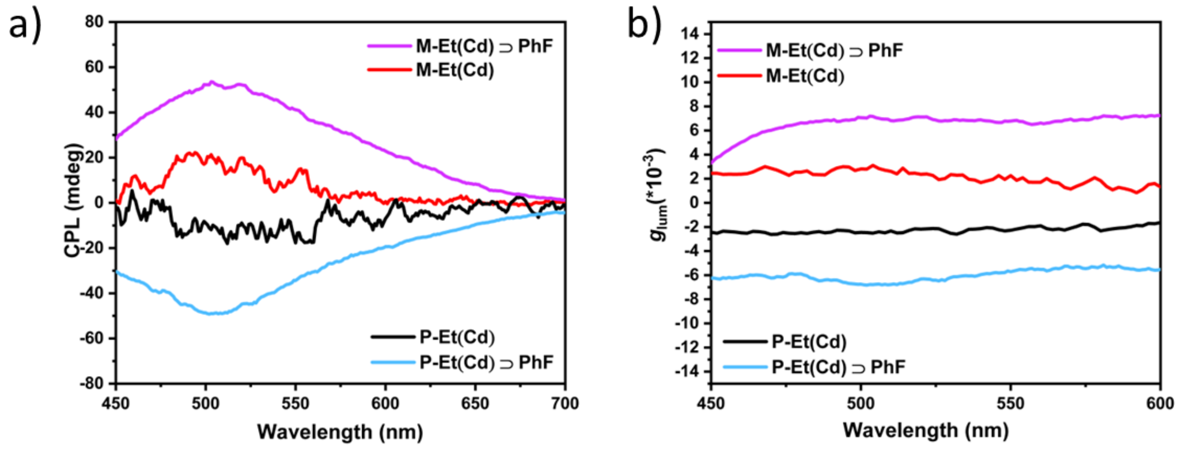

Figure S20. (a) CPL and g_{lum} spectra of **P/M-Et(Cd)** and **P/M-Et(Cd)⊃PhF**.

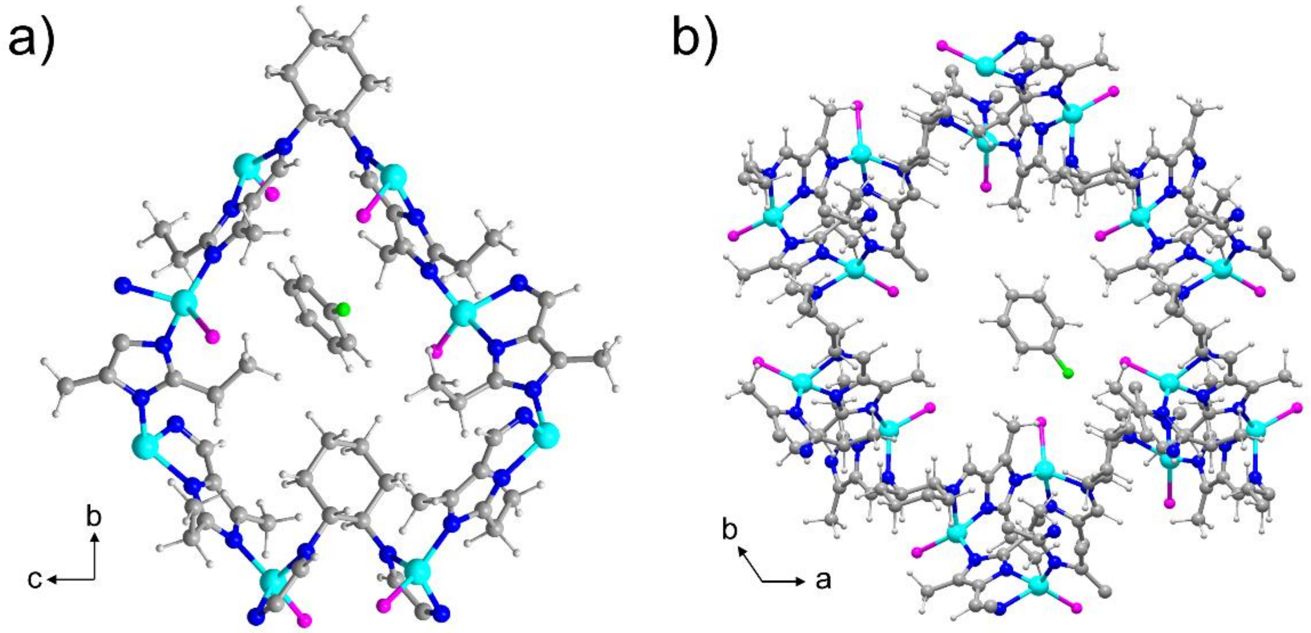

Figure S21. Structures of fluorobenzene placed in the channels of **P-Et** (a) *a*/*b*- and (b) *c*-axis. Color scheme: Zn, cyan; Br, pink; N, blue; F, green; C, gray; H, light gray.

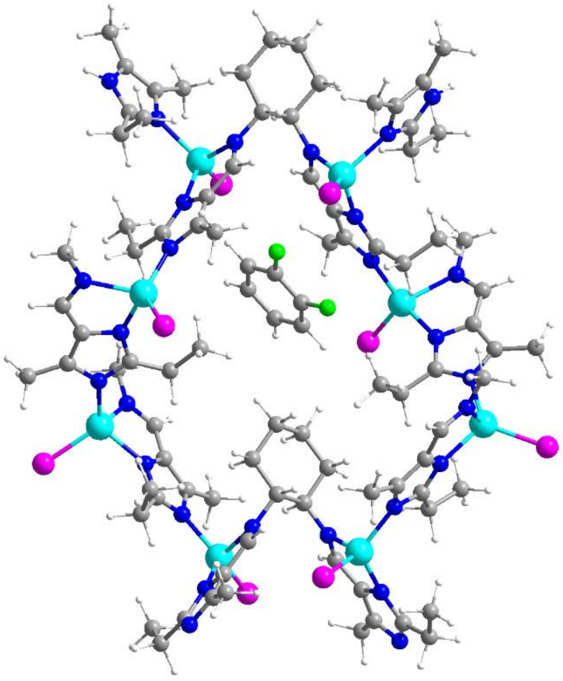

Figure S22. The optimized excited-state structure of **P-Et⊃*o*-PhF$_2$**. Color scheme: Zn, cyan; Br, pink; N, blue; F, green; C, gray; H, light gray.

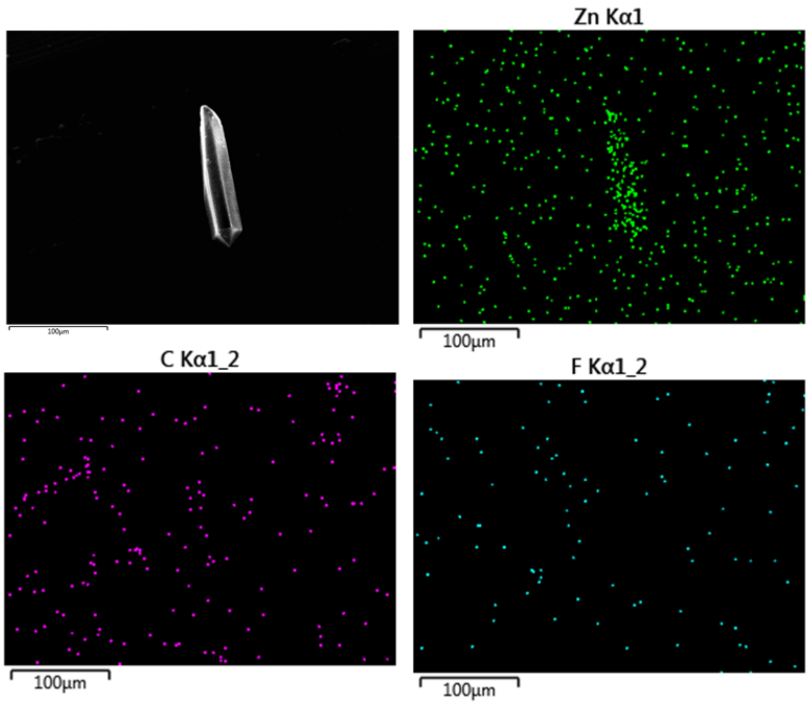

Figure S23. SEM image and the corresponding elemental mapping of **P-Et⊃PhF**.

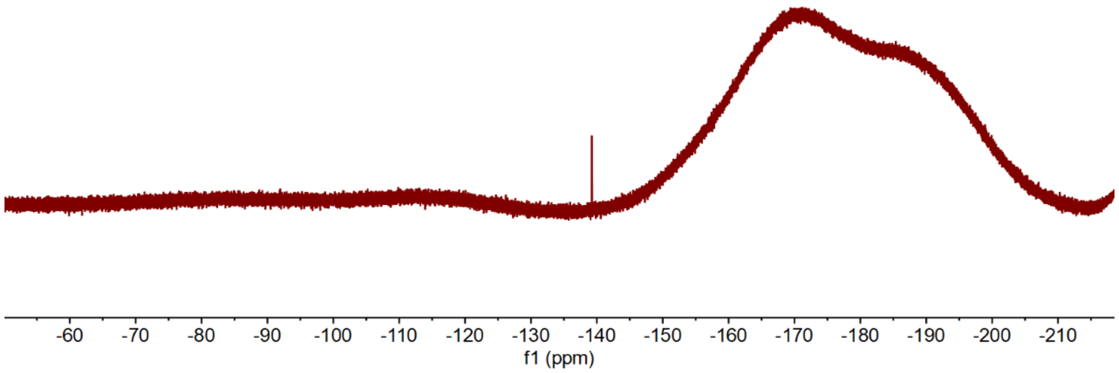

Figure S24. ^{19}F NMR spectrum of **P-Et⊃PhF** after digestion.

Table S1. Crystallographic data of **P-Et** and **M-Et**.

Parameter	**P-Et**	**M-Et**
Chemical formula	$C_{43}H_{63}Br_4N_{13}OZn_4$	$C_{43}H_{63}Br_4N_{13}OZn_4$
Formula weight	1359.18	1359.18
Temperature (K)	100.01(13)	100.01(12)
Crystal system	hexagonal	hexagonal
Space group	$P6_1$	$P6_5$
a (Å)	15.85680(10)	16.03830(10)
b (Å)	15.85680(10)	16.03830(10)
c (Å)	53.8336(4)	53.4954(4)
α (deg)	90	90
β (deg)	90	90
γ (deg)	120	120
Volume (Å^3)	11722.36(17)	11916.91(17)
Z	6	6
D_{calcd} (g cm^{-3})	1.155	1.136
μ (mm^{-1})	4.034	3.968
Reflections collected	92809	125718
Unique reflections	16188	15714
R_{int}	0.0392	0.0458
Goodness-of-fit on F^2	1.077	1.062
R_1[a] [I > 2σ(I)]	0.0471	0.0485
wR_2[b] [I > 2σ(I)]	0.136	0.1346
R_1[a] [all refl.]	0.0487	0.0498
wR_2[b] [all refl.]	0.1378	0.1364
Flack parameter	-0.002(6)	0.06(2)
CCDC number	2194055	2194056

Table S2. Crystallographic data of **P-Et(Cd)** and **M-Et(Cd)**.

Parameter	**P-Et(Cd)**	**M-Et(Cd)**
Chemical formula	$C_{35}H_{61}Br_2Cd_2N_9O_3$	$C_{30}H_{50}Br_2Cd_2N_8O_2$
Formula weight	1040.54	939.40
Temperature (K)	100.00(10)	99.99(10)
Crystal system	hexagonal	hexagonal
Space group	$P6_5$	$P6_1$
a (Å)	16.8644(2)	16.85960(10)
b (Å)	16.8644(2)	16.85960(10)
c (Å)	26.7222(3)	26.6465(2)
α (deg)	90	90
β (deg)	90	90
γ (deg)	120	120
Volume (Å^3)	6581.80(17)	6559.42(9)
Z	6	6
D_{calcd} (g cm^{-3})	1.575	1.427
μ (mm^{-1})	10.233	10.183
Reflections collected	75332	53286
Unique reflections	8870	9097
R_{int}	0.0657	0.0503
Goodness-of-fit on F^2	1.145	1.053
R_1[a] [I > 2σ(I)]	0.0476	0.0631
wR_2[b] [I > 2σ(I)]	0.1386	0.1656
R_1[a] [all refl.]	0.0481	0.0633
wR_2[b] [all refl.]	0.1393	0.1658
Flack parameter	-0.018(6)	0.039(13)
CCDC number	2243850	2243851

Table S3. Photophysical date of **P/M-Et**, **P/M-Et⊃PhX**, **P/M-Et⊃*o/m/p*-PhF$_2$** in solid state at 298 K.

compound	λ_{abs} (nm)	g_{abs} (10^{-4})	λ_{em} (nm)	τ_{av} (ns)	Φ_{PL}(%)	ξ_{abs}	g_{lum} (10^{-3})	|FM| (10^{-3})	B_{CPL} (10^{-5})
**P-Et**	295	-2.1	518	1.78	27.2	0.229	-5.7	1.6	17.8
**P-Et⊃PhF**	298	-3.4	514	1.85	47.3	0.357	-13	6.1	109.8
**P-Et⊃PhCl**	300	-2.9	515	1.87	40.3	0.344	-13	5.2	90.1
**P-Et⊃PhBr**	298	-3.3	517	2.04	40.6	0.382	-14	5.7	108.6
**P-Et⊃*o*-PhF$_2$**	310	-4.3	512	2.16	43.8	0.331	-8.3	3.6	60.2
**P-Et⊃*m*-PhF$_2$**	310	-3.7	507	2.18	36.3	0.34	-8.4	3.0	51.9
**P-Et⊃*p*-PhF$_2$**	313	-3.2	513	2.19	43.3	0.371	-8.3	3.6	66.8
**M-Et**	295	2.2	518	1.74	28.0	0.235	5.1	1.4	16.8
**M-Et⊃PhF**	300	3.4	515	1.79	46.6	0.352	13	6.1	106.6
**M-Et⊃PhCl**	298	2.9	515	1.79	39.5	0.398	12	4.7	94.3
**M-Et⊃PhBr**	298	3.6	517	1.95	40.2	0.334	15	6.0	100.7
**M-Et⊃*o*-PhF$_2$**	310	4.3	503	2.15	42.7	0.332	8.4	3.6	59.5
**M-Et⊃*m*-PhF$_2$**	310	4.1	500	2.14	37.9	0.349	8.6	3.3	56.8
**M-Et⊃*p*-PhF$_2$**	313	3.7	513	2.23	42.4	0.351	8.7	3.7	64.6

Table S4. Photophysical date of **P/M-Et(Cd)** and **P/M-Et(Cd)⊃PhF** in solid state at 298 K.

compound	λ_{abs} (nm)	g_{abs} (10^{-4})	λ_{em} (nm)	τ_{av} (ns)	Φ_{PL}(%)	ξ_{abs}	g_{lum} (10^{-3})	|FM| (10^{-3})	B_{CPL} (10^{-5})
**P-Et(Cd)**	300	-0.8	492	1.08	16.4	0.203	-2.4	0.4	4.0
**P-Et(Cd)⊃PhF**	300	-2.1	480	1.2	27.7	0.235	-6.8	1.8	14.4
**M-Et(Cd)**	300	1.2	492	1.04	14.0	0.207	3.1	0.4	4.5
**M-Et(Cd)⊃PhF**	300	1.8	480	1.0	28.4	0.194	7.2	2.0	19.8

Table S5. The reported $|g_{lum}|$, Φ_{PL}, and |FM| values for CPL-active non-lanthanide MOFs.

CPL-active MOFs	$\|g_{lum}\|$ (10^{-3})	Φ_{PL} (%)	|FM| (10^{-3})	Ref.
**Zn-CMOF-L/D**	0.8	7.2	0.06	[30]
**Cd-CMOF-L/D**	10.0	45.5	4.55	
**Cd-TCPA**	2.7	43.2	1.17	[31]
**L/D-MOF**	12.0	43.0	5.16	[32]
**L/D-ZIF⊃S420**	0.9	59.0	0.53	[33]
**γCD-MOF⊃Ru-BPen**	15	16.92	2.54	[34]
**pCp-1**	7.2	87.0	6.26	[35]
**pCp-2**	8.3	61.9	5.14	
**L/D-CMOF**	1.5	6.0	0.09	[36]
**L/D-CMOF⊃CBS**	11.5	66	7.26	
**P/M-1 (P/M-Me)**	2.3	6.4	0.15	[37]
**P/M-2**	1.0	4.9	0.05	

The results were derived in solid state at 298 K.

Table S6. Interactions between **P-Et** and **PhF**.

**P-Et···PhF**	Distances (Å)
C-Ha···Br1	3.130
C-Hb···Br2	2.996
C-H1···F	2.531
C-H2···F	2.483
C-Hc···C	2.798
benzene ring···imidazole ring	3.522

The interaction distances are directly measured by Diamond software.

Table S7. EDA of the interaction energy between **PhF** and 8-membered ring window of **P-Et**.

Energy	kcal mol^{-1}
Pauli Repulsion	30.82
Electrostatic Interaction	-13.21
Orbital Interaction	-6.94
Dispersion Interaction	-33.69
Total Interaction Energy	-23.02

Table S8. Transition dipole moments and g_{lum} values of **P-Et**, **P-Et⊃PhF** and **P-Et⊃*o*−PhF$_2$**.

compound	$\lvert\boldsymbol{\mu}\rvert$[a] ($10^{-20}$ esu·cm)	$\lvert\boldsymbol{m}\rvert$[b] ($10^{-23}$ erg·G^{-1})	cos $\theta_{\mu,\mathrm{m}}$[c]	$g_{\mathrm{lum, cal}}$	$g_{\mathrm{lum, exp}}$
**P-Et**	1.2310487	3.188232	-0.230	-2.38×10^{-3}	-5.70×10^{-3}
**P-Et⊃PhF**	0.2063506	3.854043	-0.372	-2.78×10^{-2}	-1.28×10^{-2}
**P-Et⊃*o*−PhF$_2$**	2.5531738	5.03934	-0.527	-4.16×10^{-3}	-8.4×10^{-3}

TDDFT calculation at the M06-2X/def-TZVP level using the excited-state structures.

[a] The electric transition moment. [b] The magnetic transition moment. [c] $\theta_{\mu,\mathrm{m}}$ is the angle of vectors between $\boldsymbol{\mu}$ and $\boldsymbol{m}$.

Table S9. Selected excited states for investigating the abilities of intersystem crossing.

	Excited State	E (eV)	EDD	ΔE_{ST} (eV)
**P-Et**	S_1	3.477	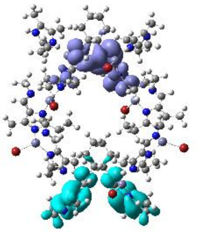	0
	T_9	3.477	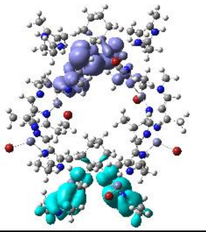	
**P-Et⊃PhBr**	S_1	3.531	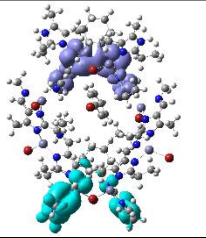	0
	T_9	3.531	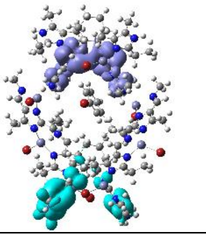	

Table S10. Atomic coordinates of optimized **P-Et** models at ground state and S_1 state.

S_0			
Atom	X	Y	Z
Zn	-2.7145	-4.5601	2.6912
Zn	-5.9398	2.846	1.0621
Zn	1.7598	-6.6506	-1.0408
Br	-2.2288	-4.1887	4.9812
Br	-5.4056	2.0364	3.231
Br	2.4275	-8.8648	-1.5955
N	-3.9499	-3.2142	1.7267
N	-1.3774	-5.5035	1.4701
N	-3.7174	-6.4662	2.5018
N	-4.4348	3.0694	-0.2953
N	-6.8697	1.868	-0.5623
N	-7.4666	-0.6755	0.4961
N	-2.8132	3.4088	-1.7767
N	3.2438	-5.261	-1.2053
N	0.2251	-6.5257	0.32
N	5.1156	-4.1326	-1.6311

continue			
N	0.9249	-5.4519	-2.6303
N	-5.5205	-2.5951	0.2729
C	-4.7835	2.4448	-1.4772
C	-3.2531	3.6286	-0.5102
C	-6.625	-0.803	1.4734
C	-8.4512	0.3795	0.5231
C	-3.7667	2.6534	-2.4
C	-6.0626	1.8249	-1.5764
C	-8.2015	1.3117	-0.6703
C	-5.6151	-1.7986	1.4045
C	-4.5097	-3.4219	0.5048
C	-4.6296	-2.1823	2.3038
H	-6.3506	1.3255	-2.5028
H	-6.6642	-0.1455	2.3445
C	-3.6727	2.1865	-3.8065
C	-4.3079	-1.6284	3.6412
C	-2.5303	4.4293	0.5192
C	-4.022	-4.4677	-0.4407
C	-4.7852	-4.5281	-1.7545
H	-2.9561	-4.285	-0.6255
H	-4.0581	-5.437	0.0721
H	-4.5713	-2.3296	4.4391
H	-4.8225	-0.6795	3.8018
H	-3.2327	-1.456	3.7347
C	-3.2118	5.767	0.8106
H	-2.4743	3.8367	1.4394
H	-1.5073	4.6049	0.1803
C	-9.8668	-0.1919	0.4848
H	-8.3326	0.976	1.4446
C	-9.2523	2.422	-0.71
H	-8.2769	0.7114	-1.592
H	-4.3663	-5.3052	-2.399
H	-4.7365	-3.5797	-2.297
H	-5.8427	-4.7706	-1.5979
C	-10.914	0.9149	0.4275
H	-9.9581	-0.8441	-0.394
H	-10.0147	-0.8314	1.3619
C	-10.6642	1.8465	-0.7555
H	-10.8803	1.4958	1.3596
H	-11.9167	0.4789	0.3684
H	-9.0598	3.0603	-1.5801
H	-9.1297	3.0541	0.1798
H	-10.7993	1.2889	-1.6928
H	-11.3994	2.6581	-0.7667
H	-3.8925	2.9974	-4.5109
H	-4.3763	1.3701	-3.9882
H	-2.6595	1.8451	-4.0344
H	-2.6487	6.3264	1.5627
H	-4.2279	5.6205	1.1917

continue			
H	-3.263	6.3811	-0.0944
C	-1.727	-6.8329	1.3116
C	3.0445	-4.4891	-2.335
C	1.8054	-4.5956	-3.0307
H	1.6192	-3.953	-3.8975
C	-0.7236	-7.4671	0.5935
C	4.215	-3.789	-2.5974
C	-2.9443	-7.2988	1.8885
H	-3.202	-8.3601	1.8087
C	-0.2074	-5.3615	0.869
C	4.493	-5.0279	-0.8224
C	0.5531	-4.0796	0.8003
H	0.6229	-3.7669	-0.2503
H	1.5868	-4.2721	1.1089
C	5.1192	-5.6953	0.3536
H	5.1151	-6.7771	0.17
H	6.1619	-5.3786	0.4264
C	-0.633	-8.8864	0.168
H	-0.6513	-8.979	-0.9223
H	0.3106	-9.3338	0.4903
C	4.4997	-2.8324	-3.6975
H	4.5504	-1.8019	-3.3285
H	3.7198	-2.8778	-4.4617
H	5.4695	-3.0413	-4.1553
C	-0.0571	-2.9748	1.6511
H	0.56	-2.0749	1.608
H	-0.1318	-3.2758	2.7015
H	-1.0579	-2.7024	1.2962
C	4.3978	-5.3917	1.6655
H	4.8996	-5.8896	2.4991
H	3.3592	-5.7398	1.6422
H	4.4003	-4.3164	1.8665
H	-1.4621	-9.4663	0.5808
C	-4.9203	-6.9245	3.1474
C	-0.3396	-5.5742	-3.3079
H	-0.4498	-4.8572	-4.1319
H	-5.7836	-6.3678	2.7628
H	-5.0977	-7.9976	2.9986
H	-4.8546	-6.7219	4.2219
H	-1.1582	-5.4162	-2.5949
H	-0.4479	-6.5885	-3.7077
Zn	-7.0079	-2.0203	-1.0505
Br	-6.4043	-1.236	-3.2306
Zn	-1.2085	4.3695	-2.6789
Br	-0.2245	3.2038	-4.4891
N	0.0779	5.3949	-1.4772
N	1.6617	6.4376	-0.3216
N	-2.0205	6.3564	-2.908
C	-0.1998	6.748	-1.4791

continue			
C	-1.3144	7.2074	-2.24
H	-1.5508	8.2765	-2.2569
C	0.7886	7.3958	-0.7498
C	1.8256	3.9236	-0.5338
H	2.8768	4.0689	-0.2795
H	1.7903	3.3481	-1.4651
C	0.9362	8.8399	-0.4387
H	0.7838	9.0289	0.6297
H	0.2093	9.4351	-0.9968
H	1.9455	9.1917	-0.6693
C	1.1983	5.2495	-0.7866
C	1.1286	3.1518	0.5882
H	1.6754	2.2334	0.8141
H	0.1085	2.8741	0.3044
H	1.0829	3.7505	1.5025
C	-3.1399	6.795	-3.7001
H	-3.3362	7.8705	-3.5986
H	-2.9573	6.5696	-4.7565
H	-4.0382	6.2419	-3.4011
Zn	7.2105	1.8264	1.0944
Zn	6.8416	-3.0624	-1.0761
Br	7.9554	0.7135	3.1026
Br	7.953	-2.195	-3.0299
N	8.6424	2.4544	-0.2195
N	6.4524	0.8329	-0.5995
N	7.8979	-3.8308	0.4927
N	6.0126	-1.8313	0.4299
N	10.3698	2.9125	-1.5939
N	9.1511	-4.5978	2.2012
C	8.4571	1.7671	-1.4094
C	7.5767	-3.112	1.635
C	6.5959	-2.0977	1.564
H	6.3485	-1.5328	2.4652
C	7.3114	0.9607	-1.5743
H	7.1569	0.4379	-2.5204
C	9.7931	3.1111	-0.3876
C	5.2316	0.0962	-0.8209
H	5.3412	-0.5335	-1.7206
C	2.7447	0.3184	-1.2254
H	2.8031	-0.312	-2.1236
H	1.9236	1.023	-1.3999
C	4.964	-0.8431	0.3609
H	4.9609	-0.2278	1.279
C	4.0623	1.0601	-1.0411
H	4.2861	1.6905	-1.9093
H	4.0033	1.7287	-0.1709
C	3.6033	-1.5378	0.2259
H	3.6607	-2.2442	-0.6099
H	3.4287	-2.1374	1.1271

continue			
C	2.4578	-0.5632	-0.0159
H	2.3167	0.0692	0.8731
H	1.5214	-1.114	-0.1616
C	9.5458	2.0661	-2.2406
C	10.4071	3.9914	0.6521
C	9.8184	1.5665	-3.6173
H	10.6511	4.9505	0.1791
H	11.3726	3.5554	0.9389
C	9.5468	4.2031	1.8891
H	9.3863	2.2288	-4.3775
H	9.4067	0.5634	-3.765
H	10.897	1.5331	-3.7872
H	10.0393	4.883	2.5904
H	9.3629	3.2601	2.414
H	8.5755	4.6414	1.6324
C	8.8389	-4.6935	0.8911
C	9.4984	-5.6808	-0.0161
C	8.3722	-3.606	2.6772
H	9.4742	-6.6589	0.4793
H	10.5618	-5.4182	-0.0887
C	8.8908	-5.7651	-1.4085
H	9.3996	-6.5269	-2.0061
H	8.9775	-4.8138	-1.9433
H	7.8293	-6.0346	-1.368
C	8.4136	-3.1617	4.0983
H	7.6147	-3.6262	4.6895
H	8.3034	-2.0755	4.1739
H	9.3677	-3.4539	4.542
Zn	3.3003	6.6808	0.9256
Br	4.2868	8.8439	0.9024
N	2.4528	6.0683	2.8034
N	4.4327	5.0049	1.243
N	5.7095	3.2087	1.5537
C	4.0822	4.4152	2.4458
C	3.0518	5.007	3.2306
H	2.7811	4.5506	4.1885
C	4.8788	3.2932	2.6303
C	6.0723	4.4995	-0.5686
H	7.1454	4.6433	-0.3973
H	6.0106	3.5748	-1.1556
C	5.4067	4.256	0.7432
C	4.8543	2.3046	3.7372
H	4.5014	1.3305	3.3795
H	4.1888	2.6331	4.5392
H	5.8572	2.1365	4.1385
C	5.4946	5.6721	-1.3457
H	6.0189	5.7859	-2.2976
H	5.5984	6.6136	-0.7962
H	4.4329	5.5226	-1.5756

continue			
C	1.4154	6.691	3.5832
H	1.7042	7.7196	3.8254
H	0.4913	6.7404	2.9943
H	1.2079	6.153	4.5175
N	-7.0906	4.6343	1.1359
N	-7.9845	6.5208	0.511
C	-7.167	5.5148	0.1436
C	-7.8972	5.0989	2.1669
C	-8.0835	4.3538	3.439
C	-6.5048	5.433	-1.1904
C	-6.923	6.536	-2.1537
H	-6.7324	4.4516	-1.6184
H	-5.4188	5.4443	-1.048
H	-8.5136	3.3627	3.259
H	-8.7575	4.8963	4.1052
H	-7.1334	4.193	3.9542
H	-6.4352	6.3958	-3.1207
H	-6.6329	7.5275	-1.7888
C	-8.4634	6.287	1.7851
H	-8.0034	6.5308	-2.3334
C	-9.3913	7.2243	2.4672
H	-8.9432	8.2154	2.5993
H	-10.3272	7.3495	1.9109
H	-9.6453	6.8454	3.4583
H	-8.2081	7.3182	-0.0626
N	-8.5935	-3.4141	-1.0914
N	-9.8959	-5.086	-0.5893
C	-9.6608	-3.4191	-1.9809
C	-7.9204	-4.8063	0.9118
H	-6.8808	-4.5695	0.6765
H	-7.9776	-5.8913	1.0572
C	-9.8041	-2.4158	-3.0683
H	-9.8979	-1.3995	-2.6685
H	-10.6959	-2.6209	-3.664
H	-8.9323	-2.4158	-3.7277
C	-8.7627	-4.4348	-0.2583
C	-8.3326	-4.0772	2.1935
H	-7.6592	-4.341	3.0134
H	-8.2821	-2.9953	2.0512
H	-9.3532	-4.336	2.4913
C	-10.4871	-4.4683	-1.6734
C	-11.7469	-4.9671	-2.2804
H	-12.0434	-4.3255	-3.1115
H	-11.6331	-5.9834	-2.6735
H	-12.5708	-4.9753	-1.5583
H	-10.2475	-5.9041	-0.1172
S_1			
Atom	X	Y	Z
Zn	-6.0017	2.794	1.0464

continue			
Zn	-7.0331	-2.0627	-1.1059
Br	-5.6707	2.0608	3.3354
Br	-6.4826	-1.5991	-3.373
N	-3.9393	-3.287	1.6402
N	-4.4417	3.0459	-0.2641
N	-7.015	4.7192	1.0953
N	-6.8763	1.8064	-0.4726
N	-7.8775	6.5397	0.2567
N	-7.4984	-0.7846	0.4789
N	-2.8297	3.4665	-1.7407
N	-8.681	-3.434	-0.9701
N	-5.5187	-2.6644	0.2013
N	-10.1355	-4.8286	-0.1384
C	-7.1349	5.444	-0.0103
C	-4.7764	2.3943	-1.4509
C	-3.2808	3.6732	-0.4897
C	-6.5689	-0.8125	1.3742
C	-8.4873	0.267	0.5167
C	-7.7091	5.3784	2.0985
C	-3.7588	2.6474	-2.3671
C	-6.0268	1.7367	-1.5456
C	-8.1865	1.2573	-0.6235
C	-5.5477	-1.8116	1.2924
C	-4.5412	-3.5257	0.445
C	-4.5589	-2.1895	2.1812
H	-6.3096	1.1926	-2.4403
C	-7.8104	4.8494	3.4837
H	-6.5515	-0.0993	2.2022
C	-3.6545	2.2092	-3.7841
C	-4.1704	-1.5835	3.4798
C	-6.5584	5.1458	-1.3508
C	-2.6194	4.543	0.528
C	-4.1259	-4.6306	-0.47
C	-5.0288	-4.8084	-1.6808
H	-3.0943	-4.4388	-0.7929
H	-4.079	-5.5594	0.1109
C	-7.0918	6.0325	-2.4673
H	-6.7474	4.0911	-1.575
H	-5.4673	5.2263	-1.2866
H	-4.446	-2.2236	4.3243
H	-4.6365	-0.6029	3.6036
H	-3.0859	-1.4536	3.5326
C	-3.3491	5.87	0.7417
H	-2.5719	3.9994	1.4793
H	-1.5906	4.7396	0.2163
C	-9.894	-0.3161	0.4136
H	-8.394	0.8212	1.4656
C	-9.2704	2.3403	-0.6582
H	-8.2522	0.6836	-1.5671

continue			
H	-4.6817	-5.6453	-2.2932
H	-5.0461	-3.9144	-2.3106
H	-6.0608	-5.0233	-1.3828
C	-10.9498	0.783	0.373
H	-9.9568	-0.9238	-0.4988
H	-10.0706	-0.9994	1.2528
C	-10.676	1.7598	-0.7672
H	-10.9413	1.3293	1.3265
H	-11.9477	0.3405	0.2749
H	-9.055	3.0112	-1.4975
H	-9.1813	2.9446	0.2556
H	-10.7771	1.231	-1.726
H	-11.4245	2.5606	-0.7728
H	-8.3166	3.8794	3.5033
H	-8.369	5.541	4.1189
H	-6.8229	4.686	3.9219
H	-4.0442	2.9582	-4.4897
H	-4.2336	1.2931	-3.9356
H	-2.6188	2.0052	-4.0704
H	-6.662	5.7217	-3.4227
H	-6.8286	7.0878	-2.324
H	-2.837	6.4867	1.4875
H	-4.3733	5.7003	1.0876
H	-3.3938	6.4398	-0.193
C	-9.798	-3.444	-1.7944
C	-8.0371	-4.5781	1.1915
H	-7.0149	-4.3088	0.9195
H	-8.0498	-5.6599	1.3759
C	-9.8988	-2.6025	-3.0157
H	-9.904	-1.5348	-2.7686
H	-10.8196	-2.8235	-3.5602
H	-9.0447	-2.7628	-3.6783
C	-8.9128	-4.2787	0.0251
C	-8.4457	-3.8214	2.4579
H	-7.7532	-4.0457	3.2738
H	-8.4217	-2.7441	2.2804
C	-8.2569	6.525	1.5852
H	-8.1808	5.9574	-2.5614
H	-9.455	-4.0923	2.7841
C	-10.7204	-4.3181	-1.2804
C	-9.0914	7.5979	2.1832
C	-12.0725	-4.7313	-1.7337
H	-8.6063	8.5789	2.1175
H	-10.0706	7.6759	1.6963
H	-9.2664	7.3895	3.2401
H	-12.3276	-4.2188	-2.6626
H	-12.1272	-5.809	-1.9254
H	-12.8437	-4.4811	-0.9963
H	-10.549	-5.5089	0.4787

H	-8.1174	7.2483	-0.4168

Table S11. Atomic coordinates of optimized **P-Et⊃PhF** models at ground state and S_1 state.

S_0			
Atom	X	Y	Z
Zn	-2.63875	-4.56072	2.730443
Zn	-5.87679	2.831817	1.065408
Zn	-6.9441	-2.04808	-1.01589
Zn	-1.15628	4.338866	-2.69578
Zn	7.274805	1.828029	1.072341
Zn	1.829187	-6.66781	-0.99986
Zn	3.358364	6.676788	0.884257
Zn	6.906559	-3.07385	-1.06851
Br	-1.95545	-4.03737	4.940056
Br	-5.26433	1.943213	3.182284
Br	8.002859	0.651107	3.046387
Br	2.43642	-8.88584	-1.61061
Br	-6.2179	-1.22721	-3.14579
Br	4.379352	8.814154	0.605177
Br	8.049461	-2.27372	-3.02499
Br	-0.11044	2.987851	-4.34406
N	-3.99359	-3.30017	1.827825
N	-1.33122	-5.49364	1.46884
N	-3.54139	-6.51744	2.709342
N	-4.43397	3.128595	-0.33151
N	-7.0897	4.566607	1.225655
N	-6.84445	1.871547	-0.55826
N	-8.05978	6.449767	0.717042
N	-7.42964	-0.69046	0.521242
N	-2.82342	3.470492	-1.82055
N	-5.54235	-2.67412	0.358305
N	8.72841	2.457923	-0.21972
N	3.313806	-5.28401	-1.18931
N	2.758107	6.276391	2.904631
N	0.321482	-6.51128	0.386736
N	5.195527	-4.17339	-1.61729
N	1.009565	-5.50119	-2.62965
N	4.528501	5.031362	1.226421
N	6.547475	0.83011	-0.63954
N	7.925809	-3.80033	0.534981
N	0.115196	5.362367	-1.47056
N	-8.5598	-3.39343	-1.15545
N	1.638744	6.403617	-0.23497

continue			
N	6.030879	-1.8212	0.400328
N	-1.91786	6.329657	-2.99421
N	5.833953	3.259463	1.565776
N	10.50603	2.878377	-1.54173
N	9.130239	-4.55683	2.28047
N	-9.91187	-5.05736	-0.7741
C	-7.16241	5.528841	0.312904
C	-4.81825	2.558625	-1.52972
C	-3.23397	3.649611	-0.5391
C	-6.63458	-0.85564	1.531084
C	-8.41002	0.370146	0.541929
C	-7.98147	4.886674	2.240417
C	-3.80558	2.767928	-2.45667
C	-6.0837	1.913413	-1.6082
C	-8.17009	1.298987	-0.65442
C	-5.64891	-1.87789	1.489878
C	-4.53583	-3.50373	0.600032
C	-4.67636	-2.26864	2.400493
H	-6.39767	1.454837	-2.5471
C	-8.18822	4.01352	3.424944
H	-6.68901	-0.20593	2.406748
C	-3.72172	2.330128	-3.87214
C	-4.36139	-1.71405	3.738679
C	-6.42624	5.603869	-0.98224
C	-2.44875	4.34291	0.519923
C	-4.03163	-4.54351	-0.34272
C	-4.74881	-4.57263	-1.68281
H	-2.95867	-4.36988	-0.48941
H	-4.09388	-5.52021	0.155102
C	-6.71435	6.866012	-1.78368
H	-6.67911	4.714824	-1.5713
H	-5.35455	5.520198	-0.77916
H	-4.56437	-2.4406	4.531207
H	-4.92903	-0.80089	3.923706
H	-3.29665	-1.47676	3.811893
C	-3.04141	5.695663	0.913178
H	-2.40322	3.684444	1.393521
H	-1.42554	4.47821	0.164114
C	-9.82589	-0.20411	0.517287
H	-8.28731	0.970647	1.460372
C	-9.23026	2.401731	-0.69574
H	-8.24514	0.694971	-1.57421
H	-4.31944	-5.34629	-2.32441
H	-4.66281	-3.61654	-2.20722
H	-5.81557	-4.79831	-1.5704
C	-10.8778	0.898044	0.460853
H	-9.92205	-0.86228	-0.35654
H	-9.96461	-0.83841	1.399742
C	-10.6398	1.82091	-0.73093

continue			
H	-10.8385	1.48656	1.388033
H	-11.8792	0.457413	0.413582
H	-9.04286	3.036445	-1.56945
H	-9.10717	3.038905	0.190217
H	-10.7782	1.255318	-1.66301
H	-11.3783	2.629484	-0.74423
H	-8.56188	3.026476	3.129388
H	-8.91759	4.455688	4.106769
H	-7.25434	3.846512	3.967476
H	-3.91099	3.160937	-4.56238
H	-4.44836	1.5394	-4.07405
H	-2.71799	1.956017	-4.09276
H	-6.1443	6.856989	-2.71512
H	-6.42309	7.76852	-1.23551
H	-2.42959	6.17041	1.684962
H	-4.0552	5.583282	1.312676
H	-3.07768	6.371364	0.052138
C	8.583421	1.743428	-1.39882
C	-1.57552	-6.85557	1.469577
C	3.139491	-4.5571	-2.35215
C	4.327616	4.572085	2.517458
C	7.543987	-3.10107	1.668816
C	-0.16944	6.714435	-1.47403
C	1.907925	-4.67805	-3.05867
H	1.741595	-4.07288	-3.95595
C	-0.53987	-7.48725	0.797259
C	3.416736	5.264594	3.364464
H	3.287314	4.927923	4.398128
C	4.316357	-3.86853	-2.61603
C	6.560703	-2.08902	1.558763
C	-1.25175	7.175217	-2.2794
H	6.271545	-1.52759	2.449352
H	-1.49957	8.241749	-2.28832
C	7.443362	0.935462	-1.58468
C	-2.73661	-7.34747	2.134048
H	7.322406	0.390313	-2.52325
H	-2.92468	-8.42592	2.153743
C	-9.59695	-3.33353	-2.07765
C	0.780116	7.360668	-0.69311
C	5.138488	3.463832	2.719486
C	9.887432	3.10589	-0.36139
C	-0.19204	-5.32832	0.816386
C	4.553307	-5.03416	-0.78683
C	-7.98047	-4.88851	0.80134
C	1.82732	3.897142	-0.4406
H	-6.92518	-4.69845	0.59498
H	2.914107	3.996132	-0.50405
H	-8.09459	-5.97227	0.918408
H	1.522078	3.20522	-1.2321

continue			
C	-9.68065	-2.27593	-3.11881
C	0.900601	8.801596	-0.35799
H	-9.77804	-1.27946	-2.67226
H	0.720229	8.96865	0.710007
H	-10.5488	-2.43671	-3.76123
H	0.176938	9.395566	-0.92133
H	-8.78096	-2.25839	-3.73981
H	1.910432	9.170047	-0.56226
C	6.008537	4.369935	-0.67512
H	7.095925	4.481667	-0.58463
H	5.875584	3.416891	-1.20061
C	5.444265	4.23052	0.698144
C	-8.77713	-4.44734	-0.37663
C	1.201492	5.216147	-0.72873
C	0.451271	-4.00327	0.573603
H	0.497217	-3.83135	-0.51006
H	1.497243	-4.06079	0.897869
C	5.2566	2.608827	3.926741
H	4.974727	1.575075	3.703971
H	4.611615	2.978978	4.727532
H	6.288976	2.565914	4.282699
C	5.140222	-5.6454	0.438493
H	5.139137	-6.73484	0.306526
H	6.181362	-5.33046	0.529381
C	5.421865	5.52027	-1.47798
H	5.887951	5.563271	-2.46538
H	5.590794	6.484318	-0.98783
H	4.343217	5.40224	-1.63636
C	-8.3843	-4.16996	2.091476
C	1.455016	3.354461	0.94021
H	-7.7433	-4.48682	2.918475
H	2.017779	2.4413	1.154412
H	0.390986	3.118853	1.014547
H	-8.27646	-3.08889	1.977861
H	1.696594	4.081166	1.718361
C	5.333865	0.094778	-0.90634
H	5.486229	-0.55273	-1.78732
C	2.880014	0.316619	-1.46558
H	2.993634	-0.33351	-2.34439
H	2.081785	1.025235	-1.71578
C	-0.33406	-8.93701	0.554478
H	-0.29085	-9.16594	-0.51396
H	0.621166	-9.26973	0.969787
C	4.996249	-0.82281	0.274511
H	4.954074	-0.19675	1.18413
C	4.185195	1.05784	-1.21311
H	4.46206	1.670342	-2.07872
H	4.072521	1.743092	-0.36086
C	4.627044	-2.95508	-3.74526

continue			
H	4.720077	-1.91724	-3.40681
H	3.840702	-2.99501	-4.50324
H	5.585268	-3.21175	-4.20322
C	-0.26101	-2.85004	1.267299
H	0.256896	-1.90843	1.074619
H	-0.28899	-2.99539	2.352794
H	-1.28755	-2.72911	0.904178
C	3.637674	-1.50747	0.077881
H	3.738476	-2.23608	-0.73425
H	3.403727	-2.08284	0.981237
C	2.512624	-0.53644	-0.25772
H	2.312507	0.116407	0.604578
H	1.587659	-1.09069	-0.45111
C	4.372933	-5.27735	1.707473
H	4.833837	-5.74699	2.580242
H	3.329534	-5.60821	1.656802
H	4.385161	-4.19482	1.863943
C	-1.63997	0.786993	0.449282
C	-0.73161	0.791044	-0.59721
C	-2.88437	0.184379	0.351041
F	-1.31272	1.41001	1.598575
C	-1.082	0.165357	-1.79147
H	0.229208	1.275116	-0.47412
C	-3.21932	-0.4351	-0.85012
H	-3.56638	0.223267	1.193948
C	-2.32549	-0.44876	-1.91948
H	-0.38748	0.191854	-2.62543
H	-4.19272	-0.8974	-0.9627
H	-2.61424	-0.92054	-2.85371
C	9.70261	2.019469	-2.19744
C	10.46641	4.005237	0.681699
C	10.0212	1.487999	-3.55204
H	10.71542	4.96052	0.203313
H	11.42696	3.580968	1.000812
C	9.568401	4.225555	1.890145
H	9.637463	2.146223	-4.34121
H	9.592894	0.491974	-3.69997
H	11.10494	1.426497	-3.67567
H	10.03432	4.918373	2.596991
H	9.378415	3.286476	2.420577
H	8.601775	4.650568	1.597203
C	-8.59587	6.072366	1.932804
H	-7.77251	6.946819	-2.05589
H	-1.13659	-9.52114	1.011388
H	-9.42298	-4.38435	2.360549
C	-10.4544	-4.37715	-1.84635
C	8.874833	-4.64322	0.9593
C	9.600337	-5.57501	0.045171
C	8.306904	-3.58823	2.737011

continue			
H	10.20505	-6.23804	0.670016
H	10.29848	-4.99309	-0.57133
C	8.679341	-6.37997	-0.86819
H	9.257556	-7.03287	-1.52881
H	8.075998	-5.72468	-1.50483
H	7.994564	-7.0064	-0.28761
C	8.283162	-3.16172	4.163719
H	7.448133	-3.61846	4.709412
H	8.186866	-2.07448	4.246008
H	9.209723	-3.47369	4.650073
C	-4.69097	-6.996	3.433117
C	1.851005	7.006336	3.752079
C	-0.24685	-5.63707	-3.31939
C	-3.01574	6.768071	-3.81601
H	-5.60071	-6.52671	3.03899
H	-4.80352	-8.08635	3.374485
H	-4.60369	-6.706	4.485848
C	-11.7036	-4.81843	-2.51663
H	-11.9636	-4.12662	-3.31923
H	-11.5957	-5.81382	-2.96161
H	-12.549	-4.85025	-1.82049
C	-9.6127	6.89096	2.64053
H	-9.22754	7.88378	2.898448
H	-10.5182	7.027175	2.03872
H	-9.90561	6.401952	3.570939
H	-8.29055	7.288561	0.209011
H	-10.2958	-5.88807	-0.35185
H	-3.23302	7.837754	-3.69752
H	-2.79389	6.570376	-4.87045
H	-3.91398	6.19287	-3.56048
H	2.170616	8.052136	3.816634
H	0.845785	6.995747	3.314043
H	1.795308	6.590334	4.766347
H	-0.33369	-4.95939	-4.17884
H	-0.36526	-6.66795	-3.67073
H	-1.07293	-5.43124	-2.62735
S_1			
Atom	X	Y	Z
Zn	-2.61501	-4.59156	2.736704
Zn	-5.92262	2.785319	1.140346
Zn	-6.97076	-2.09096	-0.9591
Zn	-1.24563	4.348831	-2.65204
Zn	7.235753	1.881176	1.030769
Zn	1.83689	-6.6441	-1.04285
Zn	3.281463	6.701089	0.9028
Zn	6.886295	-3.01146	-1.13424
Br	-1.44304	-4.03592	4.743685
Br	-5.09393	2.17621	3.278091
Br	7.98719	0.538199	2.917213

continue			
Br	2.921833	-8.75436	-1.26394
Br	-6.12283	-1.41777	-3.15418
Br	4.357061	8.819279	0.725326
Br	8.246685	-2.16766	-2.8715
Br	-0.02404	3.031915	-4.223
N	-4.06358	-3.46558	1.885592
N	-1.34765	-5.5385	1.396098
N	-3.31152	-6.63466	2.943199
N	-4.48345	3.131861	-0.28293
N	-7.22409	4.471192	1.240443
N	-6.86716	1.829603	-0.51548
N	-8.42428	6.165872	0.582321
N	-7.49246	-0.80242	0.49731
N	-2.86479	3.472692	-1.76179
N	-5.60018	-2.79553	0.419413
N	8.710631	2.463944	-0.24317
N	3.326788	-5.21253	-1.23971
N	2.682631	6.232339	2.913269
N	0.256204	-6.49713	0.199783
N	5.229833	-4.12454	-1.61279
N	1.162944	-5.60554	-2.82717
N	4.490989	5.049953	1.233972
N	6.515752	0.851552	-0.68115
N	7.917474	-3.62322	0.603633
N	0.067818	5.3999	-1.4687
N	-8.60427	-3.45426	-1.20674
N	1.604861	6.438497	-0.24956
N	5.975915	-1.73734	0.390816
N	-2.00923	6.352032	-2.9295
N	5.82134	3.292686	1.551522
N	10.46743	2.921963	-1.57881
N	9.145002	-4.31125	2.399386
N	-10.066	-4.99486	-0.71677
C	-7.41204	5.336738	0.253633
C	-4.79357	2.441356	-1.43589
C	-3.3209	3.724339	-0.50821
C	-6.62545	-0.90426	1.551306
C	-8.44508	0.262062	0.510699
C	-8.15639	4.752074	2.227186
C	-3.78169	2.644956	-2.35704
C	-6.04838	1.756555	-1.51134
C	-8.18717	1.243749	-0.64201
C	-5.66894	-1.94724	1.531403
C	-4.62438	-3.67807	0.682759
C	-4.70458	-2.36823	2.441401
H	-6.30345	1.194369	-2.41243
C	-8.26108	3.941439	3.468479
H	-6.70223	-0.23808	2.404144
C	-3.63923	2.101959	-3.73221

continue			
C	-4.39489	-1.81752	3.786839
C	-6.68083	5.389114	-1.04474
C	-2.62131	4.557034	0.511941
C	-4.1768	-4.75537	-0.25665
C	-4.98375	-4.83506	-1.54446
H	-3.11908	-4.59428	-0.50038
H	-4.20656	-5.72125	0.26526
C	-7.16572	6.483601	-1.98502
H	-6.77713	4.408264	-1.52219
H	-5.61203	5.50624	-0.84106
H	-4.9846	-2.2968	4.580653
H	-4.61583	-0.74607	3.817127
H	-3.34249	-1.95313	4.047933
C	-3.33338	5.878332	0.796531
H	-2.5543	3.967356	1.431614
H	-1.59974	4.747002	0.174848
C	-9.87811	-0.27347	0.425581
H	-8.35664	0.843108	1.449265
C	-9.24003	2.353688	-0.69179
H	-8.23462	0.666743	-1.58101
H	-4.61151	-5.6448	-2.17902
H	-4.92324	-3.90405	-2.11578
H	-6.04345	-5.0298	-1.34905
C	-10.9268	0.830359	0.367618
H	-9.95163	-0.9072	-0.46966
H	-10.0455	-0.93558	1.281667
C	-10.6489	1.780401	-0.79332
H	-10.9102	1.39968	1.308195
H	-11.9317	0.401189	0.280955
H	-9.02482	3.018503	-1.53784
H	-9.15534	2.961096	0.21895
H	-10.7528	1.234361	-1.74172
H	-11.3844	2.592895	-0.81712
H	-8.58625	2.919325	3.243468
H	-8.98514	4.379768	4.158785
H	-7.29351	3.857965	3.970077
H	-3.87536	2.855698	-4.4935
H	-4.30528	1.24688	-3.87297
H	-2.61153	1.773821	-3.90776
H	-6.59635	6.457518	-2.91704
H	-7.03313	7.481479	-1.55215
H	-2.78407	6.45943	1.542937
H	-4.34113	5.703281	1.187396
H	-3.41292	6.486869	-0.11105
C	8.536753	1.799756	-1.44793
C	-1.48828	-6.90828	1.489681
C	3.214848	-4.53853	-2.43704
C	4.290302	4.567495	2.514525
C	7.511286	-2.93581	1.681521

continue			
C	-0.22518	6.748097	-1.45497
C	2.025547	-4.73618	-3.21665
H	1.872981	-4.14997	-4.12842
C	-0.48992	-7.5079	0.743385
C	3.350177	5.227834	3.364746
H	3.208515	4.866622	4.388417
C	4.396849	-3.85795	-2.67281
C	6.451584	-1.95243	1.557753
C	-1.34272	7.200082	-2.22218
H	6.110121	-1.4195	2.443314
H	-1.61967	8.259267	-2.19896
C	7.391073	1.01005	-1.64301
C	-2.53893	-7.43982	2.305932
H	7.238787	0.526251	-2.60998
H	-2.66168	-8.5259	2.379696
C	-9.60119	-3.43208	-2.16938
C	0.730937	7.395419	-0.68557
C	5.115447	3.47196	2.707338
C	9.869675	3.110203	-0.37887
C	-0.30225	-5.32881	0.619945
C	4.541204	-4.95513	-0.78508
C	-8.19574	-4.75378	0.922476
C	1.804612	3.934219	-0.48905
H	-7.12376	-4.62063	0.758973
H	2.890885	4.04973	-0.53327
H	-8.37052	-5.81384	1.143531
H	1.517947	3.266821	-1.30844
C	-9.59599	-2.45969	-3.29385
C	0.852141	8.834309	-0.33863
H	-9.76843	-1.4383	-2.93352
H	0.674167	8.995325	0.730846
H	-10.3812	-2.69577	-4.01583
H	0.125426	9.431433	-0.89444
H	-8.62966	-2.45357	-3.80489
H	1.858744	9.208324	-0.54716
C	5.986127	4.445404	-0.67363
H	7.071621	4.571733	-0.57906
H	5.860941	3.504507	-1.22294
C	5.418949	4.272743	0.695981
C	-8.91185	-4.40451	-0.33522
C	1.165607	5.253047	-0.74856
C	0.19404	-3.977	0.218412
H	0.039743	-3.85727	-0.86307
H	1.281324	-3.94496	0.364332
C	5.251174	2.601901	3.903351
H	4.967082	1.569382	3.675919
H	4.612355	2.957468	4.714896
H	6.284927	2.56777	4.256585
C	5.06035	-5.564	0.472737

continue			
H	5.01175	-6.65342	0.350478
H	6.112806	-5.30236	0.591414
C	5.388793	5.606624	-1.4536
H	5.860506	5.679288	-2.43656
H	5.54182	6.560078	-0.93848
H	4.313576	5.477234	-1.62084
C	-8.62447	-3.87613	2.101724
C	1.411704	3.34484	0.86634
H	-8.06189	-4.15195	2.997547
H	1.97232	2.425716	1.061886
H	0.348317	3.103746	0.912529
H	-8.41238	-2.82711	1.882562
H	1.635548	4.046597	1.672616
C	5.295319	0.137626	-0.97625
H	5.455776	-0.51976	-1.84967
C	2.856135	0.344241	-1.5956
H	2.992489	-0.29515	-2.47917
H	2.056404	1.045907	-1.851
C	-0.21488	-8.95031	0.517673
H	-0.33064	-9.21935	-0.53747
H	0.811998	-9.20868	0.789548
C	4.912915	-0.76171	0.202616
H	4.826355	-0.13163	1.102009
C	4.150457	1.094614	-1.31036
H	4.450461	1.704548	-2.16928
H	4.014954	1.782938	-0.46485
C	4.779606	-2.99422	-3.82015
H	4.910262	-1.94938	-3.51662
H	4.01061	-3.02008	-4.59526
H	5.729153	-3.314	-4.25771
C	-0.48567	-2.83745	0.965248
H	-0.10918	-1.87015	0.625167
H	-0.31591	-2.91165	2.04422
H	-1.56405	-2.82944	0.785435
C	3.586117	-1.48798	-0.04028
H	3.723144	-2.20735	-0.85497
H	3.326416	-2.06885	0.851779
C	2.465751	-0.5205	-0.40364
H	2.231695	0.121278	0.456029
H	1.55522	-1.08462	-0.62263
C	4.260428	-5.15818	1.710046
H	4.655178	-5.64828	2.604113
H	3.210148	-5.4461	1.61123
H	4.292066	-4.07599	1.86919
C	-1.7556	0.881687	0.288061
C	-0.9057	0.870402	-0.8074
C	-2.88668	0.085858	0.362587
F	-1.48169	1.723041	1.30882
C	-1.19996	0.023331	-1.87383

continue			
H	-0.04609	1.529754	-0.8326
C	-3.16687	-0.75361	-0.71286
H	-3.53445	0.132296	1.231656
C	-2.33009	-0.78962	-1.82713
H	-0.55812	0.039985	-2.74993
H	-4.05458	-1.37358	-0.67739
H	-2.58686	-1.42883	-2.66626
C	9.64657	2.101567	-2.25568
C	10.46656	3.974403	0.682221
C	9.937103	1.631255	-3.63843
H	10.67675	4.954906	0.237837
H	11.44767	3.559665	0.945552
C	9.606401	4.122513	1.92834
H	9.48464	2.291674	-4.38771
H	9.555651	0.619264	-3.80433
H	11.0162	1.634297	-3.80589
H	10.08296	4.791958	2.649708
H	9.452567	3.15737	2.42252
H	8.623432	4.542573	1.687626
C	-8.91751	5.819319	1.826153
H	-8.22102	6.354762	-2.24975
H	-0.89966	-9.56803	1.103444
H	-9.69296	-3.98678	2.315006
C	-10.5271	-4.39869	-1.87429
C	8.901648	-4.43813	1.06193
C	9.644539	-5.39289	0.215902
C	8.299656	-3.3897	2.823662
H	9.726115	-6.32394	0.789924
H	10.6766	-5.01742	0.151605
C	9.066469	-5.62226	-1.17143
H	9.641268	-6.39016	-1.693
H	9.105789	-4.71355	-1.77953
H	8.028369	-5.96681	-1.12232
C	8.196553	-2.92394	4.219142
H	7.198203	-3.13072	4.621542
H	8.337837	-1.83803	4.257931
H	8.942774	-3.42449	4.834546
C	-4.35762	-7.13689	3.795568
C	1.733991	6.92189	3.750942
C	-0.06026	-5.80952	-3.56426
C	-3.16439	6.77039	-3.68121
H	-5.31747	-6.71133	3.483666
H	-4.42833	-8.23242	3.778896
H	-4.17222	-6.80696	4.82333
C	-11.7789	-4.82869	-2.54726
H	-11.9389	-4.23754	-3.45062
H	-11.7416	-5.88284	-2.84532
H	-12.6567	-4.69286	-1.90486
C	-10.0447	6.545189	2.464196

continue			
H	-9.80905	7.602569	2.630382
H	-10.9576	6.494945	1.859643
H	-10.2698	6.102162	3.435748
H	-8.76337	6.91806	0.005012
H	-10.5137	-5.75063	-0.22439
H	-3.38976	7.837094	-3.55303
H	-3.0075	6.56813	-4.74613
H	-4.03312	6.18235	-3.36249
H	2.020599	7.975631	3.832775
H	0.741943	6.883369	3.287291
H	1.669173	6.490271	4.757547
H	-0.14285	-5.15065	-4.43721
H	-0.11468	-6.85115	-3.89682
H	-0.91494	-5.63109	-2.90305

Table S12. Atomic coordinates of optimized **P-Et⊃PhBr** models at ground state and S_1 state.

S_0			
Atom	X	Y	Z
Zn	-6.8964	-2.0107	-1.0595
Zn	-5.8833	2.8334	0.997
Br	-6.3952	-1.2321	-3.2701
Br	-5.4041	2.0261	3.1811
N	-7.3825	-0.6636	0.4713
N	-5.4024	-2.5484	0.2588
C	-5.5531	-1.7941	1.4146
C	-6.5758	-0.8088	1.4747
C	-8.3785	0.3824	0.4852
C	-4.3777	-3.3584	0.4946
C	-4.5846	-2.1846	2.3304
H	-6.6437	-0.1663	2.3547
H	-8.2589	0.995	1.3957
C	-8.1478	1.2962	-0.7261
C	-9.7884	-0.2047	0.4692
N	-3.8645	-3.1829	1.7407
C	-3.8204	-4.3409	-0.4792
C	-4.2921	-1.6392	3.6764
N	-6.8221	1.8685	-0.6348
H	-8.2224	0.6795	-1.6371
C	-9.212	2.3935	-0.7785
H	-9.8804	-0.8746	-0.3961
H	-9.9223	-0.8282	1.36
C	-10.8474	0.8903	0.3997
H	-2.7892	-4.0392	-0.7037
H	-3.7332	-5.3129	0.021
C	-4.621	-4.4684	-1.7655
H	-4.3015	-2.4211	4.4408

continue			
H	-4.9972	-0.8489	3.9377
H	-3.2877	-1.2046	3.6847
C	-6.0452	1.8864	-1.673
H	-9.0312	3.0177	-1.661
H	-9.0909	3.0437	0.0982
C	-10.6175	1.8016	-0.8029
H	-10.8112	1.4895	1.3202
H	-11.846	0.4428	0.3585
H	-4.1623	-5.2108	-2.4236
H	-4.6644	-3.5225	-2.3129
H	-5.6497	-4.7934	-1.5719
N	-4.3782	3.0326	-0.3579
C	-4.7649	2.502	-1.5733
H	-6.3544	1.4293	-2.6144
H	-10.7536	1.2247	-1.7282
H	-11.3619	2.6044	-0.8234
C	-3.1746	3.5518	-0.5482
C	-3.7499	2.7339	-2.4931
N	-2.7609	3.4055	-1.8337
C	-2.3971	4.2294	0.5256
C	-3.6801	2.3667	-3.9298
H	-2.3991	3.5796	1.4074
H	-1.3611	4.3299	0.1971
C	-2.956	5.6057	0.887
H	-3.9276	3.2204	-4.5724
H	-4.3767	1.5551	-4.1545
H	-2.6672	2.051	-4.1941
H	-2.3487	6.0704	1.6684
H	-3.9825	5.5305	1.2619
H	-2.9486	6.2714	0.0176
Zn	-2.6281	-4.5503	2.6868
Zn	1.8163	-6.6619	-1.0406
Br	-2.141	-4.1996	4.9811
Br	2.3699	-8.8636	-1.7512
N	-1.2985	-5.4936	1.4648
N	-3.6191	-6.464	2.5297
C	-1.6072	-6.8397	1.3791
C	-2.82	-7.3065	1.9651
C	-0.1328	-5.3467	0.8572
C	-4.8166	-6.921	3.1862
C	-0.5807	-7.4819	0.703
H	-3.0522	-8.376	1.9336
N	0.338	-6.527	0.3753
C	0.5789	-4.0425	0.7134
C	-0.4336	-8.924	0.3838
H	0.652	-3.7996	-0.3552
H	1.6147	-4.1736	1.0475
C	-0.0895	-2.906	1.4745
H	-0.3463	-9.0935	-0.6929

continue			
H	-1.2852	-9.4918	0.7664
H	0.482	-9.3281	0.8248
N	3.3224	-5.2989	-1.2055
N	0.9838	-5.4191	-2.6051
C	3.1388	-4.5298	-2.3398
C	1.8901	-4.5997	-3.0235
H	0.4793	-1.9804	1.366
H	-0.1541	-3.1275	2.5454
H	-1.0983	-2.7052	1.0978
C	4.5737	-5.0836	-0.8198
C	-0.2907	-5.5032	-3.2692
C	4.3223	-3.8535	-2.6047
H	1.7178	-3.958	-3.8939
N	5.2142	-4.206	-1.6332
C	5.1794	-5.7445	0.3705
C	4.6275	-2.9107	-3.7112
H	5.1652	-6.8286	0.2016
H	6.2251	-5.4397	0.4518
C	4.4448	-5.4124	1.6684
H	4.7063	-1.8796	-3.3493
H	3.8449	-2.9423	-4.4734
H	5.5905	-3.1468	-4.17
H	4.9238	-5.9107	2.5151
H	3.3992	-5.7377	1.6324
H	4.4668	-4.335	1.8571
H	-0.4374	-6.5168	-3.6583
H	-0.3844	-4.7895	-4.0982
H	-1.0958	-5.3103	-2.5493
H	-4.964	-8.0046	3.0904
H	-4.7663	-6.6634	4.2498
H	-5.6903	-6.4084	2.7659
Zn	-1.0965	4.311	-2.6785
Br	-0.0616	3.0946	-4.4375
N	0.1555	5.3314	-1.4352
N	-1.8593	6.2994	-2.985
C	-0.1231	6.6848	-1.4526
C	-1.193	7.1472	-2.2742
C	1.2445	5.1886	-0.696
C	-2.9237	6.7391	-3.8485
C	0.8321	7.335	-0.6828
H	-1.4253	8.2169	-2.3027
N	1.6888	6.3795	-0.2178
C	1.8646	3.8675	-0.4026
C	0.9609	8.7791	-0.365
H	2.9518	3.9753	-0.4078
H	1.6041	3.1888	-1.2212
C	1.4269	3.2877	0.9438
H	0.7883	8.9592	0.7022
H	0.2357	9.3696	-0.93

continue			
H	1.9704	9.1418	-0.58
H	1.9874	2.3746	1.1643
H	0.3618	3.0404	0.9547
H	1.6176	3.9968	1.7523
H	-2.653	6.5551	-4.8941
H	-3.8262	6.1526	-3.6406
H	-3.1554	7.8055	-3.7277
Zn	6.9412	-3.1489	-1.0681
Zn	7.3035	1.8211	1.0711
Br	8.0731	-2.2925	-3.0155
Br	8.0608	0.678	3.0585
N	7.9735	-3.9047	0.5206
N	6.1157	-1.8813	0.4127
C	7.6534	-3.1641	1.6492
C	6.6871	-2.1373	1.5551
C	8.9055	-4.7686	0.9373
C	5.0827	-0.8786	0.3178
C	8.44	-3.6477	2.7028
H	6.4414	-1.5544	2.4451
N	9.2127	-4.6539	2.2467
C	9.559	-5.777	0.0489
C	5.3871	0.0481	-0.8654
H	5.0678	-0.2576	1.2319
C	3.7177	-1.5599	0.158
C	8.4798	-3.1803	4.1165
H	9.5052	-6.7512	0.5501
H	10.6287	-5.5378	-0.0072
C	8.9727	-5.859	-1.3527
N	6.5989	0.7917	-0.6205
H	5.5222	-0.5905	-1.7552
C	4.2299	1.0129	-1.1304
H	3.7904	-2.2804	-0.6644
H	3.5127	-2.144	1.0628
C	2.5841	-0.5846	-0.1313
H	7.6757	-3.6295	4.7125
H	8.3769	-2.0923	4.1739
H	9.4304	-3.4717	4.5681
H	9.4767	-6.6339	-1.9375
H	9.0857	-4.9128	-1.8917
H	7.9058	-6.1085	-1.328
C	7.4856	0.9019	-1.5732
C	2.9146	0.2789	-1.3424
H	4.4799	1.6305	-2.0005
H	4.1477	1.693	-0.2707
H	2.413	0.06	0.7437
H	1.6528	-1.136	-0.3013
N	8.7537	2.4542	-0.226
C	8.615	1.7275	-1.3986
H	7.3651	0.3485	-2.5068

continue			
H	2.9906	-0.3622	-2.2318
H	2.1144	0.9973	-1.5518
C	9.9029	3.1173	-0.3776
C	9.7271	2.0128	-2.2037
N	10.5208	2.8881	-1.558
C	10.4735	4.0342	0.6549
C	10.048	1.4747	-3.5551
C	9.578	4.254	1.8651
H	10.039	4.9575	2.5646
H	9.4001	3.3172	2.4035
H	8.6057	4.6667	1.5733
H	10.7082	4.9881	0.1666
H	11.4408	3.6256	0.9739
H	9.672	2.1331	-4.3477
H	11.1319	1.4058	-3.6746
H	9.6143	0.4809	-3.7018
Zn	3.4108	6.6705	0.8946
Br	4.432	8.8056	0.6241
N	2.7734	6.2455	2.9017
N	4.5605	5.0151	1.2326
C	4.3304	4.5313	2.5095
C	3.4082	5.2149	3.3522
C	1.8527	6.9648	3.7435
C	5.4766	4.2159	0.7033
C	5.127	3.4115	2.7032
H	3.2506	4.8549	4.3741
N	5.8424	3.2256	1.5585
C	6.0539	4.3691	-0.6631
C	5.2092	2.5238	3.8896
H	7.1383	4.4996	-0.5634
H	5.9416	3.4148	-1.1914
C	5.4538	5.51	-1.4695
H	4.8996	1.5048	3.6357
H	4.5649	2.8897	4.6929
H	6.2364	2.4425	4.2538
H	5.9208	5.5569	-2.4562
H	5.6108	6.4775	-0.9819
H	4.377	5.377	-1.6282
H	0.8611	6.9826	3.2757
H	1.7608	6.521	4.7433
H	2.1851	8.0034	3.8477
Br	-1.5667	0.728	1.9805
C	-1.7149	0.3235	0.1369
C	-0.6505	0.6091	-0.7104
C	-2.8836	-0.2609	-0.3367
C	-0.7567	0.2912	-2.0619
H	0.2491	1.0662	-0.3173
C	-2.9747	-0.5794	-1.6896
H	-3.7059	-0.4579	0.3411

continue			
C	-1.914	-0.3089	-2.5503
H	0.0567	0.5386	-2.7361
H	-3.8921	-1.013	-2.0761
H	-1.9954	-0.5502	-3.6056
N	-9.725	-5.126	-0.5133
C	-10.3468	-4.5293	-1.5916
C	-11.6111	-5.0534	-2.167
C	-8.5945	-4.4553	-0.2129
C	-9.5416	-3.4727	-1.9283
N	-8.4554	-3.4423	-1.0612
C	-7.7308	-4.8023	0.9489
C	-9.7236	-2.4882	-3.0271
H	-6.7037	-4.5184	0.7126
H	-7.7398	-5.8906	1.0819
C	-8.1719	-4.1078	2.2404
H	-9.7836	-1.463	-2.6449
H	-10.6462	-2.6926	-3.5742
H	-8.8862	-2.5131	-3.729
H	-7.4827	-4.3466	3.0547
H	-9.1774	-4.4187	2.5397
H	-8.1751	-3.0233	2.1094
H	-10.056	-5.9426	-0.0239
H	-11.4874	-6.0684	-2.5605
H	-12.417	-5.0755	-1.425
H	-11.9399	-4.4198	-2.992
N	-7.9656	6.4979	0.4627
C	-8.5635	6.1618	1.6611
C	-9.5937	7.0184	2.3013
C	-7.0713	5.5487	0.1217
C	-7.9892	4.972	2.0247
N	-7.0596	4.6078	1.0592
C	-6.278	5.5804	-1.1411
C	-8.2754	4.1352	3.2189
H	-6.4918	4.6639	-1.7021
H	-5.2139	5.5255	-0.8904
C	-6.5539	6.8024	-2.0072
H	-8.6707	3.1544	2.9307
H	-9.0173	4.619	3.8576
H	-7.3715	3.9496	3.8039
H	-5.9573	6.7595	-2.9205
H	-6.2884	7.7324	-1.4929
H	-7.6045	6.8575	-2.3126
H	-8.1571	7.3267	-0.0768
H	-10.4688	7.1561	1.6563
H	-9.9376	6.5603	3.2299
H	-9.1999	8.0101	2.5504
S_1			
Atom	X	Y	Z
Zn	-6.9263	-2.052	-0.9986

continue			
Zn	-2.6051	-4.5727	2.6995
Zn	-5.9351	2.7913	1.0705
Zn	1.824	-6.6314	-1.0754
Zn	-1.1927	4.3241	-2.6396
Zn	3.326	6.7052	0.9047
Br	-6.3287	-1.443	-3.2895
Br	-1.6889	-4.2121	4.8713
Br	-5.2685	2.3277	3.3051
Br	2.8216	-8.7631	-1.4368
Br	0.0163	3.1693	-4.342
Br	4.4033	8.8197	0.7345
N	-7.4422	-0.7685	0.4566
N	-5.423	-2.6259	0.2955
C	-5.5228	-1.8076	1.4273
C	-6.5203	-0.7992	1.4668
C	-8.4094	0.2825	0.4544
C	-4.4383	-3.5	0.5494
C	-4.5571	-2.2314	2.3362
H	-6.6164	-0.1501	2.3309
H	-8.3324	0.8745	1.3869
C	-8.161	1.2538	-0.7122
C	-9.8339	-0.2715	0.3654
N	-3.8949	-3.3097	1.7648
C	-3.9397	-4.5166	-0.4291
C	-4.2334	-1.6842	3.6795
N	-6.8412	1.8352	-0.5934
H	-8.2148	0.6668	-1.6445
C	-9.2172	2.3593	-0.7634
H	-9.8971	-0.9089	-0.5283
H	-9.9998	-0.932	1.223
C	-10.8926	0.8228	0.2991
H	-2.9802	-4.1692	-0.8382
H	-3.7121	-5.4436	0.1084
C	-4.8985	-4.8025	-1.5769
H	-4.6738	-2.2725	4.4949
H	-4.6027	-0.6581	3.7629
H	-3.1541	-1.6632	3.8522
C	-6.0028	1.7183	-1.5692
H	-9.0066	3.023	-1.6114
H	-9.1347	2.9707	0.1447
C	-10.6217	1.7744	-0.8626
H	-10.8851	1.3934	1.239
H	-11.8927	0.3834	0.2098
N	-1.2959	-5.5267	1.4251
N	-3.459	-6.5735	2.7063
C	-1.5451	-6.8833	1.3848
C	-2.6915	-7.3914	2.0793
H	-4.4923	-5.5811	-2.2295
H	-5.0716	-3.9138	-2.1913

continue			
H	-5.8715	-5.1469	-1.2118
N	-4.409	3.0136	-0.2948
C	-4.729	2.3666	-1.4698
H	-6.2543	1.1471	-2.466
H	-10.7215	1.2271	-1.8106
H	-11.364	2.5805	-0.8859
C	-0.2013	-5.3306	0.718
C	-4.6039	-7.0489	3.4381
C	-0.5582	-7.4902	0.6292
H	-2.8931	-8.468	2.0651
C	-3.2396	3.5992	-0.5017
C	-3.719	2.596	-2.3881
N	0.2868	-6.4962	0.2149
C	0.4209	-3.9924	0.4814
C	-0.3792	-8.9229	0.2786
N	-2.7872	3.3864	-1.7647
C	-2.5377	4.4016	0.5402
C	-3.6111	2.1458	-3.8002
H	0.3393	-3.7525	-0.588
H	1.4965	-4.0678	0.6828
C	-0.2072	-2.8837	1.3149
H	-0.5056	-9.092	-0.7961
H	-1.108	-9.5419	0.8072
H	0.6265	-9.2708	0.5289
H	-2.4934	3.7968	1.4524
H	-1.5104	4.5838	0.2168
C	-3.2311	5.7316	0.8342
H	-3.9506	2.9234	-4.4961
H	-4.2207	1.2531	-3.9625
H	-2.5746	1.9113	-4.0532
H	0.2964	-1.9304	1.142
H	-0.1469	-3.1066	2.3851
H	-1.2582	-2.7347	1.0514
N	0.099	5.3737	-1.4371
N	-1.9706	6.322	-2.9126
C	-0.1884	6.7231	-1.4389
C	-1.2999	7.1744	-2.2157
H	-2.6894	6.2866	1.6056
H	-4.2521	5.571	1.1948
H	-3.2733	6.3582	-0.0636
C	1.2015	5.2315	-0.723
C	-3.1058	6.7408	-3.6931
C	0.7759	7.376	-0.6856
H	-1.564	8.2371	-2.2123
N	1.6492	6.4213	-0.2431
C	1.8284	3.9086	-0.4521
C	0.9065	8.8189	-0.3591
H	2.9143	4.0263	-0.4246
H	1.5932	3.2557	-1.2994

continue			
C	1.3502	3.2869	0.8607
H	0.7377	8.9956	0.7094
H	0.1781	9.4119	-0.9171
H	1.9127	9.1861	-0.5806
H	1.8837	2.3531	1.062
H	0.2815	3.0602	0.8376
H	1.5327	3.9617	1.7004
H	-4.739	-8.1354	3.3556
H	-4.4898	-6.7816	4.494
H	-5.5054	-6.5469	3.0698
H	-2.9255	6.5267	-4.7521
H	-3.9847	6.1613	-3.3889
H	-3.3274	7.81	-3.5803
Zn	6.9195	-3.0763	-1.1316
Br	8.2809	-2.2187	-2.8633
N	3.3427	-5.2279	-1.2492
N	1.11	-5.4794	-2.7729
C	3.2121	-4.5029	-2.4149
C	1.988	-4.6234	-3.1564
C	4.573	-5.0097	-0.8172
C	-0.1534	-5.5965	-3.4594
C	4.4006	-3.8367	-2.6568
H	1.82	-3.9852	-4.0297
N	5.2548	-4.1591	-1.6295
C	5.1193	-5.6689	0.4033
C	4.7732	-2.9443	-3.7855
H	5.0611	-6.753	0.2445
H	6.1763	-5.4166	0.5038
C	4.3576	-5.3008	1.6758
H	4.9146	-1.9081	-3.4585
H	3.9943	-2.9462	-4.5511
H	5.7147	-3.2595	-4.2432
H	4.783	-5.8125	2.5434
H	3.3062	-5.5925	1.6033
H	4.3912	-4.2228	1.8621
H	-0.2687	-6.6172	-3.8381
H	-0.2476	-4.892	-4.2946
H	-0.9676	-5.4152	-2.7497
Zn	7.2596	1.8873	1.0263
Br	8.0279	0.5784	2.9347
N	7.9793	-3.7033	0.5838
N	6.0546	-1.7943	0.4132
C	7.6258	-2.9892	1.6632
C	6.5711	-1.9979	1.565
N	2.6866	6.2126	2.8994
N	4.5139	5.044	1.2326
C	4.2871	4.541	2.5008
C	3.3329	5.1915	3.3436
C	8.9818	-4.5105	1.015

continue			
C	4.9907	-0.8147	0.2536
C	8.4662	-3.4184	2.7776
H	6.271	-1.4446	2.4533
C	1.7213	6.8903	3.7275
C	5.4448	4.2697	0.6964
C	5.1032	3.4383	2.6897
H	3.1632	4.8082	4.355
N	9.2874	-4.3537	2.3365
C	9.6842	-5.4864	0.1585
C	5.3444	0.09	-0.9311
H	4.9275	-0.1877	1.157
C	3.6578	-1.5401	0.0427
C	8.4358	-2.9123	4.1623
N	5.8281	3.2753	1.543
C	6.023	4.4513	-0.6675
C	5.2126	2.547	3.8726
H	9.7435	-6.4208	0.7308
H	10.7271	-5.1442	0.0868
C	9.0852	-5.6969	-1.2222
N	6.5629	0.8134	-0.6589
H	5.4904	-0.5625	-1.8103
C	4.1902	1.0455	-1.2344
H	3.7737	-2.2585	-0.7759
H	3.4206	-2.122	0.9404
C	2.5282	-0.573	-0.2914
H	7.4559	-3.0938	4.6186
H	8.5893	-1.827	4.1629
H	9.2058	-3.4037	4.7552
H	7.1054	4.597	-0.5654
H	5.9183	3.5073	-1.216
C	5.4102	5.5995	-1.4547
H	4.9384	1.5179	3.6189
H	4.5519	2.8853	4.6739
H	6.2373	2.5097	4.2507
H	9.6364	-6.475	-1.7539
H	9.1362	-4.7861	-1.8263
H	8.0406	-6.0193	-1.1629
C	7.433	0.965	-1.6263
C	2.8901	0.2962	-1.4889
H	4.4693	1.6584	-2.0982
H	4.0762	1.7311	-0.3835
H	2.3132	0.0654	0.5762
H	1.6135	-1.1367	-0.4939
N	8.7312	2.4644	-0.2551
C	8.5681	1.7736	-1.4466
H	5.881	5.6727	-2.438
H	5.5514	6.5583	-0.946
H	4.3369	5.4535	-1.6201
H	7.2852	0.4607	-2.5834

continue			
H	3.0007	-0.3393	-2.3787
H	2.0858	1.0009	-1.7179
C	9.8835	3.1203	-0.4011
C	9.6766	2.0714	-2.2577
N	10.4865	2.9143	-1.5956
C	10.4678	4.0132	0.6431
C	9.9757	1.5771	-3.6301
C	9.61	4.1669	1.89
H	10.0797	4.8528	2.6002
H	9.4705	3.2068	2.3981
H	8.6206	4.5708	1.6477
H	10.6587	4.9899	0.1817
H	11.4574	3.6208	0.9091
H	9.5285	2.2252	-4.3932
H	11.0559	1.5769	-3.7911
H	9.5948	0.5626	-3.7815
H	0.7431	6.8754	3.2339
H	1.6221	6.4331	4.72
H	2.0158	7.9383	3.8458
Br	-1.572	0.8363	1.7701
C	-1.7417	0.2338	-0.017
C	-0.7937	0.6084	-0.9627
C	-2.8173	-0.5814	-0.3464
C	-0.9154	0.1363	-2.2674
H	0.0214	1.2662	-0.6844
C	-2.9291	-1.0436	-1.6555
H	-3.5571	-0.8423	0.4019
C	-1.979	-0.6946	-2.6121
H	-0.2009	0.4614	-3.0179
H	-3.7876	-1.6485	-1.9286
H	-2.0862	-1.0466	-3.6339
N	-9.8213	-5.1049	-0.332
C	-10.3712	-4.6551	-1.5163
C	-11.6238	-5.2211	-2.0782
C	-8.688	-4.4159	-0.0714
C	-9.5197	-3.6738	-1.953
N	-8.479	-3.5425	-1.0466
C	-7.8957	-4.6048	1.1751
C	-9.6232	-2.8306	-3.1726
H	-6.853	-4.3606	0.9598
H	-7.9369	-5.6641	1.4586
C	-8.3886	-3.7205	2.3238
H	-9.7577	-1.7742	-2.9139
H	-10.4745	-3.1374	-3.7847
H	-8.7118	-2.8887	-3.773
H	-7.7677	-3.8772	3.2098
H	-9.4287	-3.9437	2.5847
H	-8.3099	-2.6686	2.0407
H	-10.2006	-5.8292	0.2559

continue			
H	-11.5294	-6.2923	-2.2911
H	-12.4739	-5.0881	-1.399
H	-11.8713	-4.7207	-3.0159
N	-8.2542	6.2779	0.2843
C	-8.8185	6.0021	1.5148
C	-9.9284	6.8114	2.0788
C	-7.281	5.3796	0.0262
C	-8.1407	4.9057	1.9812
N	-7.1858	4.5368	1.0453
C	-6.4939	5.3519	-1.2398
C	-8.3586	4.1452	3.2392
H	-6.5671	4.342	-1.655
H	-5.4339	5.4925	-1.0024
C	-6.9502	6.3771	-2.2693
H	-8.7565	3.1456	3.0292
H	-9.0729	4.6612	3.8847
H	-7.4211	4.0014	3.7821
H	-6.3645	6.2785	-3.186
H	-6.8186	7.4043	-1.9108
H	-8.0012	6.2345	-2.5429
H	-8.5246	7.027	-0.3316
H	-10.8232	6.7756	1.4467
H	-10.2058	6.4284	3.0622
H	-9.6458	7.8631	2.2023

Table S13. Atomic coordinates of optimized **P-Et⊃*o*-PhF$_2$** models at ground state and S_1 state.

S_0			
Atom	X	Y	Z
Zn	-2.6186	-4.5639	2.7365
Zn	-5.8499	2.8374	1.0973
Zn	-6.9232	-2.0345	-0.9994
Zn	1.8453	-6.6633	-1.0029
Br	-1.8563	-4.0155	4.9148
Br	-5.4045	1.9548	3.2564
Br	2.4388	-8.8745	-1.6485
Br	-6.3079	-1.2577	-3.184
N	-3.9825	-3.303	1.8443
N	-1.311	-5.4937	1.4686
N	-3.512	-6.5207	2.724
N	-4.3996	3.1294	-0.2973
N	-6.8065	1.8623	-0.5304
N	-7.423	-0.699	0.5411
N	-2.8029	3.5031	-1.793
N	-5.4952	-2.643	0.3514
N	3.3288	-5.2775	-1.1874
N	0.3443	-6.511	0.3898

continue			
N	5.2086	-4.162	-1.6112
N	1.0199	-5.4823	-2.6232
C	-4.7613	2.5201	-1.4832
C	-3.2251	3.7008	-0.5176
C	-6.6591	-0.899	1.5679
C	-8.3925	0.3712	0.561
C	-3.7561	2.7463	-2.4137
C	-6.0268	1.8688	-1.5645
C	-8.136	1.2981	-0.6337
C	-5.6712	-1.918	1.5199
C	-4.4718	-3.4547	0.5865
C	-4.7217	-2.3247	2.4439
H	-6.3255	1.388	-2.4977
H	-6.7362	-0.2738	2.4595
C	-3.6536	2.2824	-3.82
C	-4.4935	-1.8359	3.8255
C	-2.5007	4.5	0.5092
C	-3.8956	-4.4164	-0.3981
C	-4.7038	-4.5603	-1.6782
H	-2.8748	-4.0888	-0.6342
H	-3.7764	-5.388	0.0961
H	-4.8974	-2.5331	4.5677
H	-4.9563	-0.8568	3.9666
H	-3.4251	-1.7516	4.0355
C	-3.0913	5.8997	0.6881
H	-2.539	3.9523	1.4558
H	-1.4513	4.5793	0.2185
C	-9.8138	-0.1892	0.5256
H	-8.2676	0.9675	1.4813
C	-9.1856	2.4106	-0.6819
H	-8.2104	0.6947	-1.554
H	-4.2376	-5.2964	-2.3381
H	-4.7671	-3.617	-2.2287
H	-5.7257	-4.9005	-1.4762
C	-10.855	0.9225	0.4642
H	-9.9107	-0.8454	-0.3497
H	-9.9641	-0.8233	1.4063
C	-10.6004	1.8432	-0.7258
H	-10.8153	1.51	1.392
H	-11.8603	0.4918	0.4102
H	-8.9883	3.0424	-1.5557
H	-9.0614	3.0477	0.2038
H	-10.7382	1.2788	-1.6587
H	-11.3306	2.6592	-0.7438
H	-3.8318	3.1018	-4.5264
H	-4.3789	1.4896	-4.0181
H	-2.6481	1.9018	-4.0214
H	-2.5049	6.4715	1.4126
H	-4.122	5.849	1.0552

continue			
H	-3.0846	6.45	-0.2582
C	-1.5505	-6.8565	1.4757
C	3.1521	-4.5435	-2.3455
C	1.9189	-4.6589	-3.05
H	1.7518	-4.0485	-3.9437
C	-0.5135	-7.488	0.8051
C	4.3277	-3.8519	-2.6068
C	-2.7098	-7.3493	2.1432
H	-2.8995	-8.4275	2.1592
C	-0.1734	-5.3278	0.8146
C	4.5686	-5.0285	-0.7851
C	0.4661	-4.0027	0.5618
H	0.5063	-3.8364	-0.523
H	1.5138	-4.0565	0.881
C	5.1576	-5.6444	0.4369
H	5.1533	-6.7335	0.3027
H	6.2	-5.3326	0.5252
C	-0.3006	-8.9382	0.5707
H	-0.2335	-9.1698	-0.4958
H	0.6458	-9.268	1.0086
C	4.6353	-2.9302	-3.7302
H	4.7195	-1.8934	-3.3857
H	3.8517	-2.9718	-4.491
H	5.5969	-3.1773	-4.1863
C	-0.2437	-2.8466	1.2527
H	0.2721	-1.9057	1.0505
H	-0.2676	-2.9855	2.3389
H	-1.2729	-2.7318	0.895
C	4.3951	-5.2765	1.7089
H	4.8566	-5.7499	2.5793
H	3.3503	-5.6032	1.6603
H	4.412	-4.1944	1.8679
H	-1.1112	-9.5227	1.0127
C	-4.6647	-6.9997	3.4423
C	-0.2388	-5.6113	-3.3098
H	-5.5719	-6.5273	3.0463
H	-4.7788	-8.0897	3.3793
H	-4.5811	-6.7141	4.4966
H	-0.3266	-4.9285	-4.1653
H	-0.3607	-6.6398	-3.667
H	-1.0622	-5.4081	-2.6138
Zn	-1.1297	4.352	-2.6613
Br	-0.0524	2.9814	-4.2747
N	0.1414	5.3759	-1.4351
N	1.6881	6.4169	-0.228
N	-1.8257	6.3506	-3.0414
C	-0.0971	6.7352	-1.4979
C	-1.1428	7.2006	-2.348
H	-1.3456	8.2747	-2.4119

continue			
C	0.8662	7.3818	-0.7345
C	1.8018	3.8972	-0.3449
H	2.8904	3.9875	-0.317
H	1.5563	3.2137	-1.1643
C	1.0332	8.8312	-0.4623
H	0.8646	9.0505	0.5979
H	0.3255	9.4222	-1.0486
H	2.0524	9.16	-0.6846
C	1.2164	5.2249	-0.6777
C	1.3123	3.3426	0.9949
H	1.8601	2.4302	1.2486
H	0.246	3.1021	0.9748
H	1.4824	4.0637	1.7975
C	-2.8757	6.797	-3.9202
H	-3.0483	7.8793	-3.8581
H	-2.6269	6.5402	-4.9556
H	-3.8073	6.275	-3.6714
Zn	7.3007	1.8201	1.0944
Zn	6.9264	-3.0744	-1.0623
Br	8.0377	0.6642	3.079
Br	8.0647	-2.2717	-3.0213
N	8.7625	2.4636	-0.1842
N	6.5987	0.8255	-0.6213
N	7.9551	-3.8105	0.5315
N	6.0703	-1.823	0.4141
N	10.5466	2.9005	-1.4926
N	9.1794	-4.5665	2.2634
C	8.6315	1.7512	-1.3664
C	7.5903	-3.107	1.6686
C	6.6114	-2.0901	1.5677
H	6.3363	-1.5237	2.4596
C	7.4972	0.9378	-1.5629
H	7.382	0.3963	-2.5042
C	9.9177	3.1203	-0.316
C	5.383	0.097	-0.89
H	5.5303	-0.5501	-1.7721
C	2.9291	0.3398	-1.4381
H	3.032	-0.3109	-2.3181
H	2.1387	1.0591	-1.6823
C	5.0426	-0.818	0.2923
H	5.0082	-0.1911	1.2017
C	4.2413	1.0694	-1.1923
H	4.5195	1.6803	-2.0586
H	4.1372	1.755	-0.3394
C	3.6785	-1.4919	0.1014
H	3.7694	-2.2205	-0.7119
H	3.4433	-2.0661	1.0051
C	2.5611	-0.5107	-0.2281
H	2.3727	0.1448	0.635

continue			
H	1.6312	-1.0584	-0.4152
C	9.7543	2.0376	-2.1562
C	10.4837	4.0215	0.7326
C	10.0858	1.5127	-3.5103
H	10.7347	4.9772	0.256
H	11.4424	3.6	1.0609
C	9.5743	4.2398	1.9328
H	9.7182	2.1801	-4.2994
H	9.6509	0.5216	-3.6711
H	11.1705	1.4424	-3.6209
H	10.0318	4.9346	2.6432
H	9.3823	3.3007	2.4622
H	8.6091	4.6622	1.6313
C	8.9066	-4.6554	0.9458
C	9.619	-5.591	0.0253
C	8.3656	-3.594	2.7281
H	10.2162	-6.2651	0.6455
H	10.3243	-5.0142	-0.588
C	8.6877	-6.3787	-0.8924
H	9.2576	-7.0342	-1.5576
H	8.092	-5.7122	-1.5245
H	7.9959	-7.0007	-0.3154
C	8.3612	-3.1644	4.1541
H	7.5395	-3.6281	4.7139
H	8.2562	-2.078	4.2361
H	9.2982	-3.467	4.6261
Zn	3.3892	6.6735	0.9241
Br	4.4322	8.8047	0.7128
N	2.7301	6.2176	2.9155
N	4.5369	5.0081	1.2511
N	5.8278	3.2232	1.5764
C	4.2936	4.5096	2.5199
C	3.3622	5.1828	3.3606
H	3.1963	4.8125	4.3775
C	5.0962	3.3936	2.7133
C	6.0601	4.3921	-0.6302
H	7.1389	4.5518	-0.5135
H	5.9808	3.4371	-1.1625
C	5.4656	4.2198	0.7263
C	5.1694	2.4959	3.8927
H	4.8667	1.4778	3.6271
H	4.515	2.8519	4.6922
H	6.1929	2.4157	4.2674
C	5.4402	5.5188	-1.4416
H	5.9143	5.5777	-2.4244
H	5.5704	6.4895	-0.9523
H	4.3682	5.3587	-1.6082
C	1.8033	6.9286	3.7578
H	2.1362	7.9655	3.8764

continue			
H	0.8157	6.9531	3.2821
H	1.7027	6.4735	4.7516
N	-7.0559	4.6035	1.1749
N	-8.0097	6.4696	0.5738
C	-7.1402	5.5079	0.2048
C	-7.9152	5.0044	2.1904
C	-8.1146	4.2159	3.4339
C	-6.4622	5.4822	-1.1234
C	-6.6127	6.7715	-1.9197
H	-6.8616	4.6359	-1.6956
H	-5.405	5.2542	-0.968
H	-8.5089	3.2184	3.211
H	-8.8241	4.7175	4.0953
H	-7.1756	4.066	3.9718
H	-6.0814	6.689	-2.8703
H	-6.1918	7.6274	-1.382
C	-8.5173	6.1799	1.8245
H	-7.6595	6.9889	-2.1605
C	-9.4975	7.0626	2.5064
H	-9.0896	8.0647	2.6799
H	-10.4217	7.1711	1.9277
H	-9.7644	6.6455	3.4787
H	-8.2394	7.2791	0.0201
N	-8.5295	-3.4101	-1.0776
N	-9.8644	-5.0658	-0.6055
C	-9.5866	-3.3909	-1.9791
C	-7.9101	-4.8261	0.9277
H	-6.8725	-4.5451	0.7377
H	-7.9348	-5.9174	1.0294
C	-9.7038	-2.3768	-3.0596
H	-9.7644	-1.3607	-2.6533
H	-10.6043	-2.5513	-3.6521
H	-8.8357	-2.3996	-3.7234
C	-8.7254	-4.4345	-0.2548
C	-8.3917	-4.1629	2.2212
H	-7.7388	-4.4372	3.0539
H	-8.3725	-3.075	2.1226
H	-9.413	-4.4656	2.4719
C	-10.4327	-4.4296	-1.6908
C	-11.6921	-4.9038	-2.3182
H	-11.9778	-4.2406	-3.1359
H	-11.5839	-5.9117	-2.7341
H	-12.5211	-4.9222	-1.602
H	-10.2351	-5.8813	-0.1436
C	-1.7685	0.7522	0.0086
C	-0.6521	0.7023	-0.8063
C	-2.9279	0.076	-0.3494
C	-0.6953	-0.04	-1.9843
H	0.2385	1.2431	-0.5101

continue			
C	-2.9793	-0.6719	-1.5126
C	-1.8536	-0.7284	-2.3324
H	0.1718	-0.0519	-2.6353
H	-3.9091	-1.1578	-1.7876
H	-1.8967	-1.2961	-3.2563
F	-4.0057	0.1791	0.4407
F	-1.7569	1.4741	1.1383

S_1			
Atom	X	Y	Z
Zn	-2.5795	-4.6062	2.744
Zn	-5.9082	2.7658	1.169
Zn	-6.9487	-2.1088	-0.9381
Zn	-1.2426	4.3482	-2.6296
Zn	3.2859	6.7043	0.9209
Br	-1.3716	-4.0253	4.7248
Br	-5.3209	2.268	3.408
Br	-6.0635	-1.4708	-3.1344
Br	4.3387	8.8367	0.7908
Br	-0.0114	3.0206	-4.1908
N	-4.0536	-3.5087	1.9044
N	-4.4257	3.0452	-0.2255
N	-6.8292	1.7815	-0.4808
N	-7.4809	-0.8394	0.5222
N	-2.8412	3.4446	-1.7273
N	-5.5871	-2.8384	0.4343
N	0.0867	5.4015	-1.4688
N	1.6265	6.4436	-0.2567
N	-2.0182	6.3464	-2.8922
C	-4.7346	2.3546	-1.3789
C	-3.2857	3.6757	-0.4652
C	-6.6482	-0.9844	1.5988
C	-8.4253	0.2314	0.5396
C	-3.7447	2.5952	-2.3148
C	-5.9958	1.6774	-1.4605
C	-8.1553	1.2106	-0.6124
C	-5.69	-2.0225	1.5671
C	-4.5943	-3.7073	0.6894
C	-4.729	-2.443	2.4788
H	-6.2442	1.1064	-2.358
H	-6.7081	-0.3111	2.4475
C	-3.6155	2.077	-3.701
C	-4.4361	-1.8843	3.8242
C	-2.606	4.54	0.5423
C	-4.097	-4.7379	-0.2765
C	-4.9743	-4.9151	-1.5076
H	-3.0808	-4.4686	-0.5957
H	-3.9888	-5.6952	0.2486
H	-5.126	-2.2614	4.5918
H	-4.5328	-0.7936	3.8075

continue			
H	-3.4231	-2.1288	4.1521
C	-3.3298	5.8663	0.7723
H	-2.555	3.981	1.4819
H	-1.5788	4.7242	0.2183
C	-9.8637	-0.2895	0.4514
H	-8.3304	0.8098	1.4785
C	-9.1962	2.3314	-0.6633
H	-8.2064	0.6348	-1.5522
H	-4.5726	-5.7021	-2.1528
H	-5.03	-3.9952	-2.0973
H	-5.9975	-5.1963	-1.2381
C	-10.9011	0.8248	0.3919
H	-9.942	-0.9225	-0.444
H	-10.0391	-0.9496	1.3074
C	-10.6104	1.7719	-0.7684
H	-10.8806	1.3937	1.3325
H	-11.9102	0.4063	0.3026
H	-8.9731	2.996	-1.5075
H	-9.1072	2.9367	0.2481
H	-10.7176	1.2268	-1.7171
H	-11.3371	2.5922	-0.7941
H	-3.8804	2.839	-4.4446
H	-4.2678	1.212	-3.8452
H	-2.5848	1.7724	-3.9004
H	-2.7965	6.4735	1.5095
H	-4.3439	5.6981	1.1489
H	-3.3956	6.4452	-0.1553
C	-0.23	6.7436	-1.4228
C	-1.3574	7.1924	-2.1764
H	-1.6449	8.2482	-2.1395
C	0.7277	7.3928	-0.6565
C	1.8846	3.9544	-0.571
H	2.9657	4.1105	-0.6135
H	1.6183	3.3116	-1.4166
C	0.8292	8.8261	-0.281
H	0.648	8.9636	0.7913
H	0.0947	9.4245	-0.8251
H	1.8309	9.2172	-0.4811
C	1.202	5.2611	-0.7747
C	1.5177	3.2959	0.7589
H	2.1291	2.4045	0.9268
H	0.4698	2.9952	0.7833
H	1.6953	3.9786	1.5925
C	-3.1761	6.7659	-3.6393
H	-3.4119	7.8281	-3.4939
H	-3.0153	6.5831	-4.7072
H	-4.0398	6.1656	-3.3317
Zn	7.2521	1.8938	1.0313
Br	7.9971	0.5401	2.9123

continue			
N	8.7382	2.4905	-0.2233
N	2.6539	6.1975	2.9114
N	4.5001	5.0556	1.2452
N	6.5538	0.8708	-0.6935
N	7.9295	-3.5991	0.6062
N	5.9888	-1.7177	0.3704
N	5.8279	3.2947	1.5526
N	10.5134	2.9512	-1.5335
N	9.1289	-4.2945	2.4182
C	8.5814	1.8284	-1.4316
C	4.28	4.5503	2.5137
C	7.5072	-2.9155	1.6803
C	3.3206	5.1904	3.3582
H	3.1631	4.811	4.3729
C	6.4494	-1.9315	1.5435
H	6.0974	-1.398	2.4245
C	7.4408	1.0359	-1.6435
H	7.3027	0.5546	-2.6138
C	5.1035	3.4526	2.7002
C	9.8991	3.1371	-0.3417
C	6.0232	4.4839	-0.6506
H	7.1098	4.5853	-0.5411
H	5.8856	3.5597	-1.2253
C	5.4371	4.2888	0.7081
C	5.221	2.5611	3.8822
H	4.9433	1.5323	3.6311
H	4.5674	2.9001	4.6891
H	6.2487	2.5225	4.2524
C	5.4586	5.6765	-1.4073
H	5.95	5.7705	-2.3787
H	5.6147	6.6119	-0.8611
H	4.3849	5.5696	-1.5989
C	5.3392	0.156	-1.0104
H	5.517	-0.5038	-1.8785
C	2.9147	0.3617	-1.6824
H	3.0712	-0.2816	-2.5598
H	2.1225	1.0632	-1.9605
C	4.9319	-0.7398	0.1629
H	4.8306	-0.1079	1.0594
C	4.2026	1.1126	-1.3711
H	4.5214	1.72	-2.2251
H	4.0499	1.8034	-0.5307
C	3.6087	-1.4644	-0.1037
H	3.7616	-2.1882	-0.9116
H	3.3298	-2.0409	0.7854
C	2.4967	-0.4984	-0.496
H	2.2434	0.1465	0.3556
H	1.5915	-1.0644	-0.7334
C	9.7024	2.1318	-2.2232

continue			
C	10.4805	4.0008	0.7283
C	10.0121	1.6638	-3.6025
H	10.6844	4.986	0.2911
H	11.4638	3.5938	0.9953
C	9.6109	4.134	1.9695
H	9.5825	2.334	-4.3566
H	9.6204	0.6578	-3.7805
H	11.094	1.6532	-3.7506
H	10.0775	4.8021	2.6986
H	9.4606	3.1644	2.4558
H	8.6265	4.5488	1.7252
C	8.9061	-4.4163	1.0768
C	9.6611	-5.3684	0.2385
C	8.2772	-3.3742	2.8328
H	9.7346	-6.3014	0.8105
H	10.6941	-4.9928	0.1914
C	9.1032	-5.5928	-1.1576
H	9.6856	-6.3583	-1.6741
H	9.1499	-4.6818	-1.7618
H	8.0647	-5.9382	-1.1244
C	8.1514	-2.9157	4.2289
H	7.1458	-3.1222	4.6131
H	8.2948	-1.8304	4.276
H	8.8858	-3.4215	4.8541
C	1.686	6.8672	3.7431
H	1.9654	7.9208	3.8489
H	0.7031	6.832	3.2602
H	1.6045	6.4175	4.7405
Zn	1.8697	-6.6404	-1.0485
Br	2.9625	-8.7455	-1.2803
N	-1.3074	-5.5485	1.4019
N	-3.2497	-6.6541	2.9718
N	0.2979	-6.5019	0.2032
C	-1.4347	-6.919	1.5064
C	-0.4359	-7.5154	0.7584
C	-2.4749	-7.4557	2.3331
H	-2.5879	-8.5424	2.4126
C	-0.2686	-5.3358	0.6181
C	0.2167	-3.9846	0.2019
H	0.0401	-3.8681	-0.8766
H	1.3067	-3.9504	0.3251
C	-0.1481	-8.9569	0.5426
H	-0.2614	-9.2344	-0.5105
H	0.8811	-9.2042	0.816
C	-0.4472	-2.8429	0.9589
H	-0.0745	-1.8779	0.6069
H	-0.2585	-2.9143	2.0347
H	-1.5291	-2.8373	0.8004
H	-0.8269	-9.5765	1.1332

continue			
C	-4.2876	-7.1601	3.8318
H	-5.252	-6.7441	3.5217
H	-4.3492	-8.2563	3.8217
H	-4.1003	-6.8227	4.8568
Zn	6.9102	-2.9954	-1.1427
Br	8.269	-2.1482	-2.8794
N	3.3568	-5.2052	-1.2481
N	5.2613	-4.1204	-1.624
N	1.1947	-5.6062	-2.8359
C	3.2482	-4.54	-2.4505
C	2.0598	-4.7416	-3.2305
H	1.91	-4.1625	-4.1472
C	4.4311	-3.8615	-2.6882
C	4.5701	-4.945	-0.7922
C	5.0852	-5.5461	0.4709
H	5.0393	-6.6362	0.3541
H	6.1366	-5.282	0.5926
C	4.8168	-3.0055	-3.8403
H	4.9507	-1.9594	-3.5425
H	4.0481	-3.0338	-4.6156
H	5.7656	-3.3304	-4.2755
C	4.279	-5.1353	1.7025
H	4.6728	-5.6173	2.6014
H	3.2304	-5.4288	1.6022
H	4.3049	-4.0518	1.8542
C	-0.0269	-5.8141	-3.5745
H	-0.1059	-5.1622	-4.453
H	-0.0821	-6.8582	-3.899
H	-0.8833	-5.6294	-2.9172
N	-7.1734	4.5155	1.1133
N	-8.2952	6.2058	0.3123
C	-7.308	5.3193	0.0667
C	-8.1186	4.8989	2.0541
C	-8.2967	4.1878	3.3467
C	-6.5551	5.2633	-1.2194
C	-7.0266	6.2768	-2.2534
H	-6.6497	4.2483	-1.6172
H	-5.4882	5.395	-1.0128
H	-8.6242	3.1548	3.1856
H	-9.0492	4.6883	3.9602
H	-7.3585	4.1308	3.9035
H	-6.4603	6.1609	-3.1803
H	-6.8822	7.308	-1.9118
C	-8.8293	5.9632	1.5625
H	-8.0837	6.137	-2.5049
C	-9.9436	6.7712	2.1194
H	-9.6756	7.8302	2.2095
H	-10.8462	6.7043	1.501
H	-10.2019	6.4125	3.1171

continue			
H	-8.5938	6.926	-0.3248
N	-8.5948	-3.4527	-1.2108
N	-10.0929	-4.9592	-0.7247
C	-9.5896	-3.4059	-2.1745
C	-8.2204	-4.764	0.9176
H	-7.1487	-4.6115	0.7698
H	-8.3823	-5.8314	1.112
C	-9.5591	-2.4322	-3.2975
H	-9.6961	-1.4065	-2.9343
H	-10.3553	-2.6412	-4.0157
H	-8.5959	-2.4566	-3.8137
C	-8.9255	-4.3962	-0.3409
C	-8.6773	-3.9215	2.1121
H	-8.1323	-4.2213	3.011
H	-8.4636	-2.8661	1.9282
H	-9.7494	-4.0416	2.3009
C	-10.5384	-4.3508	-1.882
C	-11.7989	-4.7503	-2.5575
H	-11.9466	-4.1498	-3.4568
H	-11.7839	-5.803	-2.8626
H	-12.6736	-4.6004	-1.9139
H	-10.5593	-5.7043	-0.2336
C	-1.6452	0.8527	0.0426
C	-0.8642	0.7771	-1.0955
C	-2.7664	0.0467	0.1948
C	-1.2051	-0.136	-2.0927
H	-0.0205	1.4473	-1.2104
C	-3.1129	-0.8598	-0.792
C	-2.3242	-0.9503	-1.9379
H	-0.6108	-0.1678	-3.0002
H	-3.9998	-1.469	-0.6582
H	-2.6225	-1.6379	-2.7228
F	-3.5044	0.1935	1.2922
F	-1.36	1.7379	1.0136

References

[1] R. Paul, J. A. Brockman, W. A. Hallett, J. W. Hanifin, M. E. Tarrant, L. W. Torley, F. M. Callahan, P. F. Fabio, B. D. Johnson, *J. Med. Chem.* **1985**, *28*, 1704.
[2] G. M. Sheldrick, *Acta Crystallogr. A Found. Adv.* **2015**, *71*, 3.
[3] G. M. Sheldrick, *Acta Crystallogr. C Struct. Chem.* **2015**, *71*, 3.
[4] A. Spek, *J. Appl. Crystallogr.* **2003**, *36*, 7.
[5] S. J. Clark, M. D. Segall, C. J. Pickard, P. J. Hasnip, M. I. Probert, K. Refson, M. C. Payne, *Z. Kristallogr. Cryst. Mater.* **2005**, *220*, 567.
[6] D. Vanderbilt, *Phys. Rev. B* **1990**, *41*, 7892.
[7] J. P. Perdew, K. Burke, M. Ernzerhof, *Phys. Rev. Lett.* **1996**, *77*, 3865.
[8] C. Adamo, V. Barone, *J. Chem. Phys.* **1999**, *110*, 6158.
[9] A. D. Becke, E. R. Johnson, *J. Chem. Phys.* **2005**, *123*, 154101.
[10] S. Grimme, J. Antony, S. Ehrlich, H. Krieg, *J. Chem. Phys.* **2010**, *132*, 154104.
[11] E. R. Johnson, A. D. Becke, *J. Chem. Phys.* **2005**, *123*, 24101.
[12] P. J. Hay, W. R. Wadt, *J. Chem. Phys.* **1985**, *82*, 270.
[13] M. M. Francl, W. J. Pietro, W. J. Hehre, J. S. Binkley, M. S. Gordon, D. J. DeFrees, J. A. Pople, *J. Chem. Phys.* **1982**, *77*, 3654.
[14] T. Lu, Q. Chen, *J. Comput. Chem.* **2022**, *43*, 539.
[15] Y. Zhao, D. G. Truhlar, *Theor. Chem. Acc.* **2008**, *120*, 215.
[16] A. Schäfer, C. Huber, R. Ahlrichs, *J. Chem. Phys.* **1994**, *100*, 5829.
[17] T. Lu, F. Chen, *J. Comput. Chem.* **2012**, *33*, 580.
[18] W. Humphrey, A. Dalke, K. Schulten, *J. Mol. Graph. Model.* **1996**, *14*, 33.
[19] M. Frisch, G. Trucks, H. Schlegel, G. Scuseria, M. Robb, J. Cheeseman, G. Scalmani, V. Barone, B. Mennucci, G. Petersson, *Wallingford CT*.
[20] M. v. Hopffgarten, G. Frenking, *Wiley Interdiscip. Rev. Comput. Mol. Sci.* **2011**, *2*, 43.
[21] L. Zhao, M. von Hopffgarten, D. M. Andrada, G. Frenking, *Wiley Interdiscip. Rev. Comput. Mol. Sci.* **2017**, *8*, e1345.
[22] M. P. Mitoraj, A. Michalak, T. Ziegler, *J. Chem. Theory Comput.* **2009**, *5*, 962.
[23] C. Fonseca Guerra, J. Snijders, G. t. Te Velde, E. J. Baerends, *Theor. Chem. Acc.* **1998**, *99*, 391.
[24] G. t. Te Velde, F. M. Bickelhaupt, E. J. Baerends, C. Fonseca Guerra, S. J. van Gisbergen, J. G. Snijders, T. Ziegler, *J. Comput. Chem.* **2001**, *22*, 931.
[25] E. v. Van Lenthe, J. Snijders, E. Baerends, *J. Chem. Phys.* **1996**, *105*, 6505.
[26] E. v. Lenthe, E.-J. Baerends, J. G. Snijders, *J. Chem. Phys.* **1993**, *99*, 4597.
[27] E. van Lenthe, E.-J. Baerends, J. G. Snijders, *J. Chem. Phys.* **1994**, *101*, 9783.
[28] S. Grimme, J. Antony, S. Ehrlich, H. Krieg, *J. Chem. Phys.* **2010**, *132*, 154104.
[29] E. Van Lenthe, E. J. Baerends, *J. Comput. Chem.* **2003**, *24*, 1142.
[30] P. F. Gao, Y. Y. Jiang, H. Liu, M. S. Zhou, T. Li, H. R. Fu, L. F. Ma, D. S. Li, *ACS Appl. Mater. Interfaces* **2022**, *14*, 16435.
[31] H. R. Fu, N. Wang, X. X. Wu, F. F. Li, Y. Zhao, L. F. Ma, M. Du, *Adv. Optical Mater.* **2020**, *8*, 2000330.
[32] W. Shang, X. Zhu, T. Liang, C. Du, L. Hu, T. Li, M. Liu, *Angew. Chem. Int. Ed.* **2020**, *59*, 12811.
[33] T. Zhao, J. Han, X. Jin, M. Zhou, Y. Liu, P. Duan, M. Liu, *Research* **2020**, *2020*, 6452123.
[34] L. Hu, K. Li, W. Shang, X. Zhu, M. Liu, *Angew. Chem. Int. Ed.* **2020**, *59*, 4953.
[35] H. Jiang, W. Zhang, B. Hou, Y. Liu, Y. Cui, *CCS Chem.* **2022**, 1.
[36] C. Zhang, Z. P. Yan, X. Y. Dong, Z. Han, S. Li, T. Fu, Y. Y. Zhu, Y. X. Zheng, Y. Y. Niu, S. Q. Zang, *Adv. Mater.* **2020**, *32*, 2002914.
[37] X. Z. Wang, M. Y. Sun, Z. Huang, M. Xie, R. Huang, H. Lu, Z. Zhao, X. P. Zhou, D. Li, *Adv. Optical Mater.* **2021**, *9*, 2002096.
